# Mericitabine and Either Boceprevir or Telaprevir in Combination with Peginterferon Alfa-2a plus Ribavirin for Patients with Chronic Hepatitis C Genotype 1 Infection and Prior Null Response: The Randomized DYNAMO 1 and DYNAMO 2 Studies

**DOI:** 10.1371/journal.pone.0145409

**Published:** 2016-01-11

**Authors:** Heiner Wedemeyer, Xavier Forns, Christophe Hézode, Samuel S. Lee, Astrid Scalori, Athina Voulgari, Sophie Le Pogam, Isabel Nájera, James A. Thommes

**Affiliations:** 1 Department of Gastroenterology, Hepatology and Endocrinology at Hannover Medical School, Hannover, Germany; 2 Liver Unit, Hospital Clinic, IDIBAPS and CIBEREHD, Barcelona, Spain; 3 Department of Hepatology and Gastroenterology, Hôpital Henri Mondor, INSERM U955, Université Paris-Est, Créteil, France; 4 Liver Unit, University of Calgary, Calgary, Canada; 5 Global Product Development Immunology, Respiratory, Roche Products Ltd, Welwyn, United Kingdom; 6 Global Product Development Clinical Science, Roche Products Ltd, Welwyn, United Kingdom; 7 Clinical Development-Infectious Diseases, Genentech Inc., South San Francisco, California, United States of America; 8 Roche Pharma and Early Development, Roche Innovation Center Basel, F. Hoffmann-La Roche Ltd, Basel, Switzerland; 9 Product Development Immunology, Genentech Inc., South San Francisco, California, United States of America; Taipei Veterans General Hosptial, TAIWAN

## Abstract

Most patients with chronic hepatitis C virus (HCV) genotype 1 infection who have had a previous null response (<2-log_10_ reduction in HCV RNA by treatment week 12) to peginterferon/ribavirin (PegIFN/RBV) do not achieve a sustained virological response (SVR) when re-treated with a first-generation HCV protease inhibitor (PI) administered in combination with PegIFN/RBV. We studied the incremental benefits associated with adding mericitabine (nucleoside analog inhibitor of HCV polymerase) to PI plus PegIFN alfa-2a/RBV-based therapy in two double-blind randomized multicenter phase 2 trials (with boceprevir in DYNAMO 1, and with telaprevir in DYNAMO 2). The primary endpoint in both trials was SVR, defined as HCV RNA <25 IU/mL 12 weeks after the end of treatment (SVR12). Overall, the addition of mericitabine to PI plus PegIFN alfa-2a/RBV therapy resulted in SVR12 rates of 60–70% in DYNAMO 1 and of 71–96% in DYNAMO 2. SVR12 rates were similar in patients infected with HCV genotype 1a and 1b in both trials. The placebo control arms in both studies were stopped because of high rates of virological failure. Numerically lower relapse rates were associated with longer treatment with mericitabine (24 versus 12 weeks), telaprevir-containing regimens, and regimens that included 48 weeks of PegIFN alfa-2a/RBV therapy. No mericitabine resistance mutations were identified in any patient in either trial. The addition of mericitabine did not add to the safety burden associated with either telaprevir or boceprevir-based regimens. These studies demonstrate increased SVR rates and reduced relapse rates in difficult-to-treat patients when a nucleoside polymerase inhibitor with intermediate antiviral potency is added to regimens containing a first-generation PI.

***Trial Registration***: ClinicalTrials.gov NCT01482403 and ClinicalTrials.gov NCT01482390

## Introduction

Chronic hepatitis C virus (HCV) infection is estimated to affect approximately 180 million people worldwide and is a leading cause of hepatocellular carcinoma, cirrhosis, and liver-related death [1, 2]. The goal of antiviral treatment is attainment of sustained virological response (SVR), which is associated with long-term eradication of the virus and interruption of liver disease progression [3, 4]. Compared with treated patients not attaining SVR, patients who achieve SVR demonstrate significant reductions in both liver-related and overall mortality [5, 6].

The first direct-acting antiviral agents (DAAs) approved for treatment of chronic HCV infection were the serine protease inhibitors boceprevir (BOC) and telaprevir (TVR), both of which are only effective against HCV genotype 1 and must be used in combination with peginterferon alfa/ribavirin [7, 8]. Compared with dual peginterferon alfa/ribavirin therapy, these protease inhibitor-based triple combinations increase SVR rates in treatment-naïve and previously treated patients and also decrease the required duration of treatment for most previously untreated patients [9–13]. Compassionate use and expanded access studies in patients with more advanced liver disease have demonstrated the effectiveness of BOC- and TVR-based triple therapies, but have also shown high rates of serious adverse events including anaemia, especially in patients with advanced cirrhosis [14–16].

The SVR rates attained with BOC- and TVR-based triple therapies are lowest (29−38%) among patients with a prior null response to peginterferon alfa/ribavirin (defined as <2 log_10_ decline in HCV RNA by week 12) [12, 13]. One approach to increasing SVR rates among prior null responders to dual peginterferon alfa/ribavirin therapy is to incorporate a second DAA into a protease-inhibitor-based regimen (quadruple therapy) [17, 18]. Mericitabine (MCB) is an investigational nucleoside analog inhibitor of the HCV RNA-dependent RNA polymerase that has a high barrier to resistance, and is effective against HCV that contains mutations that confer resistance to protease inhibitors [19–21]. MCB has been evaluated in combination with peginterferon alfa-2a/ribavirin, with or without ritonavir-boosted danoprevir (danoprevir/r, a second-generation protease inhibitor), and in interferon-free regimens with danoprevir/r and setrobuvir [18, 22–25]. Of particular note, 24 weeks of treatment with a four-drug regimen consisting of MCB, danoprevir/r, and peginterferon alfa-2a/ribavirin demonstrated an SVR rate of 84% in patients with prior null response to peginterferon/ribavirin [18].

This report summarizes the results of two randomized studies that evaluated the efficacy and safety of MCB and either BOC (DYNAMO 1) or TVR (DYNAMO 2) in combination with peginterferon alfa-2a/ribavirin and among patients with chronic HCV genotype 1 infection who had prior null response to treatment with peginterferon alfa/ribavirin.

## Methods

### Study designs

DYNAMO 1 and DYNAMO 2 (clinicaltrials.gov identifiers: NCT01482403 and NCT01482390, respectively) were both randomized, double-blind, international, multicenter, parallel-group, Phase 2 studies designed to evaluate the efficacy and safety of MCB in combination with peginterferon alfa-2a (PEGASYS^®^, Roche, Basel, Switzerland) and ribavirin (COPEGUS^®^, Roche, Basel, Switzerland) and either BOC (DYNAMO 1) or TVR (DYNAMO 2) in patients with chronic HCV genotype 1 infection and prior null response to peginterferon alfa/ribavirin. The first patients were screened for inclusion in DYNAMO 1 and 2 on November 11 and 14, 2011, respectively, and the last patient visits occurred on January 27 and 28, 2014.

**Fig 1:**
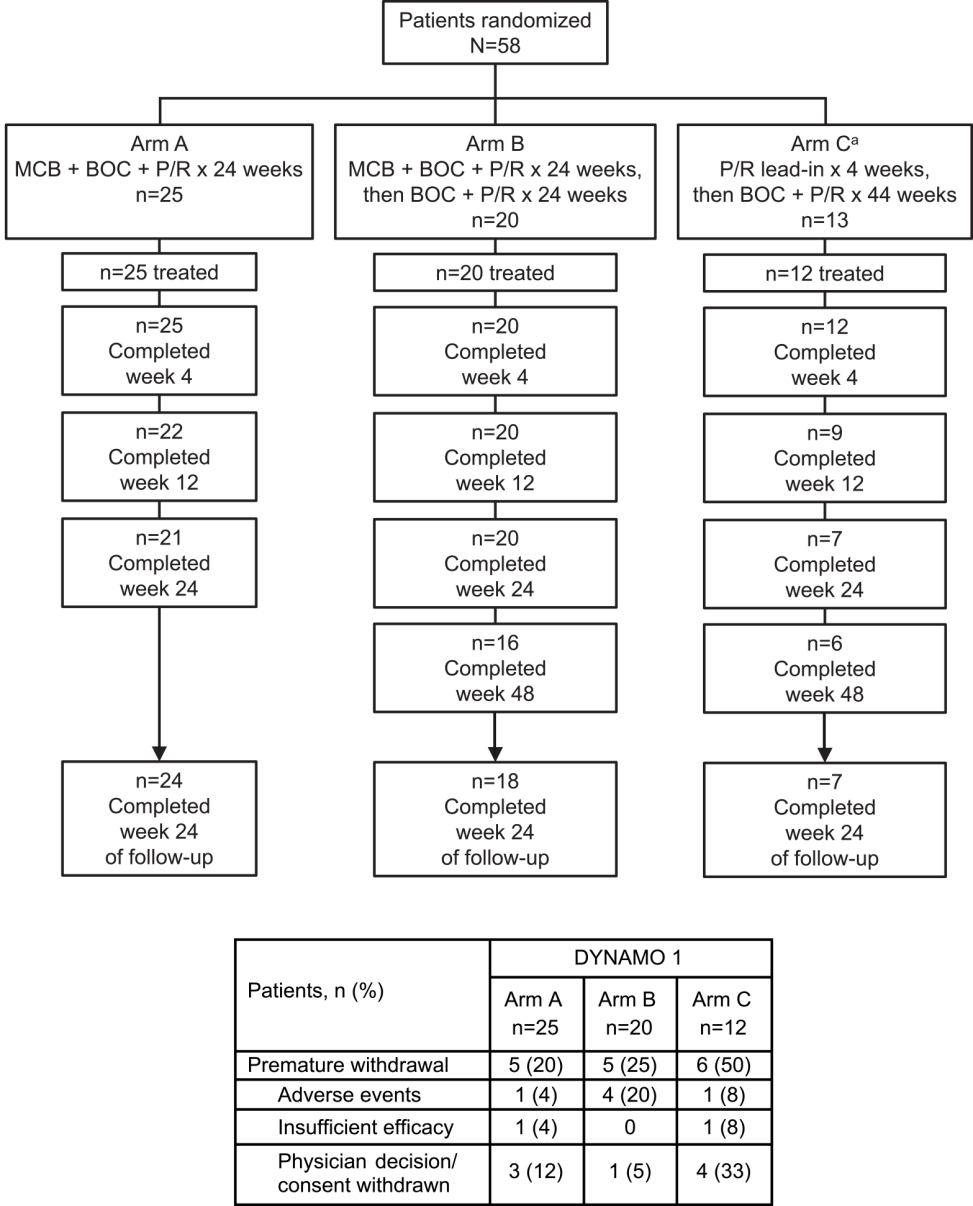
Patient disposition in DYNAMO 1. BOC, boceprevir; MCB, mericitabine; P/R, peginterferon alfa-2a + ribavirin.

**Fig 2:**
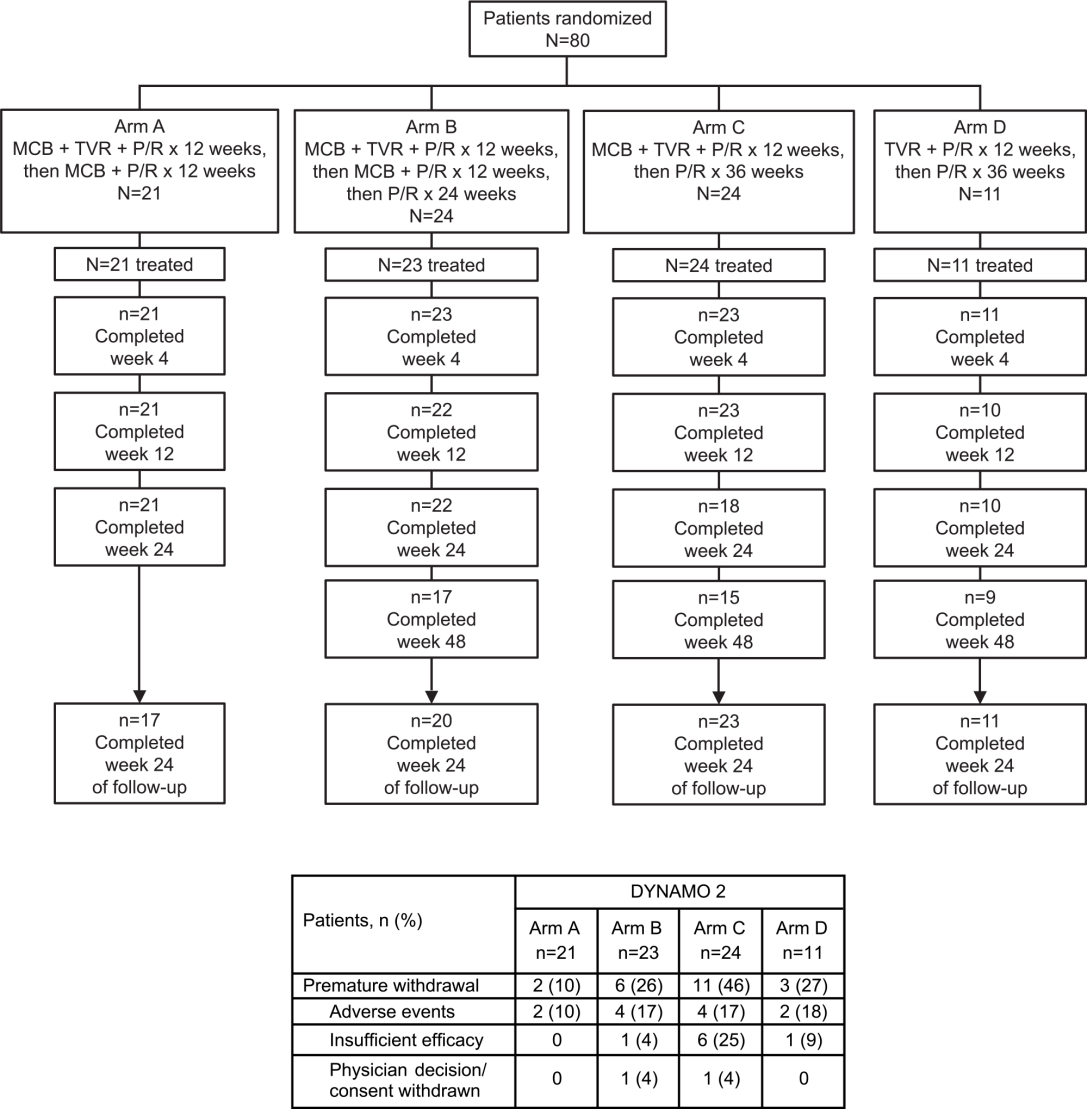
Patient disposition in DYNAMO 2. MCB, mericitabine; P/R, peginterferon alfa-2a + ribavirin; TVR, telaprevir.

DYNAMO 1 recruited 58 patients (Fig 1) at 20 primary specialist clinics in Canada, France, Germany, Spain, and the US; DYNAMO 2 recruited 80 patients (Fig 2) at 31 primary specialist clinics in Canada, France, Germany, Great Britain, Spain, and the US. Study protocols are available online (please see S2 File. NV27780F protocol and S3 File. NV27779F protocol).

Both studies were conducted in accordance with the principles of Good Clinical Practice and the Declaration of Helsinki and all patients gave written informed consent before undergoing any study procedures. Study protocols were approved by independent ethics committees in each country. *Canada*: Conjoint Health Research Ethics Board, Calgary, Alberta; University of Western Ontario (HSREB), London, Ontario. *Germany*: Ethik-Kommission der Medizinischen Hochschule Hannover, Hannover. *France*: CPP Sud- Méditerranée I, Marseille. *Spain*: CEIC Hospital Clinic I Provincial, Barcelona; CEIC Hospitals Vall D'Hebron, Barcelona; CEIC Hospital General Universitario de Valencia, Valencia. *UK*: KHP Clinical Trials Office, London; Joint Research Office, Academic Health Science Centre, Imperial College, London; St George’s Research Office, St George’s University of London, London; RM & G Office, Bournemouth University, Bournemouth. *USA*: Western International Review Board, Olympia, WA; QUORUM review IRB, Seattle, WA UCSD Human Research Protections Program, La Jolla, CA; McGuire VA Institutional Review Board, Richmond, VA; Kaiser Foundation Research Institute, Oakland, CA; Baylor Research Institute Institutional Review Board, Dallas, TX; Weill Cornell Med College IRB, New York, NY; Saint Louis University IRB, St Louis, MO; Human Studies Subcommittee VA Long Beach, Long Beach, CA; Metrowest Medical Center IRB, Framingham, MA; WIRB, Puyallup, WA; Henry Ford Health System IRB, Detroit, MI; LSU Health Sciences Center IRB, New Orleans LA; KC-VAMC Human Committee, Kansas City, MO.

### Patients

Adult patients with chronic HCV genotype 1a or 1b infection, serum HCV RNA level ≥50,000 IU/mL, a liver biopsy result consistent with the diagnosis of chronic hepatitis C and a prior null response to peginterferon alfa/ribavirin were eligible for inclusion in the studies. Null response to prior therapy was defined as a <2-log_10_ reduction in HCV RNA after at least 12 weeks of treatment. Previous HCV treatment must have been discontinued at least 12 weeks prior to enrollment.

Patients without cirrhosis/transition to cirrhosis must have had a biopsy within the previous 24 months. In contrast, the liver biopsy may have been obtained at any time in the past in patients with cirrhosis/transition to cirrhosis. The diagnosis could be confirmed by FibroScan performed within the previous 12 months, on which elasticity scores ≥12.5 kPa were used to designate cirrhosis/transition to cirrhosis. A repeat liver biopsy was required to determine cirrhosis status in patients with elasticity scores ≥9.5 kPa but <12.5 kPa. Patients with cirrhosis/transition to cirrhosis must have had an abdominal ultrasound, computerized tomography scan, or magnetic resonance imaging scan to confirm the absence of hepatocellular carcinoma within the previous 6 months, endoscopy without evidence of gastroesophageal bleeding within the previous 24 months, and a serum alfa-fetoprotein level <100 ng/mL. Patients were excluded if they had received prior treatment with any DAA, were coinfected with hepatitis A or B virus or human immunodeficiency virus, had a medical condition associated with liver disease other than HCV infection, a history or evidence of decompensated liver disease and/or serious coexisting chronic medical or psychiatric conditions.

### Treatment

**Fig 3:**
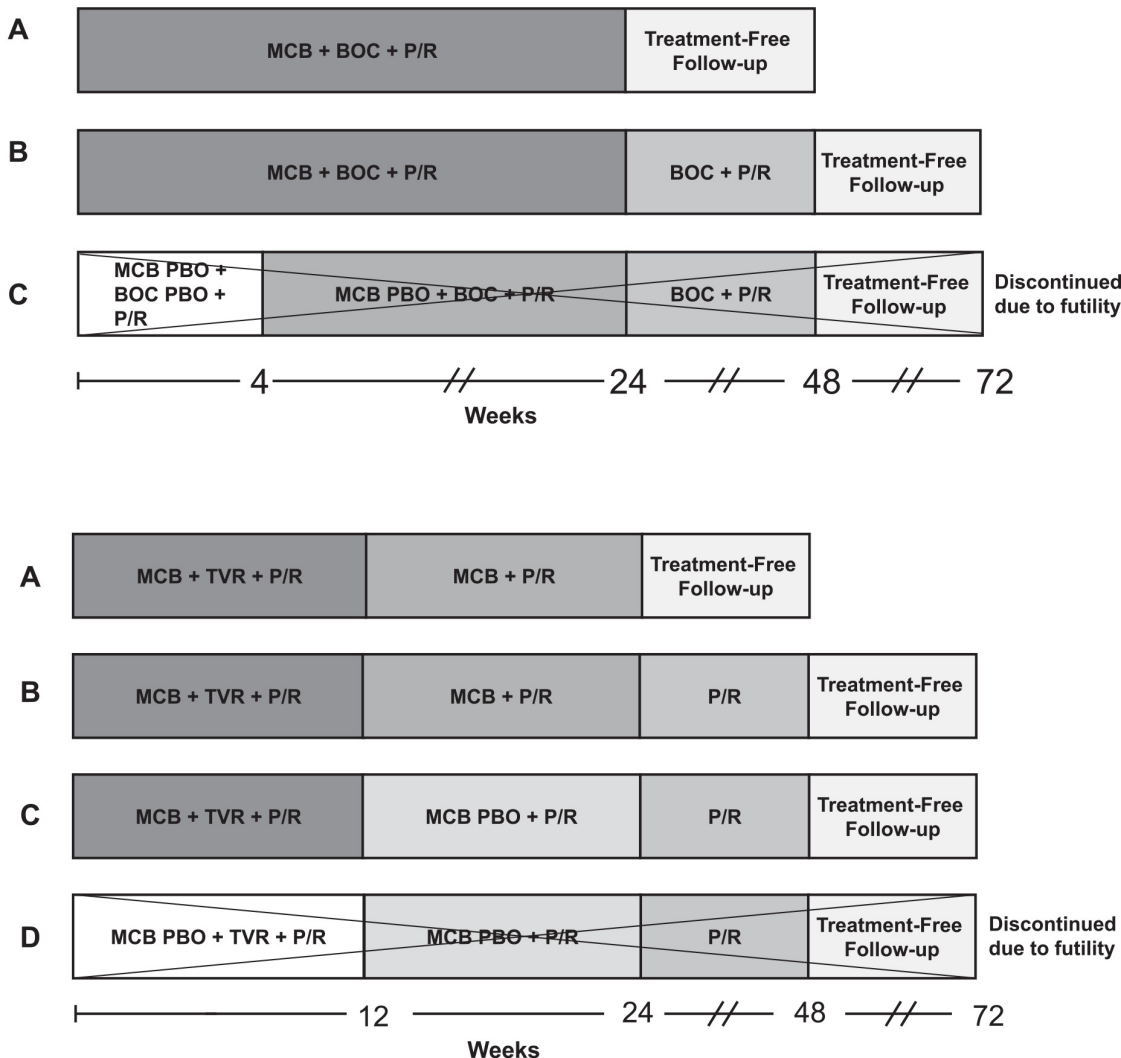
Study designs of DYNAMO 1 (a) and DYNAMO 2 (b). BOC, boceprevir 800 mg TID (at recommended intervals of 7–9 hours); MCB, mericitabine 1000 mg BID; P/R, peginterferon alfa-2a 180 μg once/week + ribavirin 1000 mg/day (<75 kg) or 1200 mg/day (≥75 kg); PBO, placebo; TVR, telaprevir 750 mg TID (at recommended intervals of 7–9 hours). Control Arms C in DYNAMO 1 and D in DYNAMO 2 were closed due to futility while the studies were ongoing (27 July 2012 in DYNAMO 1 and 10 July 2012 in DYNAMO 2), after which patients in these groups were given the option to receive mericitabine for a maximum duration of 24 weeks. MCB was added to the regimen for 5 patients in Arm C in DYNAMO 1 and to the regimen for 9 patients in Arm D in DYNAMO 2 at various time points and for various durations.

Patients in DYNAMO 1 were randomized (2:2:1) into one of three treatment groups (Fig 3A). Patients in Arm A received 24 weeks of treatment with MCB, BOC, and peginterferon alfa-2a/ribavirin (total duration: 24 weeks). The same therapy was administered in Arm B and followed by 24 weeks of BOC plus peginterferon alfa-2a/ribavirin (total duration: 48 weeks). Patients randomized to Arm C received 4 weeks of MCB placebo, BOC placebo, and peginterferon alfa-2a/ribavirin, followed by 20 weeks of MCB placebo, BOC, and peginterferon alfa-2a/ribavirin, followed by 24 weeks of BOC plus peginterferon alfa-2a/ribavirin, for a total treatment duration of 48 weeks.

Patients in DYNAMO 2 were randomized (2:2:2:1) between four treatment groups (Fig 3B). Patients in Arm A received 12 weeks of treatment with MCB, TVR, and peginterferon alfa-2a/ribavirin, followed by 12 weeks of treatment with MCB plus peginterferon alfa-2a/ribavirin (total duration: 24 weeks). Patients in Arm B received the same treatment as Arm A, followed by 24 weeks of peginterferon alfa-2a/ribavirin (total duration: 48 weeks). Those randomized to Arm C received 12 weeks of treatment with MCB, TVR, and peginterferon alfa-2a/ribavirin, followed by 12 weeks of treatment with MCB placebo plus peginterferon alfa-2a/ribavirin, and 24 weeks of peginterferon alfa-2a/ribavirin, for a total treatment duration of 48 weeks. Finally, patients in Arm D received the same treatment as in Arm C, except that MCB placebo replaced MCB during the first 12 weeks of treatment (total duration: 48 weeks).

MCB was administered at a dose of 1000 mg BID, BOC at 800 mg TID, TVR at 750 mg TID and peginterferon alfa-2a at 180 μg/week Ribavirin was given at initial doses of 1000 mg/day (body weight: <75 kg) or 1200 mg/day (body weight: ≥75 kg) and the daily dose was reduced in a step-wise manner in 200 mg (1 tablet) decrements in the event of adverse events or laboratory abnormalities.

Patients were recruited by the investigators. Randomization was centralized and stratified by HCV genotype (1a or 1b) and interleukin 28B (*IL28B*) genetic polymorphism (CC, CT, or TT). The computer-generated randomization list was maintained by the sponsor and was not accessible to staff at the study centers or study monitors. Randomization assignments were communicated to study centers by an interactive voice response system.

Study staff, monitors, and patients were blinded to MCB. Double-blinding was achieved by using matched placebo tablets.

While the studies were ongoing, emerging data from trials of novel DAA-based regimens prompted the decision to suspend placebo-controlled evaluations of MCB, and the control arms of DYNAMO 1 (Arm C) and DYNAMO 2 (Arm D) were closed. Patients previously randomized into these arms could receive MCB in addition to the assigned three-drug regimen at the investigator’s discretion.

### Assessments and outcomes

HCV RNA was extracted with the Roche High Pure PCR Product Purification system and quantified with the Roche COBAS^®^ TaqMan^®^ v2.0 assay according to the manufacturer’s recommendations (lower limit of quantification: 25 IU/mL, limit of detection 20 IU/mL; Roche Diagnostics, Indianapolis, IN). Resistance monitoring was conducted on samples from all patients at baseline and on post-baseline samples for patients with breakthrough (defined as either a sustained ≥1 log_10_ IU/mL increase in HCV RNA on ≥2 consecutive measurements while on treatment compared with the nadir [where the nadir was a ≥1 log_10_ IU/mL decrease from baseline after 2 weeks of treatment], or a confirmed quantifiable HCV RNA level [≥25 IU/mL on ≥2 consecutive measurements] after a confirmed unquantifiable HCV RNA level [<25 IU/mL on ≥2 consecutive measurements]) or relapse (defined as quantifiable HCV RNA [≥25 IU/mL on ≥2 consecutive measurements] after all treatment has been stopped in patients who had responded to treatment). Population sequencing of the coding regions of nonstructural protein 3/nonstructural protein 4A (NS3/4A) and nonstructural protein 5B (NS5B) was performed using standard sequencing methods. Safety assessments included monitoring of adverse events, laboratory parameters and vital signs.

### Statistics

Efficacy analyses were conducted according to the intent-to-treat principle among all randomized patients who received at least one study drug dose. Safety analyses included all randomized patients who received at least one study drug dose and who had at least one post-baseline safety assessment.

The primary efficacy outcome of both studies was SVR12, defined as HCV RNA <25 IU/mL 12 weeks after the actual end of treatment. SVR rates were summarized using descriptive statistics, and confidence intervals (CIs) were constructed using the Wilson score method without continuity correction. No formal hypothesis testing was planned. Pre-planned subgroup analyses were performed according to HCV genotype 1a or 1b infection and presence or absence of bridging fibrosis or cirrhosis. Relapse rate was calculated as the proportion of patients with quantifiable HCV RNA during follow-up among those with a confirmed end-of-treatment virological response and at least one post-treatment HCV RNA assessment. For the closed control arms of both studies, only baseline characteristics and safety outcomes are reported due to the wide variability in treatment received.

In DYNAMO 1, a sample size of 30 patients per arm was planned to provide a 90% CI of ±15% around an expected SVR12 rate of 45–55%. In DYNAMO 2, a sample size of 40 patients per arm was planned to provide a 90% CI of ±13% around the same expected SVR12 rate. The planned sample sizes were amended to 60 and 120 patients in DYNAMO 1 and 2, respectively, following closure of the control arms.

## Results

### Patient disposition

A total of 58 patients were randomized to Arms A (n = 25), B (n = 20), and C (n = 13) of DYNAMO 1 (Fig 1). Of the 57 patients who began treatment, 21 (84%), 16 (80%) and 6 (46%) completed treatment in Arms A, B, and C, respectively. Follow-up was completed by 24 patients (96%) in Arm A, 18 (90%) in Arm B, and 7 (54%) in Arm C. At the investigator’s discretion, MCB was added to treatment for five of the 12 patients who received treatment in Arm C.

Eighty patients were randomized between Arms A (n = 21), B (n = 24), C (n = 24), and D (n = 11) of DYNAMO 2 (Fig 2). Of the 79 who commenced treatment, 21 (100%), 17 (71%), 15 (63%) and 9 (82%) patients completed treatment in Arms A, B, C, and D, respectively. Twenty-four weeks of follow-up were completed by 17 patients (81%) in Arm A, 20 (83%) in Arm B, 23 (96%) in Arm C and 11 (100%) in Arm D. Nine of the 11 patients in Arm D received MCB at the investigator’s discretion.

### Baseline characteristics

Baseline characteristics of patients enrolled in DYNAMO 1 and DYNAMO 2 are presented in Table 1. Patients were predominantly male and a majority had HCV genotype 1a infection, host *IL28B* non-CC genotype and baseline HCV RNA level ≥800,000 IU/mL. The prevalence of bridging fibrosis or cirrhosis was 53.4% in DYNAMO 1 and 55% in DYNAMO 2. Within each study, baseline demographic and disease characteristics were balanced between the treatment arms.

**Table 1:** Baseline characteristics (all randomized patients). BOC, boceprevir; MCB, mericitabine; P/R, peginterferon alfa-2a/ribavirin; TVR, telaprevir

	DYNAMO 1			DYNAMO 2			
	Arm A	Arm B	Arm C	Arm A	Arm B	Arm C	Arm D
	MCB + BOC + P/R × 24 weeks	MCB + BOC + P/R × 24 weeks, then BOC + P/R × 24 weeks	P/R lead-in × 4 weeks, then BOC + P/R × 44 weeks*	MCB + TVR + P/R × 12 weeks, then MCB + P/R × 12 weeks	MCB + TVR + P/R × 12 weeks, then MCB + P/R ×12 weeks, then P/R × 24 weeks	MCB + TVR + P/R × 12 weeks, then P/R × 36 weeks	TVR + P/R × 12 weeks, then P/R × 36 weeks*
	n = 25	n = 20	n = 13	n = 21	n = 24	n = 24	n = 11
**Male, n (%)**	17 (68.0)	13 (65.0)	8 (61.5)	15 (71.4)	17 (70.8)	17 (70.8)	9 (81.8)
**Mean (SD) age, years**	53.8 (8.0)	53.3 (10.1)	56.0 (8.1)	51.9 (10.1)	54.0 (7.6)	53.5 (7.7)	52.8 (6.7)
**Race, n (%)**							
White	23 (92.0)	16 (80.0)	13 (100)	14 (66.7)	20 (83.3)	20 (83.3)	11 (100)
Black	2 (8.0)	4 (20.0)	0	5 (23.8)	4 (16.7)	2 (8.3)	0
Other	0	0	0	2 (9.6)	0	2 (8.3)	0
**Mean (SD) BMI, kg/m**^**2**^	26.3 (3.7)	28.1 (2.6)	27.7 (3.4)	27.3 (4.5)	26.6 (4.9)	27.7 (3.9)	25.8 (2.4)
**HCV genotype, n (%)**							
1a	13 (52.0)	12 (60.0)	8 (61.5)	14 (66.7)	16 (66.7)	15 (62.5)	6 (54.5)
1b	12 (48.0)	8 (40.0)	5 (38.5)	7 (33.3)	8 (33.3)	9 (37.5)	5 (45.5)
**Host *IL28B* genotype, n (%)**							
CC	2 (8.0)	1 (5.0)	2 (15.4)	0	3 (12.5)	0	0
Non-CC	23 (92.0)	19 (95.0)	11 (84.6)	21 (100)	21 (87.5)	24 (100)	11 (100)
**Bridging fibrosis/cirrhosis, n (%)**	11 (44.0)	12 (60.0)	8 (61.5)	10 (47.6)	14 (58.3)	13 (54.2)	7 (63.6)
**Mean (SD) serum ALT, IU/L**	128.9 (90.8	129.4 (71.0)	149.3 (151.4)	118.4 (62.9)	118.4 (60.9)	128.2 (79.3)	154.5 (254.0)
**Mean (SD) serum AST, IU/L**	87.1 (57.3)	93.2 (43.3)	102.0 (84.7)	77.5 (33.3)	83.5 (48.9)	98.1 (58.3)	87.5 (104.4)
**Mean (SD) serum albumin, g/L**	40.8 (3.5)	40.7 (4.2)	38.6 (3.4)	40.7 (3.7)	39.9 (4.0)	40.4 (2.9)	40.0 (2.7)
**Mean (SD) total bilirubin, μmol/L**	9.7 (4.1)	11.9 (5.4)	11.0 (5.1)	8.2 (2.1)	8.7 (2.9)	9.9 (3.4)	10.0 (5.1)
**Mean (SD) serum creatinine, μmol/L**	74.0 (10.7)	77.3 (20.5)	68.0 (11.3)	75.6 (15.4)	73.3 (15.4)	76.1 (9.9)	71.8 (9.6)
**Mean (SD) blood glucose, mmol/L**	5.9 (2.6)	6.1 (1.9)	6.1 (1.1)	6.3 (2.1)	6.2 (1.8)	5.9 (0.9)	5.8 (1.3)
Mean (SD) alpha-fetoprotein, μg/L**	24.8 (32.2) n = 9	37.4 (23.9) n = 12	18.2 (23.1) n = 4	10.9 (8.0) n = 11	8.7 (5.0) n = 11	23.3 (24.0) n = 15	27.3 (41.3) n = 5
**Mean (SD) erythrocytes, x 10**^**12**^**/L**	4.9 (0.4)	4.8 (0.3)	4.9 (0.3)	5.0 (0.4)	5.0 (0.4)	5.0 (0.3)	4.9 (0.4)
**Mean (SD) leukocytes, x 10**^**9**^**/L**	5.9 (2.3)	5.2 (1.2)	7.2 (4.4)	6.0 (1.7)	5.8 (2.2)	6.4 (1.8)	5.9 (2.1)
**Mean (SD) platelets, x 10**^**9**^**/L**	168.4 (64.7)	174.3 (72.7)	185.4 (72.8)	199.7 (70.3)	164.5 (41.6)	190.9 (62.3)	193.9 (55.6)
**Mean (SD) neutrophils, x 10**^**9**^**/L**	3.3 (1.6)	2.8 (1.1)	4.9 (4.4)	3.4 (1.6)	3.3 (1.9)	3.6 (1.2)	3.0 (1.0)
**Mean (SD), lymphocytes, x 10**^**9**^**/L**	2.0 (0.9)	1.9 (0.5)	1.8 (0.7)	2.1 (0.6)	1.9 (0.5)	2.1 (0.7)	2.2 (1.0)
**Median HCV RNA, log**^**10**^ **IU/mL (range)**	6.6 (5.3, 7.3)	6.7 (5.7, 7.1)	6.7 (5.8, 7.3)	6.9 (6.0, 7.4)	6.9 (5.5, 7.6)	6.8 (6.0, 7.3)	6.5 (5.9, 6.9)
**HCV RNA ≥800,000 IU/mL, n (%)**	23 (92.0)	19 (95.0)	12 (92.3)	21 (100)	21 (87.5)	24 (100)	11 (100)

* MCB could be added to treatment at the investigator’s discretion

** Not collected in all patients.

### Efficacy

**Fig 4:**
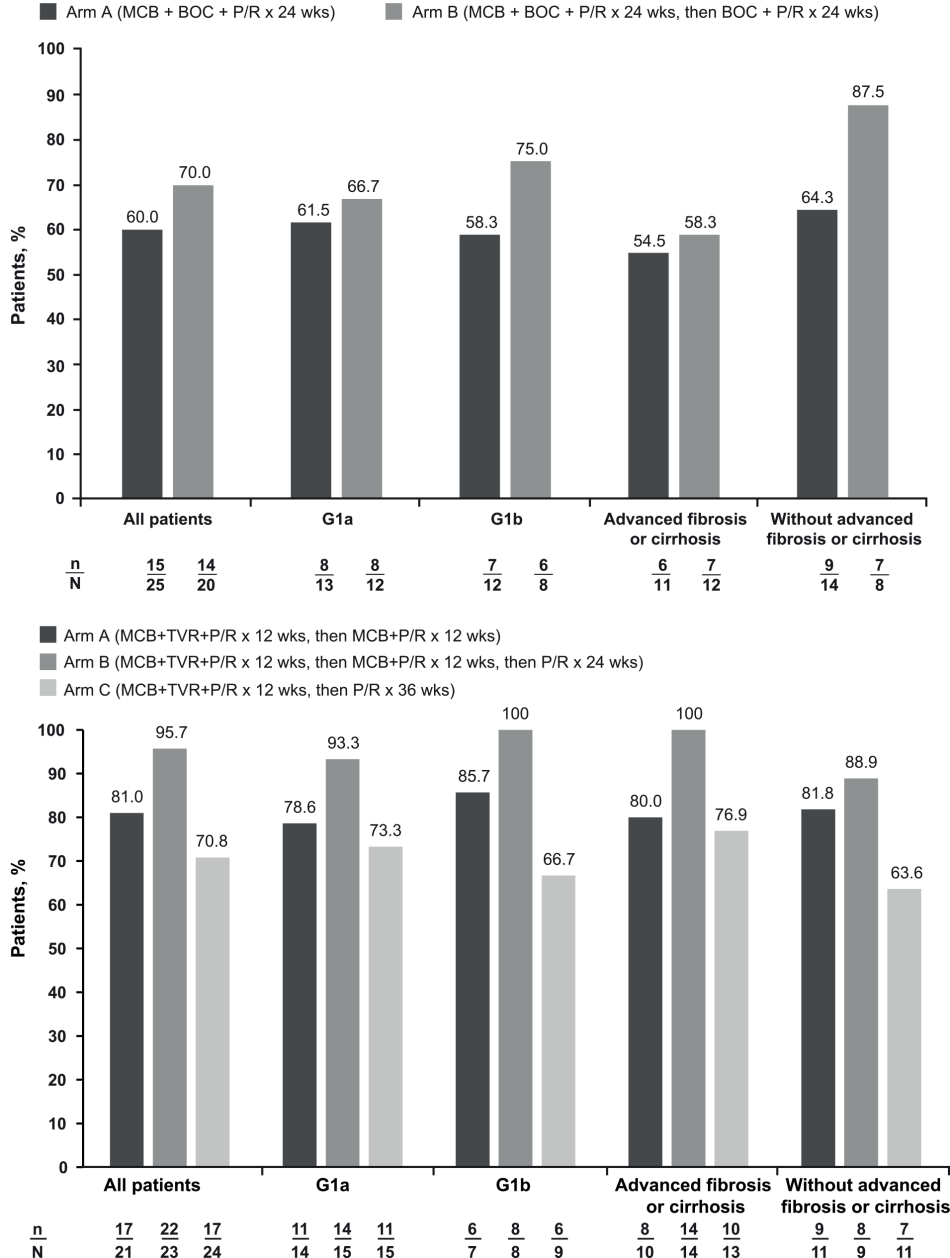
SVR12 rates by treatment arm in the overall populations and by HCV genotype and presence/absence of bridging fibrosis or cirrhosis in DYNAMO 1 (a) and DYNAMO 2 (b). BOC, boceprevir; MCB, mericitabine; P/R, peginterferon alfa-2a + ribavirin; TVR, telaprevir.

In DYNAMO 1, the rate of SVR12 was consistently greater in Arm B than in Arm A across the overall population and predefined subgroups, with the highest SVR12 rates observed in noncirrhotic patients. The primary endpoint of SVR12 was attained by 60.0% (95% CI: 40.7–76.6%) of patients in Arm A and 70.0% (95% CI: 48.1–85.5%) of patients in Arm B (Fig 4A, Table 2). Rates of SVR12 appeared similar between patients with HCV genotype 1a or 1b infection in Arm A (61.5% and 58.3%) and Arm B (66.7% and 75.0%). Higher rates of SVR12 were observed in noncirrhotic patients than in those with bridging fibrosis/cirrhosis in Arm A (64.3% and 54.5%) and Arm B (87.5% and 58.3%). SVR12 rates were identical to SVR24 rates in all subgroups (Table 2). At the end of 12-weeks follow-up, relapse occurred in 8/23 patients (34.8%) in Arm A and 2/16 patients (12.5%) in Arm B.

**Table 2:** Virological response at end of treatment and at week 4, 12 and 24 of follow-up (all treated patients) and relapse at week 4, 12 and 24 of follow-up. BOC, boceprevir; CI, 95% confidence interval; MCB, mericitabine; P/R, peginterferon alfa-2a/ribavirin; TVR, telaprevir

	DYNAMO 1			DYNAMO 2			
	Arm A	Arm B	Arm C	Arm A	Arm B	Arm C	Arm D
	MCB + BOC + P/R × 24 weeks	MCB + BOC + P/R × 24 weeks, then BOC + P/R × 24 weeks	P/R lead-in × 4 weeks, then BOC + P/R × 44 weeks*	MCB + TVR + P/R × 12 weeks, then MCB + P/R × 12 weeks	MCB + TVR + P/R × 12 weeks, then MCB + P/R ×12 weeks, then P/R × 24 weeks	MCB + TVR + P/R × 12 weeks, then P/R × 36 weeks	TVR + P/R × 12 weeks, then P/R × 36 weeks*
	n = 25	n = 20	n = 12	n = 21	n = 23	n = 24	n = 11
**Response at actual end of treatment, n (%; CI)**	23 (92.0; 75.0, 97.8)	17 (85.0; 64.0, 94.8)	8 (66.7; 39.1, 86.2)	21 (100; 84.5, 100)	22 (95.7; 79.0, 99.2)	17 (70.8; 50.8, 85.1)	10 (90.9; 62.3, 98.4)
**SVR4, n (%; CI)**	15 (60.0; 40.7, 76.6)	15 (75.0; 53.1, 88.8)	4 (33.3; 13.8, 60.9)	18 (85.7; 65.4, 95.0)	22 (95.7; 79.0, 99.2)	17 (70.8; 50.8, 85.1)	10 (90.9; 62.3, 98.4)
**SVR12, n (%; CI)**	15 (60.0; 40.7, 76.6)	14 (70.0; 48.1, 85.5)	4 (33.3; 13.8, 60.9)	17 (81.0; 60.0, 92.3)	22 (95.7; 79.0, 99.2)	17 (70.8; 50.8, 85.1)	10 (90.9; 62.3, 98.4)
**SVR24, n (%; CI)**	15 (60.0; 40.7, 76.6)	14 (70.0; 48.1, 85.5)	4 (33.3; 13.8, 60.9)	16 (76.2; 54.9, 89.4)	22 (95.7; 79.0, 99.2)	17 (70.8; 50.8, 85.1)	10 (90.9; 62.3, 98.4)
**Relapse at follow-up Week 4, n/n (%; CI)**	8/22 (36.4; 19.7, 57.0)	1/16 (6.3; 1.1, 28.3)	3/7 (42.9; 15.8, 75.0)	1/19 (5.3; 0.9, 24.6)	0/22 (0; 0, 14.9)	0/15 (0; 0, 20.4)	0/10 (0; 0, 27.8)
**Relapse at follow-up Week 12, n/n (%; CI)**	8/23 (34.8; 18.8, 55.1)	2/16 (12.5; 3.5, 36.0)	3/7 (42.9; 15.8, 75.0)	2/19 (10.5; 2.9, 31.4)	0/22 (0; 0, 14.9)	0/17 (0; 0, 18.4)	0/10 (0; 0, 27.8)
**Relapse at follow-up Week 24, n/n (%; CI)**	8/23 (34.8; 18.8, 55.1)	2/16 (12.5; 3.5, 36.0)	3/7 (42.9; 15.8, 75.0)	3/19 (15.8; 5.5, 37.6)	0/22 (0; 0, 14.9)	0/17 (0; 0, 18.4)	0/10 (0; 0, 27.8)

* MCB could be added to treatment at the investigator’s discretion

In DYNAMO 2, the highest SVR12 rates were achieved in patients who received both the longest duration of treatment with MCB and the longest overall treatment duration (Arm B). SVR12 was attained by 81.0% (95% CI: 60.0–92.3%) of patients in Arm A, 95.7% (95% CI: 79.0–99.2%) of patients in Arm B, and 70.8% (95% CI: 50.8–85.1%) of patients in Arm C (Fig 4B, Table 2). Rates of SVR12 in patients with HCV genotype 1a and 1b infection were 78.6% and 85.7% in Arm A, 93.3% and 100% in Arm B, and 73.3% and 66.7% in Arm C. Among patients with bridging fibrosis or cirrhosis, SVR12 was achieved by 80.0% of patients in Arm A, 100.0% in Arm B, and 76.9% in Arm C; corresponding SVR12 rates for noncirrhotic patients were 81.8%, 88.9%, and 63.6%, respectively. Twelve weeks after the end of treatment, 3/19 patients (15.8%) in Arm A had relapse, whereas no relapses were observed in the other arms (Table 2).

### Resistance

In DYNAMO 1, 10 patients in Arm A (two of whom experienced breakthrough [both G1a], and eight of whom experienced relapse [three G1a, five G1b]) and three patients in Arm B (one of whom experienced breakthrough [G1a] and two of whom experienced relapse [one G1a and one G1b]) were monitored for viral resistance.

BOC resistance mutations (in NS3) were identified in seven of these patients: T54S in two patients in Arm A who relapsed (one G1a and one G1b), V55A in one patient in Arm B who relapsed (G1b), R155K in one patient in Arm B who relapsed (G1a), V36M-T54S in one patient in Arm B who experienced breakthrough (G1a), T54S/T-V170A in one patient in Arm A who relapsed (G1b), A156S-M175L in one patient in Arm A who relapsed (G1b). No MCB resistance mutations were detected. Pre-existing BOC resistance mutations (M175L or T54S/V55I) were identified in baseline samples from two patients (both of whom achieved an SVR). No pre-existing MCB resistance mutations were detected.

In DYNAMO 2, two patients in Arm A who experienced relapse (one G1a and one G1b), one patient in Arm B who experienced breakthrough while on P/R (G1a), and six patients in Arm C who experienced breakthrough while on P/R (four G1a and two G1b) were monitored for viral resistance. TVR resistance mutations (in NS3) were identified in seven of these patients: R155K in two patients in Arm C who experienced breakthrough (G1a), V36M in one patient in Arm A who relapsed (G1b), T54S in one patient in Arm C who experienced breakthrough (G1b), V36A/V-T54A/T in one patient in Arm C who experienced breakthrough (G1b), V36M-R155K in one patient in Arm C who experienced breakthrough (G1a), and V36M-R155R/T in one patient in Arm A who relapsed (G1a). No MCB resistance mutations were detected. Pre-existing TVR resistance mutations (V36L or T54S) were identified in baseline samples from two patients (both of whom achieved an SVR). No pre-existing MCB mutations were detected.

### Safety

In DYNAMO 1, all patients experienced at least one adverse event, the majority of which were of mild or moderate intensity. The most common adverse events included anaemia, dysgeusia and nausea (Table 3). The incidence of these events was generally similar in each arm, with the exception of dysgeusia, which was not reported for any patient in Arm C. One patient in Arm A, four in Arm B, and two in Arm C withdrew due to adverse events. Seven serious adverse events (SAEs) were reported in six patients, of which three were considered related to study treatment: thrombocytopenia in one patient in Arm B and two incidences of anemia in the same patient in Arm C. The SAE of thrombocytopenia related to treatment occurred in a 48-year-old woman whose baseline platelet count was 125 x 10^9^/L. Her lowest platelet count (19 x 10^9^/L) occurred on study day 133. She was hospitalized on four occasions for platelet transfusions. All treatment with study medications was discontinued on study day 168. The two incidences of anemia considered to be related to ribavirin occurred in a 71-year-old woman whose baseline hemoglobin concentration (135 g/dL) and creatinine clearance (85.8 mL/min) were both in the normal range. On study day 43, her hemoglobin concentration was 74 g/L, at which time she was hospitalized and received treatment with oral iron supplements, folic acid, and two units of packed red blood cells. Treatment with ribavirin was withheld for three days. The second episode of anemia occurred on study day 73 (hemoglobin concentration not reported), at which time all treatment with study drugs was discontinued. On study day 77, she received a second transfusion of two units of packed red blood cells. No deaths were reported during the study. High rates of grade ≥3 reductions in hemoglobin and neutrophils were reported for all three arms of the study; grade 3 platelet decreases were also common (Table 3).

**Table 3:** Adverse events and laboratory abnormalities. ALT, alanine transaminase; BOC, boceprevir; MCB, mericitabine; P/R, peginterferon alfa-2a/ribavirin; TVR, telaprevir; ULN, upper limit of normal

Patients, n (%)	DYNAMO 1			DYNAMO 2			
	Arm A	Arm B	Arm C	Arm A	Arm B	Arm C	Arm D
	MCB + BOC + P/R × 24 weeks	MCB + BOC + P/R × 24 weeks, then BOC + P/R × 24 weeks	P/R lead-in × 4 weeks, then BOC + P/R × 44 weeks*	MCB + TVR + P/R × 12 weeks, then MCB + P/R × 12 weeks	MCB + TVR + P/R × 12 weeks, then MCB + P/R ×12 weeks, then P/R × 24 weeks	MCB + TVR + P/R × 12 weeks, then P/R × 36 weeks	TVR + P/R × 12 weeks, then P/R × 36 weeks*
	n = 25	n = 20	n = 12	n = 21	n = 23	n = 24	n = 11
**Patients with ≥1 AE**	25 (100)	20 (100)	12 (100)	20 (95)	23 (100)	24 (100)	11 (100)
**Patients with ≥1 serious AE**	1 (4)	3 (15)	2 (17)	4 (19)	6 (26)	3 (13)	0
**Patients with ≥1 serious treatment-related AE**	0	1 (5)	1 (8)	3 (14)	3 (13)	1 (4)	0
**Incidence of individual AEs**^**†**^							
Anemia	10 (40)	13 (65)	6 (50)	7 (33)	11 (48)	11 (46)	6 (55)
Dysgeusia	11 (44)	11 (55)	0	5 (24)	2 (9)	2 (8)	1 (9)
Nausea	9 (36)	8 (40)	6 (50)	9 (43)	8 (35)	7 (29)	3 (27)
Headache	7 (28)	7 (35)	6 (50)	9 (43)	9 (39)	9 (38)	5 (46)
Fatigue	8 (32)	8 (40)	4 (33)	12 (57)	14 (61)	12 (50)	4 (36)
Pruritus	8 (32)	7 (35)	3 (25)	7 (33)	12 (52)	9 (38)	2 (18)
Asthenia	7 (28)	6 (30)	4 (33)	4 (19)	5 (22)	6 (25)	4 (36)
Chills	3 (12)	6 (30)	4 (33)	4 (19)	3 (13)	2 (8)	2 (18)
Cough	6 (24)	5 (25)	4 (33)	2 (10)	7 (30)	10 (42)	3 (27)
Pyrexia	3 (12)	5 (25)	4 (33)	3 (14)	4 (17)	4 (17)	4 (36)
Rash	5 (20)	3 (15)	1 (8)	6 (29)	7 (30)	14 (58)	2 (18)
Decreased appetite	4 (16)	5 (25)	2 (17)	5 (24)	5 (22)	3 (13)	4 (36)
Diarrhea	6 (24)	3 (15)	3 (25)	5 (24)	8 (35)	4 (17)	2 (18)
Neutropenia	3 (12)	6 (30)	3 (25)	4 (19)	17 (30)	3 (13)	1 (9)
Dyspnea exertional	5 (20)	6 (30)	0	3 (14)	7 (30)	4 (17)	2 (18)
**Grade 3/4 laboratory abnormalities**							
ALT ≥5.1–10.0 × ULN	3 (12)	0	2 (17)	0	0	1 (4)	1 (9)
Hemoglobin 7.0–8.9 g/dL or any decrease ≥4.5 g/dL	9 (36)	11 (55)	5 (42)	10 (48)	15 (65)	15 (63)	10 (91)
Hemoglobin <7.0 g/dL	0	0	0	0	1 (4)	0	0
Neutrophils 0.5–0.749 × 10^9^ cells/L	7 (28)	8 (40)	3 (25)	5 (24)	4 (17)	1 (4)	4 (36)
Neutrophils <0.5 × 10^9^ cells/L	2 (8)	3 (15)	2 (17)	1 (5)	3 (13)	1 (4)	0
Lymphocytes 0.35–0.499 × 10^9^ cells/L	3 (12)	2 (10)	3 (25)	0	5 (22)	2 (8)	2 (18)
Lymphocytes <0.35 × 10^9^ cells/L	0	1 (5)	0	0	3 (13)	1 (4)	0
Platelets 25–<50 × 10^9^/L	2 (8)	5 (25)	3 (25)	0	2 (9)	1 (4)	0

* MCB could be added to treatment at the investigator’s discretion

^†^ AEs reported in >30% of patients in at least one arm of either study

In DYNAMO 2, the majority of patients experienced at least one AE, these being generally of mild or moderate intensity. The most common adverse events occurred with similar frequencies in each arm and included fatigue, rash, and anemia (Table 2). Two patients in Arm A, four in Arm B, four in Arm C, and two in Arm D discontinued study medication due to adverse events. Both of the safety-related discontinuations in Arm A and one in Arm D were due to dermatological events. A total of 15 SAEs were reported in 13 patients. SAEs considered related to study treatment included: abdominal wall abscess, rash, and anemia (in the same patient), and rash in Arm A; viral gastroenteritis, neutropenia, and anemia in Arm B; and two occurrences of anemia in one patient in Arm C. No patients with SAEs considered to be related to treatment required hospitalization. All study treatment was discontinued in the patient in Arm B who experienced anemia, although no treatment was given for the SAE and the event was considered to have resolved by the time of reporting. Treatment with PegIFN alfa-2a/ribavirin was interrupted in the patient in Arm B who experienced neutropenia, and the dose of ribavirin was reduced in the patients in Arms A and C who experienced anemia as SAEs. No deaths occurred during the study. The most common grade ≥3 laboratory abnormalities were reductions in hemoglobin and neutrophil count, which affected a substantial proportion of patients in each study arm (Table 2).

## Discussion

The DYNAMO 1 and DYNAMO 2 studies were conducted to evaluate whether the addition of MCB could improve on the generally low SVR rates achieved with BOC- or TVR-based triple therapy in patients with chronic HCV genotype 1 infection and prior null response to peginterferon alfa-2a/ribavirin. In addition to the requirement for prior null response, both studies recruited a high proportion of patients with bridging fibrosis or cirrhosis, and so included populations that have typically been regarded as difficult-to-treat with first-generation protease inhibitor-based combinations. Although caution must be applied in drawing cross-trial comparisons, SVR12 rates observed in the present studies (70% in Arm B of DYNAMO 1 and 96% in Arm B of DYNAMO 2) are considerably higher than those previously reported for the approved first- and second-generation protease inhibitor-based triple regimens in patients with prior null response (31–46%) [17, 18, 26]. These results suggest that SVR12 rates with BOC- or TVR-based triple regimens can indeed be increased in this patient population by incorporation of the second DAA MCB, which has been shown to provide a high barrier to the development of resistance [18–21, 24].

In both DYNAMO 1 and DYNAMO 2, the highest SVR12 rates and lowest incidences of relapse were attained when 24 weeks of treatment with an MCB-containing, four-drug regimen was followed by 24 weeks of peginterferon alfa-2a/ribavirin (administered with BOC in DYNAMO 1). Consistent with *in vitro* data [27, 28], we hypothesize that depletion of the immuno-inhibitory NS3/4A serine protease with DAA-based therapy may restore interferon responsiveness and so explain the higher SVR rates achieved with extended administration of peginterferon alfa/ribavirin in patients previously nonresponsive to this combination [29]. The inclusion of MCB seemed to diminish HCV subtype-dependent differences in SVR12 rates that have typically been observed in clinical trials of first-generation PI-containing triple therapy regimens [10, 11, 30]. Thus, SVR12 rates were similar in G1b and G1a patients enrolled in both trials. In contrast, SVR12 rates have generally been higher by 5–29% in G1b than G1a patients in previous trials of protease inhibitor-based triple therapy [10, 11, 26, 30]. As previously observed with protease inhibitor-based three-drug regimens [9–12, 26], SVR12 rates were generally higher among noncirrhotic patients compared with patients with bridging fibrosis/cirrhosis.

The higher SVR12 rates in DYNAMO 1 and DYNAMO 2 relative to prior studies of protease inhibitor-based triple therapy combinations most likely reflect a reduction in the incidence of protease inhibitor resistance with the incorporation of MCB into therapy. In particular, the rate of relapse across Arms A–C of DYNAMO 2 12 weeks after end of treatment (3%) appeared substantially lower than the 26% relapse rate at week 72 reported in the Phase 3 study of TVR plus peginterferon alfa/ribavirin among prior null responders [12]. Viral breakthrough on BOC- or TVR-containing three-drug regimens is associated with the selection of protease inhibitor-resistant virus, which commonly become established as the predominant population at the time of treatment failure [31–34]. As expected, BOC or TVR resistance mutations were found by population sequencing in the majority of patients who experienced treatment failure. In contrast to first-generation protease inhibitors, MCB has a high barrier to resistance [18–21], as supported by the absence of MCB-resistant variants in the present studies and in previous trials of MCB in combination with peginterferon alfa-2a/ribavirin [18, 22–24].

The adverse event profiles of the MCB-containing four-drug regimens, including the rates of serious adverse events and discontinuations due to safety reasons, were similar to reports for BOC or TVR in combination with peginterferon/ribavirin, suggesting that MCB did not add to the safety burden [9–13, 30]. This observation is in line with prior studies in which MCB was well tolerated as a component of triple or quadruple therapy regimens including peginterferon alfa/ribavirin and/or danoprevir/r [18, 22, 23]. Consistent with the more favorable tolerability profile of danoprevir/r compared with first-generation protease inhibitors [35], serious adverse events and withdrawals due to adverse events were more common in the present studies than reported for MCB in combination with danoprevir/r and peginterferon alfa/ribavirin [18].

The early termination of the control arms limits the strength of the conclusions that can be drawn from the DYNAMO studies. However, the SVR12 rates associated with the addition of MCB to BOC- and TVR-based regimens in these studies are remarkably better than the historically poor outcomes achieved with BOC- and TVR-based triple therapy in null responders, especially in patients with advanced liver disease [12, 13, 16]. The ability to detect rare variants was limited because resistance monitoring was based on population sequencing; however, this limitation is of minimal clinical significance because even population sequencing is capable of detecting variants associated with on-treatment failure and relapse.

The treatment of chronic HCV infection is currently undergoing rapid change. At the time the DYNAMO 1 and DYNAMO 2 studies were designed, BOC- and TVR-containing triple regimens represented the standard of care for HCV genotype 1 infection [36]; however, these regimens have now been superseded by treatments based on new DAAs [3, 4]. BOC- and TVR-based therapies are therefore no longer recommended in US clinical practice guidelines [3]; in contrast, European clinical guidelines recommend these agents in settings where access to newer agents is limited [4]. Although a very high SVR12 rate (96%) was attained in Arm B of DYNAMO 2, a full 48 weeks of treatment with peginterferon-containing therapy was required to achieve this impressive result. Mericitabine has also been studied in combination with peginterferon alfa/ribavirin in treatment-naïve patients [22, 23]. While a 24-week response-guided regimen with MCB was well tolerated, did not select resistant variants and increased SVR compared with peginterferon alfa/ribavirin [22], given the efficacy and safety profiles of the new interferon-free regimens, the development of MCB in combination with peginterferon alfa/ribavirin has been discontinued. Nonetheless, ensuring widespread access to the novel DAA-based regimens is now a major challenge for management of chronic HCV infection in clinical practice.

Collectively, the results of the DYNAMO 1 and DYNAMO 2 studies suggest that the addition of 24 weeks of treatment with a nucleoside analog inhibitor with intermediate antiviral potency to BOC- or TVR-based combinations improves SVR12 rates.

## Supporting Information

S1 File CONSORT checklist. (DOC)

S2 File NV27780F protocol. (PDF)

S3 File NV27779F protocol. (PDF)
