# Supplementary material for: Mericitabine and Either Boceprevir or Telaprevir in Combination with Peginterferon Alfa-2a plus Ribavirin for Patients with Chronic Hepatitis C Genotype 1 Infection and Prior Null Response: The Randomized DYNAMO 1 and DYNAMO 2 Studies
Source: PLoS One. 2016 Jan 11;11(1):e0145409. doi: 10.1371/journal.pone.0145409 (PMC4713467; doi:10.1371/journal.pone.0145409)
Supplement: S2 File — (PDF) [file pone.0145409.s002.pdf]

PROTOCOL

TITLE: **A PHASE II, RANDOMIZED, DOUBLE-BLIND, MULTICENTER, PARALLEL GROUP STUDY TO EVALUATE THE SUSTAINED VIROLOGIC RESPONSE OF THE HCV POLYMERASE INHIBITOR PRODRUG RO5024048 IN COMBINATION WITH BOCEPREVIR AND PEGASYS®/COPEGUS® IN PATIENTS WITH CHRONIC HEPATITIS C GENOTYPE 1 VIRUS INFECTION WHO WERE PRIOR NULL RESPONDERS TO TREATMENT WITH PEGYLATED INTERFERON/RIBAVIRIN**

PROTOCOL NUMBER: NV27780 **VERSION NUMBER:** *6*

EUDRACT NUMBER: 2011-002714-37 **IND NUMBER:** 73,760

TEST PRODUCT: RO5024048

MEDICAL MONITOR: Astrid Scalori, M.D.

SPONSOR: F. Hoffmann-La Roche Ltd

DATE FINAL: Version A: 1 July 2011

DATES AMENDED: Version B: 5 March 2012
Version C: 16 March 2012
Version D: 27 July 2012
Version E: *28 November 2012*
Version F: See Electronic stamp below

PROTOCOL AMENDMENT APPROVAL

Approver's Name	Title	Date and Time (UTC)
Thommes,James_A	Clinical Science Leader	15-Feb-2013 21:50:00

CONFIDENTIAL STATEMENT

The information contained in this document, especially any unpublished data, is the property of F. Hoffmann–La Roche Ltd (or under its control) and therefore is provided to you in confidence as an investigator, potential investigator, or consultant, for review by you, your staff, and an applicable Ethics Committee or Institutional Review Board. It is understood that this information will not be disclosed to others without written authorization from Roche except to the extent necessary to obtain informed consent from persons to whom the drug may be administered.

PROTOCOL AMENDMENT, VERSION *F*
RATIONALE

Protocol NV27780 has been amended because in the schedule of assessments the Week 42 visit during the treatment phase was accidentaly omitted. This omission is addressed in this protocol amendment.

During the design of the protocol it was determined, based on the data from the ongoing HCV studies, that after Week 24 of the treatment phase of the study a visit interval of 6 weeks would be most appropriate to monitor the continued safety and efficacy of treatment with Pegasus®/Copegus® and the experimental drug mericitabine (RO5024048). However, in the schedule of assessments, the Week 42 visit was accidentally omitted.

We are planning to introduce a visit at Week 42, repeating the assessments done at Week 30. After the approval of the amendment, the Week 42 visit will be implemented in the eCRF.

PROTOCOL AMENDMENT, VERSION *F*:
SUMMARY OF CHANGES

APPENDIX A: Schedule of Assessments

The Week 42 visit was added to the schedule of assessments.

SAMPLE INFORMED CONSENT FORM

The sample Informed Consent Form has been revised to reflect the changes to the protocol.

TABLE OF CONTENTS

PROTOCOL AMENDMENT ACCEPTANCE FORM 9

PROTOCOL SYNOPSIS 10

LIST OF ABBREVIATIONS AND DEFINITIONS OF TERMS............................ 16

1. BACKGROUND 19

1.1 Background on Chronic Hepatitis C...................................... 19

1.2 Background on Test Molecules 20

1.2.1 HCV Polymerase Inhibitor Prodrug (RO5024048)................. 20

1.2.2 HCV Protease Inhibitor (Boceprevir) 22

1.2.3 Pegasys (Peginterferon Alfa-2a)... 23

1.2.4 Copegus (Ribavirin)... 23

1.3 Study Rationale and Benefit–Risk Assessment..................... 23

2. OBJECTIVES.. 24

2.1 Primary Objective 24

2.2 Secondary Objectives.. 25

2.3 Exploratory Objective.. 25

3. STUDY DESIGN 25

3.1 Description of Study 25

3.2 End of Study... 26

3.3 Rationale for Study Design 26

3.3.1 Rationale for Test Product Dosage....................................... 28

3.3.2 Rationale for Patient Population 29

3.3.3 Rationale for Biomarker Assessments.................................. 29

3.4 Outcome Measures 29

3.4.1 Efficacy Outcome Measures.. 29

3.4.1.1 Primary Efficacy Outcome Measure 29

3.4.1.2 Secondary Efficacy Outcome Measures................................ 30

3.4.2 Safety Outcome Measures 30

3.4.3 Pharmacokinetic Outcome Measures.................................... 30

3.4.4 Exploratory Outcome Measure 30

4. MATERIALS AND METHODS 31

4.1 Patients 31
4.1.1 Inclusion Criteria 31
4.1.2 Exclusion Criteria 32
4.2 Method of Treatment Assignment and Blinding 35
4.3 Study Treatment 36
4.3.1 Formulation, Packaging, and Handling 36
4.3.2 Dosage, Administration, and Compliance 36
4.3.3 Investigational Medicinal Product Accountability 37
4.3.4 Posttrial Access 37
4.4 Concomitant Therapy 37
4.4.1 Permitted Therapy 37
4.4.2 Growth Factor Use 38
4.4.3 Prohibited Therapy 39
4.5 Study Assessments 39
4.5.1 Description of Study Assessments 39
4.5.1.1 Efficacy Assessments 39
4.5.1.2 Safety Assessments 39
4.5.1.3 Laboratory Assessments 39
4.5.1.4 Electrocardiograms 40
4.5.1.5 Pharmacokinetic Assessments 40
4.5.1.6 Pharmacodynamic Assessments 41
4.5.1.7 Exploratory Biomarker Assessments 41
4.5.1.8 Hepatitis C Virus Drug-Resistance Monitoring 41
4.5.1.9 Samples for Roche Clinical Repository 45
4.5.2 Timing of Study Assessments 47
4.5.2.1 Screening and Pretreatment Assessments 47
4.5.2.2 Assessments During Treatment 48
4.5.2.3 Assessments at Study Completion/Early Termination Visit 48
4.5.2.4 Follow-Up Assessments 48
4.6 Patient, Study, and Site Discontinuation 49
4.6.1 Patient Discontinuation 49
4.6.1.1 Discontinuation from Study Drug 49

4.6.1.2 Withdrawal from Study 50
4.6.2 Study and Site Discontinuation 50

5. ASSESSMENT OF SAFETY 51
5.1 Safety Plan 51
5.1.1 Management of Specific AEs 51
5.2 Safety Parameters and Definitions 52
5.2.1 Adverse Events 52
5.2.2 Serious Adverse Events (Immediately Reportable to Roche) 52
5.3 Methods and Timing for Capturing and Assessing Safety Parameters 53
5.3.1 Adverse Event Reporting Period 53
5.3.2 Eliciting AE Information 53
5.3.3 Assessment of Severity of AEs 54
5.3.4 Assessment of Causality of AEs 54
5.3.5 Procedures for Recording AEs 55
5.3.5.1 Diagnosis versus Signs and Symptoms 55
5.3.5.2 Adverse Events Occurring Secondary to Other Events 55
5.3.5.3 Persistent or Recurrent AEs 56
5.3.5.4 Abnormal Laboratory Values 56
5.3.5.5 Abnormal Vital Sign Values 57
5.3.5.6 Abnormal Liver Function Tests 57
5.3.5.7 Deaths 58
5.3.5.8 Pre-Existing Medical Conditions 58
5.3.5.9 Lack of Efficacy or Worsening of CHC 58
5.3.5.10 Hospitalization or Prolonged Hospitalization 59
5.3.5.11 Overdoses 59
5.4 Immediate Reporting Requirements from Investigator to Sponsor 59
5.4.1 Emergency Medical Contacts 60
5.4.2 Reporting Requirements for SAEs and Non-Serious AEs of Special Interest 60
5.4.3 Reporting Requirements for Pregnancies 61

5.4.3.1 Pregnancies in Female Patients 61
5.4.3.2 Pregnancies in Female Partners of Male Patients 61
5.4.3.3 Abortions 62
5.4.3.4 Congenital Anomalies/Birth Defects 62
5.5 Follow-Up of Patients after AEs 62
5.5.1 Investigator Follow Up 62
5.5.2 Sponsor Follow Up 62
5.6 POSTSTUDY AEs 62
5.7 Expedited Reporting to Health Authorities, Investigators, IRBs, and ECs 63

6. STATISTICAL CONSIDERATIONS AND ANALYSIS PLAN 63
6.1 Determination of Sample Size 63
6.2 Summaries of Conduct of Study 64
6.3 Summaries of Treatment Group Comparability 64
6.4 Efficacy Analyses 64
6.4.1 Primary Efficacy Endpoint 64
6.4.2 Secondary Efficacy Endpoints 64
6.5 Safety Analyses 64
6.6 Subgroup Analyses 65
6.7 Resistance Analyses 66
6.8 Pharmacokinetic Analyses 66
6.9 Exploratory Analyses 66
6.9.1 Biomarker Analyses 67
6.10 Interim Analyses 67

7. DATA COLLECTION AND MANAGEMENT 67
7.1 Data Quality Assurance 67
7.2 Electronic Case Report Forms 67
7.3 Source Data Documentation 68
7.4 Use of Computerized Systems 68
7.5 Retention of Records 69

8. ETHICAL CONSIDERATIONS 69
8.1 Compliance with Laws and Regulations 69

8.2 Informed Consent 69
8.3 Institutional Review Board or EC 70
8.4 Confidentiality 71
8.5 Financial Disclosure 71

9. STUDY DOCUMENTATION, MONITORING, AND ADMINISTRATION 71
9.1 Study Documentation 71
9.2 Site Inspections 71
9.3 Publication of Data and Protection of Trade Secrets 72
9.4 Protocol Amendments 72

10. REFERENCES 73

LIST OF TABLES

Table 1 Study Design 26
Table 2 AE Severity Grading Scale 54

LIST OF FIGURES

Figure 1 Exposure–Response Curve for RO4995855 (Ctrough): Study 7081-5101 28

LIST OF APPENDICES

Appendix A Schedule of Assessments 77
Appendix B Liver Biopsy Scores 81
Appendix C Creatinine Clearance Calculation 83
Appendix D Child–Pugh Classification of Severity of Liver Disease 84
Appendix E Package Inserts for Boceprevir, Pegasys, and Copegus 85
Appendix F Dose Modifications, Interruptions, and Delays 86
Appendix G Management of Specific Adverse Events and Laboratory Abnormalities 91
Appendix H Urinary Protein Monitoring Plan 94
Appendix I Division of Aids Table for Grading the Severity of Adult and Pediatric Adverse Events 96

PROTOCOL AMENDMENT ACCEPTANCE FORM

**TITLE:**	**A PHASE II, RANDOMIZED, DOUBLE-BLIND, MULTICENTER, PARALLEL GROUP STUDY TO EVALUATE THE SUSTAINED VIROLOGIC RESPONSE OF THE HCV POLYMERASE INHIBITOR PRODRUG RO5024048 IN COMBINATION WITH BOCEPREVIR AND PEGASYS®/COPEGUS® IN PATIENTS WITH CHRONIC HEPATITIS C GENOTYPE 1 VIRUS INFECTION WHO WERE PRIOR NULL RESPONDERS TO TREATMENT WITH PEGYLATED INTERFERON/RIBAVIRIN**
**PROTOCOL NUMBER:**	NV27780
**VERSION NUMBER:**	*6*
**EUDRACT NUMBER:**	2011-002714-37
**IND NUMBER:**	73,760
**TEST PRODUCT:**	RO5024048
**MEDICAL MONITOR:**	Astrid Sclalori, M.D.
**SPONSOR:**	F. Hoffmann–La Roche Ltd

I agree to conduct the study in accordance with the current protocol.

____

Principal Investigator’s Name (print)

____ ______________

Principal Investigator’s Signature Date

Please return a copy of the form as instructed by your local study monitor. Please retain the original for your study files.

PROTOCOL SYNOPSIS

**TITLE:**	**A PHASE II, RANDOMIZED, DOUBLE-BLIND, MULTICENTER, PARALLEL GROUP STUDY TO EVALUATE THE SUSTAINED VIROLOGIC RESPONSE OF THE HCV POLYMERASE INHIBITOR PRODRUG RO5024048 IN COMBINATION WITH BOCEPREVIR AND PEGASYS®/COPEGUS® IN PATIENTS WITH CHRONIC HEPATITIS C GENOTYPE 1 VIRUS INFECTION WHO WERE PRIOR NULL RESPONDERS TO TREATMENT WITH PEGYLATED INTERFERON/RIBAVIRIN**
**PROTOCOL NUMBER:**	NV27780
**EUDRACT NUMBER:**	2011-002714-37
**IND NUMBER:**	73,760
**TEST PRODUCT:**	RO5024048
**PHASE:**	II
**INDICATION:**	Chronic Hepatitis C (CHC)
**SPONSOR:**	F. Hoffmann–La Roche Ltd

Objectives

Primary Objective

The primary objective for this study is as follows:

- To estimate the sustained virologic response 12 weeks after treatment (SVR-12) for the two experimental treatment groups (regimens containing RO5024048, boceprevir, Pegasys, and Copegus) in patients with previous null response to PEG-IFN/RBV combination therapy, defined as a < 2 $\log_{10}$ IU/mL decrease in viral titer after at least 12 weeks of treatment with PEG-IFN/RBV

  RO5024048 in combination with boceprevir and Pegasys/Copegus administered for 24 weeks

  RO5024048 in combination with boceprevir and Pegasys/Copegus administered for 24 weeks followed by boceprevir and Pegasys/Copegus administered for 24 weeks

Secondary Objectives

The secondary objectives for this study are as follows:

- To estimate the sustained virologic response 24 weeks after treatment (SVR-24) for the two experimental treatment groups
- To estimate the sustained virologic response 4 weeks after treatment (SVR-4) for the two experimental treatment groups
- To estimate the virological response over time (all visits) for the experimental treatment groups
- To estimate the proportion of patients who develop resistance to boceprevir and/or RO5024048
- To compare the safety and tolerability of the treatment groups

Study Design

Description of Study

This Phase II, randomized, double-blind, parallel group design will have two experimental treatment groups (see table below). Genotype 1 PEG-IFN/RBV null responders will be randomized (1:1) into the experimental treatment groups. Randomization will be centralized and stratified by hepatitis C virus (HCV) genotype (1a or 1b) and interleukin 28B (IL28B) genetic polymorphism (CC, CT, or TT).

Group (N = 60)		Study Week			
		4	24	48	72
A n = 30	Screen	RO5024048 [a] + boceprevir [b] + Pegasys/ Copegus [c]		Treatment-free follow up	
B n = 30	Screen	RO5024048 [a] + boceprevir [b] + Pegasys/ Copegus [c]		Boceprevir [b] + Pegasys/ Copegus [c]	Treatment-free follow up

BID = twice daily; PO = orally; QD = once daily; QW = once weekly; SC = subcutaneous; TID = thrice daily.

Note: Screen up to *8* weeks; rescreen up to 35 days.

[a] RO5024048 1000 mg PO BID.

[b] Boceprevir 800 mg PO TID (at recommended intervals of 7–9 hours).

[c] Pegasys 180 µg SC QW + Copegus 1000 mg (< 75 kg) or 1200 mg (≥ 75 kg) PO QD.

Number of Patients

Approximately 60 patients who qualify for the study will be enrolled at approximately 35-40 sites. Patients enrolled prior to protocol version 4 and randomized to Group C will be unblinded and offered mericitabine, in addition to the treatment they are receiving.

Target Population

Patients must meet the following criteria for study entry:

1. Male or female aged 18 years and older
2. Serologic evidence of chronic hepatitis C (CHC) infection by an anti-HCV antibody (Ab) test (current or historical)
3. Evidence of CHC infection > 6 months duration
4. Serum HCV RNA quantifiable at ≥ 50,000 IU/mL as demonstrated by the Roche COBAS® TaqMan® HCV Test
5. Evidence of HCV genotype 1a or 1b infection by molecular assay

6. The following information related to the patient's response to the previous course of PEG-IFN/RBV therapy must be available in the medical records of the patient: (1) approved doses of prior PEG-IFN/RBV treatment and the start/end date of previous treatment with PEG-IFN/RBV, (2) documentation of previous dose modifications or interruptions (or lack thereof) to ensure documentation of previous compliance with therapy, (3) HCV RNA prior to the start of previous treatment and at 12 weeks after the start of treatment (window of Week 11 to Week 16) showing a null response, defined as a < 2 $\log_{10}$ IU/mL decrease in viral titer after at least 12 weeks of treatment with PEG-IFN/RBV, (4) HCV assay used, and (5) limit of detection of the assay used.
7. Patients must have discontinued prior HCV treatment at least 12 weeks prior to enrollment (receipt of first dose) in this trial.
8. Chronic liver disease consistent with CHC infection as seen via biopsy, using the scoring methods in Appendix B. Patients designated as *not* having cirrhosis or incomplete/transition to cirrhosis must have had a liver biopsy consistent with CHC within 24 calendar months of the first dose. For patients with cirrhosis or incomplete/transition to cirrhosis, there is no time frame for the biopsy. Fibroscan assessment is allowed in such patients but must be performed within 12 calendar months of the first dose. An elasticity score of ≥ 12.5 kPa will be used to designate incomplete/transition to cirrhosis or cirrhosis (i.e., 'cirrhotic'). If an elasticity score of ≥ 9.5 kPa but < 12.5 kPa is returned, the patient must receive a liver biopsy to determine whether his or her case is described as 'cirrhotic' or 'non-cirrhotic'.
9. Patients with cirrhosis or incomplete/transition to cirrhosis must have an abdominal ultrasound, computed tomography (CT) scan, or magnetic resonance imaging (MRI) scan without evidence of hepatocellular carcinoma (within 6 months prior to randomization), an endoscopy without evidence of bleeding gastroesophageal varices (within 2 years prior to randomization), and a serum alpha-fetoprotein (AFP) < 100 ng/mL (< 100 μg/L).
10. Normal cardiac troponin I value at the screening visit (< 0.100 ng/mL)
11. Serum total bilirubin less than the upper limit of normal (ULN) at the screening visit, unless there is a documented history of Gilbert's syndrome
12. Negative serum pregnancy test at screening (for females of childbearing potential). Negative urine pregnancy test documented within the 24-hour period prior to the first dose of study drugs confirmed by a negative serum pregnancy test collected within 24 hours prior to the first dose of study drug
13. Female patients of childbearing potential must agree to use two forms of non-hormonal contraception (i.e., condom, cervical barrier, intrauterine device [IUD], spermicide, or sponge) during treatment and after the last dose of boceprevir, RO5024048, and Copegus (in accordance with the locally approved label for Copegus). All males with female partners of childbearing potential must use two forms of effective contraception (combined) during treatment and following the last dose of Copegus in accordance with the locally approved label for Copegus.
14. Willingness to give written informed consent and willingness to participate in and comply with the study requirements

Patients who meet any of the following criteria will be excluded from study entry:

1. Infection with any HCV genotype other than genotype 1a or 1b
2. Body mass index < 18 or ≥ 36
3. Positive test at screening for anti–hepatitis A virus (HAV) IgM Ab, hepatitis B surface antigen (HBsAg), or anti-HIV Ab
4. History of having received RO5024048/boceprevir or any cross-resistant direct-acting antiviral (DAA) agent at any previous time or use of any other systemic antiviral therapy with established or perceived activity against HCV ≤ 3 months prior to the first dose of study drug
5. Herbal agents (e.g., milk thistle) ≤ 1 month prior to the first dose of study drug and throughout the duration of the study

6. History of having received any investigational drug ≤ 3 months prior to the first dose of study drug or the expectation that such drugs will be used during the study. Patients enrolled in this study cannot be simultaneously enrolled in another study for research, diagnostic, or treatment purposes.
7. History or other evidence of a medical condition associated with chronic liver disease other than HCV (e.g., hemochromatosis, autoimmune hepatitis, Wilson's disease, alpha-1 antitrypsin deficiency, alcoholic liver disease, and/or toxin exposure)
8. Females who are pregnant or breastfeeding
9. Males with female partners who are pregnant
10. Absolute neutrophil count (ANC) < 1.5×10^3 cells/μL (< 1.5×10^9 cells/L)
11. Platelet count < 90×10^3 cells/μL (< 90×10^9 cells/L)
12. Hemoglobin (Hgb) concentration < 12 g/dL (120 g/L) in females or < 13 g/dL (130 g/L) in males or a baseline increased risk for anemia (e.g., thalassemia, sickle cell anemia, spherocytosis, history of gastrointestinal bleeding) or in those for whom anemia would be medically problematic
13. The use of colony-stimulating factors such as granulocyte colony-stimulating factor (G-CSF), erythropoietin, blood transfusion, or other therapeutic agents to elevate hematology parameters to facilitate patient entry into the study within the last 6 months
14. Any patient with a history of severe psychiatric disease, including psychosis and/or depression, characterized by a suicide attempt, hospitalization for psychiatric disease, or a period of disability as a result of psychiatric disease who does not agree to have a psychiatric evaluation at screening and who does not agree to have continued monitoring by a mental health specialist at least every 4 weeks during the study
15. History of immunologically mediated disease (e.g., vasculitis, cryoglobulinemia, inflammatory bowel disease, idiopathic thrombocytopenic purpura, lupus erythematosus, autoimmune hemolytic anemia, scleroderma, severe psoriasis [defined as affecting > 10% of the body, where the palm of one hand equals 1% or if the hands and feet are affected], rheumatoid arthritis requiring more than intermittent non-steroidal anti-inflammatory medications for management). Patients with a history of celiac disease may enroll in the study.
16. History or other evidence of decompensated liver disease. Coagulopathy, hyperbilirubinemia, hepatic encephalopathy, hypoalbuminemia, ascites, and bleeding from esophageal varices are conditions consistent with decompensated liver disease.
17. Serum creatinine > 1.5 times the ULN
18. History of pre-existing renal disease. Patients with a history of nephrolithiasis will be allowed.
19. Estimated creatinine clearance (CRCL) of ≤ 70 mL/min (≤ 1.17 mL/sec), calculated by the Cockcroft-Gault formula (see Appendix C)
20. Type 1 or 2 diabetes with glycosylated hemoglobin (HbA1c) of ≥ 8.5% at the screening visit
21. History or other evidence of chronic pulmonary disease associated with functional limitation
22. History of severe cardiac disease (e.g., New York Heart Association Functional Class III or IV, myocardial infarction within 6 months, ventricular tachyarrhythmia requiring ongoing treatment, unstable angina, or other significant cardiovascular disease). Patients with stable cardiac disease (e.g., 6 months after bypass surgery, stent replacement, etc.) as confirmed by a cardiologist will be permitted. In addition, patients with documented or presumed coronary artery disease or cerebrovascular disease should not be enrolled if in the judgment of the investigator an acute decrease in Hgb by up to 4 g/dL would not be well tolerated.
23. Patients with higher potential of duration of ventricular depolarization and repolarization (corrected) (QTc) prolongation as defined by duration of ventricular depolarization and repolarization (QT) interval for heart rate using Fredericia's formula (QTcF) > 450 ms (average from multiple readings) or notable resting bradycardia < 50 beats/min or notable resting tachycardia > 100 beats/min or personal or family history of congenital long QT syndrome or sudden death
24. History of uncontrolled severe seizure disorder

25. History of any neoplastic disease within the last 5 years, with the exception of localized or in situ carcinoma of the skin (e.g., basal or squamous cell carcinoma)
26. History of any systemic antineoplastic or immunomodulatory treatment (including supraphysiologic doses of steroids and radiation) ≤ 6 months prior to the first dose of study drug or the expectation that such treatment will be needed at any time during the study
27. Poorly controlled thyroid dysfunction
28. History or other evidence of a clinically relevant ophthalmologic disorder due to diabetes mellitus or hypertension or history or other evidence of severe retinopathy (e.g., cytomegalovirus, macular degeneration)
29. History of major organ transplantation with an existing functional graft
30. History or other evidence of severe illness or any other conditions that would make the patient, in the opinion of the investigator, unsuitable for the study
31. Use of contraindicated concomitant medications that are highly dependent on cytochrome P450 (CYP) 3A4/5 for clearance and for which elevated plasma concentrations are associated with serious and/or life-threatening events. Potent CYP3A4/5 inducers are also contraindicated (Victrelis™ USPI 2011).
32. Evidence of excessive alcohol, drug, or substance abuse (excluding marijuana use) within 1 year of the first dose
33. History of allergy to RBV, pegylated IFNs, or other IFNs

Length of Study

The length of study will be approximately 2 years from screening of the first patient to the end of the study as described below.

End of Study

The end of the study is defined as the date when the last patient last visit (LPLV) occurs. Last patient last visit is defined as either the date of the last patient visit of the last patient to complete the study or the date at which the last data point from the last patient—which was required for statistical analysis (i.e., key safety and efficacy results for decision-making)—was received, whichever is the later date.

<u>Efficacy Outcome Measures</u>

Primary Efficacy Outcome Measure

The primary efficacy endpoint will be SVR-12 (actual) outcome with response defined as an unquantifiable (less than the lower limit of quantification [LLOQ; < 25 IU/mL]) serum HCV RNA 12 weeks after the actual end of treatment (a single last unquantifiable HCV RNA within 8–20 weeks after the last day of study drug administration).

Secondary Efficacy Outcome Measures

For the following measures, which are HCV RNA response outcomes, response is defined as an unquantifiable (less than the LLOQ [< 25 IU/mL]) serum HCV RNA:

- Hepatitis C virus RNA response outcome at clinic visits over time
- Sustained virologic response 24 weeks after treatment (SVR-24) (actual) outcome, defined as the HCV RNA response outcome ≥ 20 weeks after the last day of study drug administration
- Sustained virologic response 4 weeks after treatment (SVR-4) (actual) outcome, defined as the HCV RNA response outcome within 2–8 weeks after the last day of study drug administration
- Hepatitis C virus RNA (log_{10} IU/mL) change from baseline to Week 24
- Occurrence of documented DAA resistance

Safety Outcome Measures

- Incidence and severity of adverse events (AEs) and serious adverse events (SAEs) and reasons for discontinuation of any study medication. The Division of AIDS Table for Grading the Severity of Adult and Pediatric Adverse Events (ACTG) will be used to grade AEs.
- Clinically significant changes in vital signs, laboratory tests, and ECG results from screening/baseline

Pharmacokinetic Outcome Measures

Trough concentrations for RO4995855 (parent drug of RO5024048), its metabolite (RO5012433), and boceprevir.

Exploratory Outcome Measures

Interferon gamma-induced protein 10kDa levels will be measured at baseline in all patients to evaluate whether the baseline IP-10 levels affect treatment response.

Investigational Medicinal Products

Test Products

RO5024048 1000 mg orally (PO) twice daily (BID) for 24 weeks

Boceprevir 800 mg PO thrice daily (TID) (at recommended intervals of 7–9 hrs) for 24 or 48 weeks

Pegasys 180 µg via subcutaneous (SC) route once weekly (QW) for 24 or 48 weeks

Copegus 1000 mg (< 75 kg) or 1200 mg (≥ 75 kg) PO once daily (QD) for 24 or 48 weeks

Statistical Methods

Primary and secondary efficacy analyses will include all patients who were randomized and received at least one dose of study medication. Patients will be analyzed according to the treatment group to which they were randomized.

No formal hypothesis testing will be done in this exploratory study. As a result, no adjustment for a Type 1 error will be made to account for the multiplicity of analyses.

Primary Analysis

The primary efficacy endpoint (SVR-12 [actual]) for each of the treatment groups will be summarized using descriptive statistics (n, percentage, and confidence interval based upon binomial probabilities).

Determination of Sample Size

Boceprevir with Pegasys/Copegus has been shown to provide approximately 34% sustained virologic response (SVR) in patients with poor prior response defined as < 1 $\log_{10}$ IU/mL decline after a 4 week lead in with PEG-IFN/RBV (Bacon et al. 2011). An SVR rate of 45%–55% for an experimental group is expected on the basis of an anticipated reduction in treatment-emergent resistance to boceprevir and the potential added benefit of RO5024048.

Assuming response rates of 45%–55% for an experimental treatment group, a sample size of 30 patients per experimental treatment group will provide 90% confidence interval widths of ± 15%.

Interim Analyses

At least one interim analysis of efficacy and safety data will be performed to inform the RO5024048 clinical development plan. An interim analysis will be performed for safety after all patients complete Week 12, and for safety and efficacy after all patients complete Week 36 and potentially at other timepoints as needed. Virologic response with a confidence interval will be summarized by visit. Presentations of resistance rates and of safety data including tabulations of all AEs, all laboratory abnormalities, and reasons for dose modifications and/or discontinuation from trial medications will be prepared.

LIST OF ABBREVIATIONS AND DEFINITIONS OF TERMS

Abbreviation	Definition
Ab	antibody
ACTG	The Division of AIDS Table for Grading the Severity of Adult and Pediatric Adverse Events
AE	adverse event
AFP	alpha-fetoprotein
AKR	aldo-keto reductase
AMA	antimitochondrial antibody
ANA	antinuclear antibody
ANC	absolute neutrophil count
aPTT	activated partial thromboplastin time
ASMA	antismooth muscle antibody
AT	antitrypsin
AUC	area under the concentration–time curve for a dosing interval
BID	twice daily
BP	blood pressure
BSA	body surface area
cDNA	complementary DNA
CHC	chronic hepatitis C
CK	creatinine kinase
C_{max}	maximum observed drug concentration during a dosing interval
C_{trough}	minimum observed drug concentration during a dosing interval
CRCL	creatinine clearance
CRF	Case Report Form
CT	computed tomography
CYP	cytochrome P450
DAA	direct-acting antiviral
EC	Ethics Committee
EC_{50}	half maximum effective concentration
EC_{90}	concentration required for 90% growth inhibition of treated cells
eCRF	electronic Case Report Form
EDC	electronic data capture
ESA	erythropoiesis-stimulating agent
FC	fold change

Abbreviation	Definition
FDA	Food and Drug Administration
G-CSF	granulocyte colony-stimulating factor
HAV	hepatitis A virus
HbA1c	glycosylated (or glycated) hemoglobin
HBsAg	hepatitis B surface antigen
HCV	hepatitis C virus
Hgb	hemoglobin
HCT	hematocrit
HIPAA	Health Insurance Portability and Accountability Act
IB	Investigator's Brochure
IC_{50}	50% inhibitory concentration
ICH	International Conference on Harmonisation
IFN	interferon
IL28B	interleukin 28B
IMC	Internal Monitoring Committee
IMP	investigational medicinal product
IND	Investigational New Drug Application
INR	International Normalized Ratio
IP-10	interferon gamma-induced protein 10kDa
IRB	Institutional Review Board
IU	international unit
IUD	intrauterine device
IVRS	interactive voice response system
LC/MS/MS	liquid chromatography tandem mass spectrometry
LLOQ	lower limit of quantification
LPLV	last patient last visit
MCH	mean corpuscular hemoglobin
MCHC	mean corpuscular hemoglobin concentration
MCV	mean corpuscular volume
MRI	magnetic resonance imaging
mRNA	messenger RNA
NS3	non-structural protein 3
NS4A	non-structural protein 4A
NS5A	non-structural protein 5A
NS5B	non-structural protein 5B
PCR	polymerase chain reaction
PD	pharmacodynamic

Abbreviation	Definition
PEG-IFN	polyethylene glycol conjugated (pegylated) interferon alfa
P-gp	P-glycoprotein
PK	pharmacokinetic
PO	orally
PT	prothrombin time
PW	premature withdrawal (from study)
QD	once daily
QT	duration of ventricular depolarization and repolarization
QTc	QT corrected
QTcF	QT interval for heart rate using Fredericia’s formula
QUAD	four drug
QW	once weekly
RBV	ribavirin
RCR	Roche Clinical Repository
SAE	serious adverse event
Roche Guideline	International Guideline for the Handling and Reporting of Laboratory Data
SAP	statistical analysis plan
SC	subcutaneous
SI	International System
SNP	single nucleotide polymorphism
SVR	sustained virologic response
SVR-4	sustained virologic response 4 weeks after treatment
SVR-12	sustained virologic response 12 weeks after treatment
SVR-24	sustained virologic response 24 weeks after treatment
TID	thrice daily
t_{max}	time to reach maximum drug concentration
TSH	thyroid-stimulating hormone
ULN	upper limit of normal

1. BACKGROUND

1.1 BACKGROUND ON CHRONIC HEPATITIS C

Hepatitis C virus (HCV) is responsible for a large proportion of chronic liver disease worldwide and accounts for 70% of cases of chronic hepatitis in industrialized countries. The global prevalence of chronic hepatitis C (CHC) is estimated to be approximately 3% (ranging from 0.1% to 5%), with an estimated 170 million chronic carriers worldwide (2.7 million in the United States and 5 million in Western Europe) (Lavanchy 1999). Approximately one-fifth of chronically infected HCV patients eventually develop cirrhosis, which may lead to liver failure and hepatocellular carcinoma (EASL 1999). Hepatitis C virus–related liver failure is one of the primary reasons for liver transplantation today.

Before the recent approval of HCV protease inhibitors, polyethylene glycol conjugated (pegylated) interferon (IFN) alfa (collectively, PEG-IFN) in combination with ribavirin (RBV) was the recommended first-line treatment for patients with CHC (NIH 2002), and approximately 50% of patients are able to achieve a sustained virologic response (SVR) (defined in this case as undetectable serum HCV RNA 24 weeks after the completion of therapy) with a first course of therapy (Fried et al. 2002; Hadziyannis et al. 2004; Manns et al. 2001; Gale and Foy 2005; Lauer and Walker 2001).

The HCV genotype has been identified as the most important viral factor influencing the response to antiviral therapy (Zeuzem 2004). Hepatitis C virus genotype 1, the major genotype, accounts for approximately 70% of HCV infections in the United States and Western Europe and is the most difficult to treat, with an SVR rate < 50% (Daniel 2005; Bukh et al. 1995). Patients infected with HCV genotypes 2 and 3 respond much better (SVR rate of 70%–80%) to PEG-IFN/RBV combination therapy than those infected with genotype 1 (Mauss et al. 2010). Patients infected with HCV genotype 4 respond worse than patients infected with genotypes 2 and 3, but appear to respond better than patients with genotype 1 (Kamal and Nasser 2008). Because patients with genotype 1 have a lower response rate compared with other genotypes, the treatment failure group is predominantly populated with genotype 1 patients.

Re-treatment of the growing population of patients who have previously failed a course of PEG-IFN/RBV with a further course of PEG-IFN/RBV has met with limited success. Fewer than 1 in every 10 previous non-responder patients has achieved an SVR with 48 weeks of re-treatment (Jensen et al. 2009; Dieterich et al. 2009). Current U.S. treatment guidelines (Ghany et al. 2009) do not advocate re-treatment of non-responders. Although re-treatment of non-responders is approved in the European Union (SmPC eMC 2010), a recommended 72-week treatment duration for genotype 1 patients and the fact that most re-treated patients meet the Week 12 stopping rule for futility of further treatment make this treatment of limited utility.

Efforts to improve patient outcomes have focused on direct-acting antiviral (DAA) small molecules that inhibit specific viral-encoded enzymes, such as the HCV protease or

HCV polymerase (Soriano et al. 2009; Powdrill et al. 2010; Chatel-Chaix et al. 1999). Promising results have been published or presented at international meetings from the Phase II and Phase III studies with the most advanced protease inhibitors either currently in development or recently approved (McHutchison et al. 2010; Zeuzem et al. 2010). Each DAA is being developed in combination with PEG-IFN/RBV because of the improved potency and reduction in potential resistance development compared with its administration as monotherapy (Sarrazin and Zeuzem 2010). Emergence of resistance is of particular concern when a single DAA with a low barrier to resistance (e.g., protease inhibitors or non-nucleoside polymerase inhibitors) is used for the treatment of PEG-IFN/RBV null responders, where the PEG IFN/RBV backbone contributes substantially less activity to the regimen (Adijawah et al. 2009; Rong et al. 2010). Observations about resistance (Sarrazin and Zeuzem 2010), along with lessons learned from HIV therapy (Monto et al. 2010), suggest that combinations of DAAs may be critical to limiting development of resistance and improving virologic response in the most difficult to-treat patients. Despite these concerns, improvements in SVR in PEG-IFN/RBV treatment-failure patients have been reported with the addition of a single HCV protease inhibitor to PEG-IFN/RBV (McHutchison et al. 2010).

Two protease inhibitors (telaprevir and boceprevir) were recently approved for treatment-naïve and treatment-experienced CHC genotype 1 patients. Despite significant improvement in response rates for treatment-naïve patients, there continues to be an unmet medical need for partial and null responders, particularly for those with compensated cirrhosis.

1.2 BACKGROUND ON TEST MOLECULES

1.2.1 HCV Polymerase Inhibitor Prodrug (RO5024048)

RO5024048, a diisobutyl ester prodrug of the cytidine nucleoside analog RO4995855 (beta-D-2′-deoxy-2′-fluoro-2′-C-methycytidine, also known as PSI-6130 and referred to as the parent drug), is a potent, competitive, and highly selective nucleoside inhibitor of non-structural protein 5B (NS5B)-directed HCV polymerase, with a 50% inhibitory concentration (IC50) value of 0.61 ± 0.04 μM in the HCV replicon system (Roche 2006). Combination with either Pegasys® or Copegus® produces an additive antiviral effect. Activity has been demonstrated in vitro and in vivo across the four main genotypes (1–4).

RO4995855 has been assessed in the replicon system and has been shown to potently inhibit HCV replication with the concentration required for 90% growth inhibition of treated cells (EC_{90}) ranging from approximately 1 to 5 μM. The elimination route for RO4995855 and RO5012433 is predominantly via the kidney. The prodrug RO5024048 was developed because of its superior permeability leading to increased plasma exposure of RO4995855 and thereby improved antiviral efficacy. The nonclinical and early clinical assessment of RO5024048 supports its promise as an effective and selective antiviral drug for the treatment of chronic HCV infection (RO5024048 Investigator’s Brochure [IB] 2011).

Toxicology and safety pharmacology studies were conducted with RO5024048 and RO4995855. RO5024048 was generally well tolerated in rodents and dogs in single and repeat-dose toxicity studies up to 26 and 4 weeks duration, respectively. Hematologic changes were evident with 4 weeks of dosing in monkeys (decreased RBCs, hemoglobin (Hgb), and hematocrit (HCT). Renal toxicity, with evidence of microangiopathic glomerulopathy was noted in monkeys at doses ≥ 25 mg/kg/day after 13 and 26 weeks of treatment. At the end of 8–16 weeks of recovery following dosing for 13–26 weeks, the glomerular changes were reversing and tended to be less severe than those at the end of treatment, with no fibrosis. After a recovery period following 36 weeks of dosing, the glomerular changes had completely reversed. Secondary cardiac abnormalities (heart murmurs, left ventricular hypertrophy, or increased heart weight) at doses of 600 mg/kg/day (13-week study) and 100–400 mg/kg/day (26-week study) were speculated to be secondary to renal hypertension associated with glomerular changes. To date, no evidence of clinical renal injury (as measured by changes in serum creatinine or urine protein/creatinine ratio) has been seen with up to 24 weeks of dosing of RO5024048 in patients with chronic HCV infection. A comprehensive summary of the nonclinical pharmacology, absorption, distribution, metabolism, and excretion and safety profile are provided in the RO5024048 IB.

Clinical Phase I experience with RO5024048 was gained from a three-part study conducted by Pharmasset, Inc., to investigate safety, pharmacokinetics, and pharmacodynamics in healthy volunteers and in patients chronically infected with HCV genotypes 1, 2, or 3 (Protocol P7081-5101) and is summarized in the RO5024048 IB.

There are two randomized, placebo-controlled, multicenter Phase II studies to evaluate dose and duration of RO5024048 in treatment-naïve CHC genotype 1 and 4 patients. All patients in both studies have completed treatment with RO5024048 in combination with Pegasys/Copegus. In Study NV20536, 408 treatment-naïve HCV genotype 1 or 4 patients received twice-daily (BID) RO5024048 placebo, 500 mg, or 1000 mg in combination with Pegasys/Copegus for 8 or 12 weeks followed by 12–36 weeks of Pegasys/Copegus, depending on early response. Study NV22621 enrolled 166 treatment-naïve HCV genotype 1 or 4 patients to receive RO5024048 placebo or 1000 mg BID in combination with Pegasys/Copegus for 24 weeks followed by up to 24 weeks of Pegasys/Copegus, depending on early response.

Safety data indicate that RO5024048 (500 or 1000 mg BID) was well tolerated when given in combination with Pegasys/Copegus for up to 24 weeks. To date, there have been no significant safety issues identified and no significant drug-related toxicity findings. Specifically, there was no evidence of an increase in Grade 3 or 4 cytopenias, abnormalities of renal parameters, or ocular or gastrointestinal events in the RO5024048 treatment groups. Serious adverse events (SAEs) were equally distributed across all treatment groups, including the Pegasys/Copegus control group (RO5024048 IB 2011).

1.2.2 HCV Protease Inhibitor (Boceprevir)

Boceprevir is a structurally novel peptidomimetic ketoamide protease inhibitor that binds reversibly to the HCV non-structural 3 (NS3) active site, inhibiting viral replication. Boceprevir is mainly metabolized by the aldo-keto reductase (AKR) pathway and also undergoes oxidative metabolism by cytochrome P450 (CYP) 3A4/5, making boceprevir a potent CYP3A4/5 inhibitor (Ghosal et al. 2011).

Based on available information, the findings identified in general toxicology studies with boceprevir include neutrophil infiltrates in the liver of mice and multinucleated hepatocytes in rats. Testicular degeneration was observed in male rats, but not in male mice or monkeys. These findings are considered species specific in nature and occur at exposures that are slightly higher than or similar to the intended clinical therapeutic dose. Increases in activated partial thromboplastin time (aPTT) have been observed in monkey toxicity studies; in the absence, however, of gross pathology evidence of hemorrhage. Based on preclinical toxicity profile for boceprevir, no overlapping target organ of toxicity with RO5024048 is expected.

Boceprevir is not expected to affect the pharmacokinetics of RO5024048 or vice versa. Boceprevir has been identified in vitro as an inhibitor of CYP3A and P-glycoprotein (P-gp); however, RO5024048 is not metabolized by CYP3A and is not a P-gp substrate. RO5024048 and RO4995855 (parent) did not show any relevant in vitro CYP inhibition or induction (RO4995855 only) potential that may affect boceprevir. RO5024048 has not been assessed as an inhibitor of AKRs, the major boceprevir metabolizing enzyme. However, because of the ubiquitous nature of AKRs and the lack of clinical interaction shown in a drug–drug interaction study with boceprevir using an AKR inhibitor, a drug–drug interaction is very unlikely (Boceprevir IB 2012).

A Phase I study comparing boceprevir monotherapy, PegIntron® monotherapy, and boceprevir plus PegIntron combination therapy in genotype 1 patients who failed previous treatment with PegIntron with or without RBV showed that combination therapy with boceprevir and PegIntron produced greater reductions in viral load than either drug given as monotherapy (Berman et al. 2009; Sarrazin et al. 2007).

Resistant variants, which lead to breakthrough, have been identified when protease inhibitors are given as monotherapy (Sarrazin and Zeuzem 2010; Susser et al. 2009). In Phase II and Phase III studies, boceprevir was given in combination with PegIntron/Rebetol® to reduce the emergence of resistant variants (Susser et al. 2009). Additionally, a 4-week lead-in of PegIntron/Rebetol was introduced prior to initiation of boceprevir triple therapy to reduce the emergence of resistant variants by reducing viral loads (Susser et al. 2009; Kwo et al. 2010). Phase III boceprevir studies in HCV genotype 1 treatment-naïve and previous treatment-failure population, consisting of prior partial responders and those who had relapsed, showed SVR rates of 68% and 66%, respectively, in patients who received 44 weeks of triple therapy after a 4-week lead-in of PegIntron/Rebetol (Poordad et al 2011; Bacon et al. 2011).

1.2.3 Pegasys (Peginterferon Alfa-2a)

Roche chemically modified the IFN alfa-2a molecule by covalently attaching a branched methoxy polyethylene glycol moiety (Pegasys IB 2010). Pegasys has a decreased systemic clearance rate and an approximately 10-fold increase in serum half-life compared with IFN alfa-2a, and as a result Pegasys circulates in the blood much longer than the parent compound. Subsequent evaluation of Pegasys given 180 μg once weekly (QW) in three large clinical trials in > 1400 patients showed that treatment with Pegasys was more efficacious than treatment with IFN thrice weekly (Pegasys USPI 2011).

1.2.4 Copegus (Ribavirin)

Ribavirin is a guanosine analog that inhibits the in vitro replication of a wide range of RNA and DNA viruses (Sidwell et al. 1979). The mechanism by which RBV acts as an antiviral is not fully defined, although it may involve alteration of cellular nucleotide pools and inhibition of viral RNA synthesis (Gilbert and Knight 1986). Ribavirin monotherapy has little or no effect on the replication of HCV, but it can result in the normalization of serum ALT activity and improvement in liver histology. However, relapse occurs in nearly all patients treated with RBV alone (Dusheiko et al. 1996; Di Bisceglie et al. 1995).

Combining Copegus with Pegasys has been found to be more effective than Pegasys monotherapy in the treatment of CHC. In a large clinical trial of 1121 patients of all genotypes, an SVR was achieved in 53% of patients treated with Pegasys/Copegus as compared with 29% of patients treated with Pegasys alone (Pegasys IB 2010). Recent data suggest that Copegus may provide a similar clinical benefit when combined with Pegasys and boceprevir and when added to an IFN-free DAA regimen (Zeuzem et al. 2010).

1.3 STUDY RATIONALE AND BENEFIT–RISK ASSESSMENT

Despite improvements in outcomes for CHC patients over the past decade, response rates with PEG-IFN/RBV in genotype 1 null-responder patients remain suboptimal, and treatment is associated with considerable side effects. Patients who failed an initial course of PegIntron®/Rebetol® and were re-treated with Pegasys/Copegus achieved an SVR of only 16% even with an increased dose of 360 μg Pegasys during the first 12 weeks and longer treatment duration of 72 weeks (Jensen et al. 2009). Even with the development of protease inhibitors such as telaprevir and boceprevir, considerable improvement for prior non-responders to PEG-IFN/RBV is still needed.

A Phase II boceprevir study in genotype 1 null responders and non-responders showed an SVR of 14% (7/49) when boceprevir was given at a dose of 400 mg thrice daily (TID) in combination with PegIntron/Rebetol (Schiff et al. 2008). In RESPOND-2, a Phase III boceprevir study, patients with non-response—defined as a decrease in viral load of at least 2 log10 IU/mL by Week 12 but a detectable viral load throughout the course of therapy—achieved an SVR of 52% after 48 weeks of triple therapy. In the same study,

an SVR of 34% was seen in patients who had a poor response to IFN—defined as a < 1 log10 IU/mL viral load decline—after a 4-week lead-in period of PegIntron/Rebetol alone followed by triple combination therapy with boceprevir for a total duration of 48 weeks (Bacon et al. 2011). Although the addition of a protease inhibitor to PEG-IFN/RBV improves treatment efficacy, there remains a substantial medical need to improve the therapeutic options for the genotype 1 null-responder patient population, particularly those with cirrhosis.

The RESPOND 2 study was conducted to evaluate boceprevir (BOC)/PEG-IFN/RBV therapy in prior PEG-IFN/RBV relapsers and non-responders. Though null responders were excluded, subset analysis of patients with < 1 log reduction in HCV RNA following the 4-week PEG-IFN/RBV lead-in period may be considered as a surrogate population for null responders. Data on null responders was obtained in the PROVIDE study, which enrolled subjects who were observed to be null responders while receiving PEG-IFN/RBV in control arms of BOC studies; these subject subsequently received BOC/PEG-IFN/RBV. From the above sources of data, estimates of SVR with BOC-containing regimens in these poorly responsive subjects ranged from 28%-40% (Merck presentation to AVDAC, 27 April 2011; Bronowicki et al. EASL, 2012). No published data on null cirrhotic patients are available.

2. OBJECTIVES

2.1 PRIMARY OBJECTIVE

The primary objective for this study is as follows:

- To estimate the sustained virologic response 12 weeks after treatment (SVR-12) for the two experimental treatment groups (regimens containing RO5024048, boceprevir, Pegasys, and Copegus) in patients with previous null response to PEG-IFN/RBV combination therapy, defined as a < 2 $\log_{10}$ IU/mL decrease in viral titer after at least 12 weeks of treatment with PEG-IFN/RBV

    RO5024048 in combination with boceprevir and Pegasys/Copegus administered for 24 weeks

    RO5024048 in combination with boceprevir and Pegasys/Copegus administered for 24 weeks followed by boceprevir and Pegasys/Copegus administered for 24 weeks

2.2 SECONDARY OBJECTIVES

The secondary objectives for this study are as follows:

- To estimate the sustained virologic response 24 weeks after treatment (SVR-24) for the two experimental treatment groups
- To estimate the sustained virologic response 4 weeks after treatment (SVR-4) for the two experimental treatment groups
- To estimate the virological response over time (all visits) for the experimental treatment groups
- To estimate the proportion of patients who develop resistance to boceprevir and/or RO5024048
- To compare the safety and tolerability of the treatment groups

2.3 EXPLORATORY OBJECTIVE

The exploratory objective for this study is as follows:

- To evaluate whether the baseline IP-10 levels affect treatment response

3. STUDY DESIGN

3.1 DESCRIPTION OF STUDY

This Phase II, randomized, double-blind, parallel group design will have two experimental treatment groups (see Table 1).

Genotype 1 PEG-IFN/RBV null responders will be randomized (1:1) into the experimental treatment groups. Randomization will be centralized and stratified by HCV genotype (1a or 1b) and interleukin 28B (IL28B) genetic polymorphism (CC, CT, or TT).

A screening period (time from the first screening assessment to the first administration of test drug) of up to 8 weeks will precede the treatment portion of the trial. If screening tests are conducted > 35 days prior to the baseline visit, certain tests will be repeated to confirm eligibility prior to enrollment.

Table 1 Study Design

Group (N = 60)		Study Week		
		4 24	48	72
A n = 30	Screen	RO5024048 [a] + boceprevir [b] + Pegasys/ Copegus [c]	Treatment-free follow up	
B n = 30	Screen	RO5024048 [a] + boceprevir [b] + Pegasys/ Copegus [c]	Boceprevir [b] + Pegasys/ Copegus [c]	Treatment-free follow up

BID = twice daily; PO = orally; QD = once daily; QW = once weekly; SC = subcutaneous; TID = thrice daily.

Note: Screen up to 8 weeks; rescreen up to 35 days.

[a] RO5024048 1000 mg PO BID.

[b] Boceprevir 800 mg PO TID (at recommended intervals of 7–9 hours).

[c] Pegasys 180 µg SC QW + Copegus 1000 mg (< 75 kg) or 1200 mg (≥ 75 kg) PO QD.

To facilitate early and close monitoring of safety parameters over time, an Internal Monitoring Committee (IMC) composed of RO5024048 team members will review aggregate safety data on an ongoing basis as defined in the IMC charter.

This study is based in the United States, Canada, and the European Union.

An interim analysis for safety will be performed when all patients reach Week 12, and for safety and efficacy when all patients reach Week 36 and potentially at other timepoints as deemed necessary by the Sponsor.

3.2 END OF STUDY

The end of the study is defined as the date when the last patient last visit (LPLV) occurs. Last patient last visit is defined as either the date of the last patient visit of the last patient to complete the study or the date at which the last data point from the last patient—which was required for statistical analysis (i.e., key safety and efficacy results for decision-making)—was received, whichever is the later date.

3.3 RATIONALE FOR STUDY DESIGN

Studies are currently investigating the benefit of a four-drug (QUAD) combination to increase rates of SVR. One trial examining QUAD therapy with a protease inhibitor (BMS-650032) and a non-structural protein 5A (NS5A) inhibitor (BMS-790052) in combination with PEG-IFN/RBV for 24 weeks has shown SVR-12 in 100% (10/10) and sustained virologic response 24 weeks after treatment (SVR-24) in 90% (9/10) of previous null responders to PEG-IFN/RBV (Lok et al. 2011). Another QUAD study in null responders to PEG-IFN/RBV is assessing HCV RNA levels before, during, and after

treatment with BMS-650032 and BMS-790052 plus PEG-IFN/RBV for 24 weeks, with and without an additional 24 weeks of PEG-IFN/RBV.

The safety and efficacy of QUAD regimens are also being studied in treatment-naïve patients. In one study, 86% (51/59) of genotype 1 patients achieved extended rapid virologic response when treated with boceprevir and a non-nucleoside polymerase inhibitor (VX-222) plus Pegasys/Copegus for 12 weeks of response-guided therapy with an additional 12 weeks of Pegasys/Copegus in patients who did not reach a predefined viral response (Di Bisceglie et al. 2011). In another study, 92% (12/13) of treatment-naïve genotype 1 patients had undetectable HCV RNA at Week 24 when treated with a protease inhibitor (GS-9256) and a non-nucleoside polymerase inhibitor (GS-9190) plus PEG-IFN/RBV for 28 days followed by PEG-IFN/RBV alone for up to 48 weeks (Foster et al. 2011). A third study is examining SVR in treatment-naïve genotype 1 patients being treated with GS-9256 and GS-9190 plus PEG-IFN/RBV for 16 or 24 weeks of response-guided therapy with the option to continue PEG-IFN/RBV for up to 48 weeks.

Developing combinations of two DAA molecules as part of a QUAD regimen with PEG-IFN/RBV will likely improve response rates and/or shorten treatment duration when compared with PEG-IFN/RBV plus one DAA, particularly in a population with considerable treatment experience.

RO5024048 in combination with PEG-IFN/RBV, is active against all genotypes and no evidence of baseline or treatment emergent resistance with the combination has been seen to date (Jensen et al. 2010; Le Pogam et al. 2010), making it an attractive DAA to study in QUAD combination with protease inhibitors, such as boceprevir. RO5024048, studied in combination with the protease inhibitor danoprevir in the INFORM-1 study (i.e., IFN-free), showed treatment-emergent RO5024048 resistance in one genotype 1 HCV treatment-naïve patient (Gane et al. 2010). The combination of RO5024048 and danoprevir with RBV dramatically reduced resistance-associated virologic breakthrough compared to monotherapy (Sarrazin et al. 2009).

This study is designed to evaluate the SVR achieved from a QUAD regimen of RO5024048 with boceprevir in combination with Pegasys/Copegus in patients with CHC genotype 1 virus infection who were null responders, defined as having a $< 2 \log_{10}$ IU/mL decrease in viral titer after 12 weeks of prior treatment with PEG-IFN/RBV. Previous PEG-IFN/RBV null responders with compensated cirrhosis are the most difficult to treat and have the highest unmet medical need with the potential to benefit from treatment with a QUAD regimen. Furthermore, the study in genotype 1 null responders reduces the heterogeneity of responses that are expected among a broader non-responder CHC population and provides an opportunity to demonstrate the incremental antiviral benefits of adding RO5024048 using smaller sample sizes.

3.3.1 Rationale for Test Product Dosage

In a Phase I study (P7081-5101), the maximum antiviral activity of RO5024048 in combination with Pegasys/Copegus for 28 days was observed following administration of 1000 mg BID. There was no increase in antiviral effect or additive benefit seen with the higher dose of 1500 mg BID (see Figure 1). In combination with Pegasys/Copegus in treatment-naïve patients, a high on-treatment virologic response with 1000 mg BID was observed and was independent of IL28B genotype in Study NV22621 (Pockros et al. 2011). Therefore, a dose higher than 1000 mg BID would not be expected to provide any additional antiviral activity. In Study NV20536, RO5024048 1000 mg BID administered in combination with Pegasys/Copegus for 12 weeks was associated with higher levels of on-treatment viral suppression and a greater decrease in HCV RNA during the first 12 weeks of treatment than were observed with RO5024048 500 mg BID, particularly in non-CC genotype 1 patients (Jensen et al. 2010). Therefore, the 1000-mg dose was chosen because of the high prevalence of the unfavorable IL28B genotype in the target population.

Figure 1 Exposure–Response Curve for RO4995855 (Ctrough): Study 7081-5101

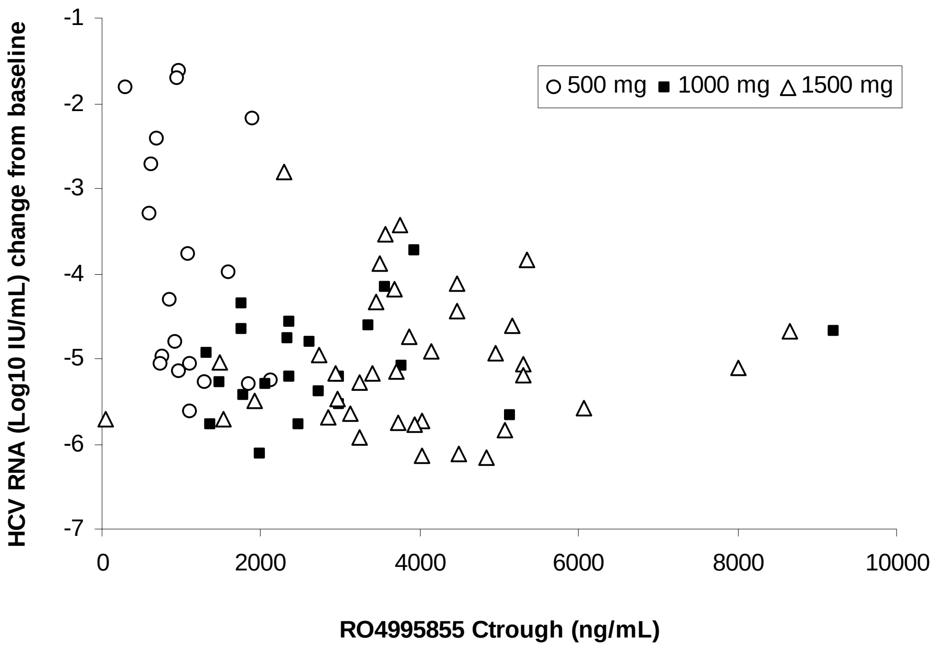

C_{trough} = minimum observed drug concentration during a dosing interval; HCV RNA = hepatitis C virus RNA.

The selected boceprevir dose in this study (800 mg thrice daily [TID]) is the currently approved dose for the treatment of CHC genotype 1 patients.

The doses selected for Pegasys/Copegus are based on currently approved doses for the treatment of CHC genotype 1 patients.

In Phase III studies of boceprevir, boceprevir was given for a maximum of 48 weeks. The preliminary results from Phase II studies of RO5024048 (NV20536 and NV22621) support RO5024048 treatment for up to 24 weeks. This study will examine if 24 weeks of RO5024048 and boceprevir is sufficient to achieve SVR when used in a QUAD regimen. The Pegasys/Copegus approved duration for the treatment of CHC genotype 1 patients is 48 weeks. This study will examine if a shorter duration of Pegasys/Copegus is sufficient to achieve SVR when used in a QUAD regimen. Therefore, in this study, boceprevir will be given for 24 or 48 weeks, RO5024048 will be given for 24 weeks, and Pegasys/Copegus will be given for 24 or 48 weeks.

3.3.2 Rationale for Patient Population

The patient population of genotype 1 previous null responders to PEG-IFN/RBV therapy was chosen because it has the highest unmet medical need with the potential to benefit from treatment with a QUAD regimen of two DAAs in combination with Pegasys/Copegus.

3.3.3 Rationale for Biomarker Assessments

Hepatitis C virus genotype 1 patients with different alleles (CC, CT, or TT) at one single nucleotide polymorphism (SNP) location (rs12979860) positioned upstream of the IL28B gene respond differently to PEG-IFN and RBV treatment, with CC being the best responder (Ge et al. 2009; Thompson et al. 2010). Although the C allele frequency of rs12979860 SNP varies from approximately 20% to more than 90% in population genetic sampling across the world (Khakoo et al. 2009), it is expected that the treatment-failure patient population will be enriched for patients with TT and CT genotype. Stratification of patients based on IL28B-related SNP will be required for balancing treatment arms for new investigative medicine trials involving PEG-IFN and/or RBV. Stratification based on IL28B-related SNP may help to assess the effect of IL28 genotype on treatment with DAAs.

Interferon gamma-induced protein 10kDaα is a baseline predictor (independent of IL28B) of IFN response. Plasma levels of IP-10 can be considered a surrogate marker for hepatic IFN-stimulated gene levels during HCV infection because the bulk of IP-10 is being secreted by the liver. IP-10 levels decrease during IFN/RBV or DAA treatment. Exploratory measurement of baseline IP-10 could provide information on whether baseline IP-10 levels affect treatment response.

3.4 OUTCOME MEASURES

3.4.1 Efficacy Outcome Measures

3.4.1.1 Primary Efficacy Outcome Measure

The primary efficacy endpoint will be SVR-12 (actual) outcome with response defined as an unquantifiable (less than the LLOQ [< 25 IU/mL]) serum HCV RNA 12 weeks after the actual end of treatment (a single last unquantifiable HCV RNA within 8–20 weeks after the last day of study drug administration).

3.4.1.2 Secondary Efficacy Outcome Measures

For the following measures, which are HCV RNA response outcomes, response is defined as an unquantifiable (less than the LLOQ [< 25 IU/mL]) serum HCV RNA:

- Hepatitis C virus RNA response outcome at clinic visits over time
- Sustained virologic response 24 weeks after treatment (SVR-24 actual) outcome, defined as the HCV RNA response outcome ≥ 20 weeks after the last day of study drug administration
- Sustained virologic response 4 weeks after treatment (SVR-4 actual) outcome, defined as the HCV RNA response outcome within 2–8 weeks after the last day of study drug administration
- Hepatitis C virus (log_{10} IU/mL) change from baseline to Week 24
- Occurrence of documented DAA resistance

3.4.2 Safety Outcome Measures

The safety outcome measures for this study are as follows:

- Incidence and severity of adverse events (AEs) and SAEs and reasons for the discontinuation of any study medication. The Division of AIDS Table for Grading the Severity of Adult and Pediatric Adverse Events (ACTG) guidelines will be used to grade AEs.
- Clinically significant changes in vital signs, laboratory tests, and ECG results from screening/baseline

3.4.3 Pharmacokinetic Outcome Measures

- Trough concentrations for RO4995855, RO5012433, and boceprevir

3.4.4 Exploratory Outcome Measure

The exploratory outcome measure for this study is as follows:

- Interferon gamma-induced protein 10kDa levels will be measured at baseline in all patients.

4. MATERIALS AND METHODS

4.1 PATIENTS

Patients enrolled using protocol version 4 will not be permitted to be re-randomized to this study and enrolled for a second course of treatment. Patients who have been enrolled prior to protocol version 4 and randomized to Group C will be offered mericitabine, in addition to the treatment they are receiving. Maximum treatment durations for each component of a patient's regimen will be as follows:

- Boceprevir: 24 weeks (Arm A) or 48 weeks (Arm B)
- RO5024048: 24 weeks maximum
- Pegasys/Copegus: 24 weeks (Arm A) or 48 weeks (Arm B)

4.1.1 Inclusion Criteria

Patients must meet the following criteria for study entry:

1. Male or female aged 18 years and older
2. Serologic evidence of CHC infection by an anti-HCV antibody (Ab) test (current or historical)
3. Evidence of CHC infection > 6 months duration
4. Serum HCV RNA quantifiable at ≥ 50,000 IU/mL as demonstrated by the Roche COBAS® TaqMan® HCV Test
5. Evidence of HCV genotype 1a or 1b infection by molecular assay
6. The following information related to the patient's response to the previous course of PEG-IFN/RBV therapy must be available in the medical records of the patient: (1) approved doses of prior PEG-IFN/RBV treatment and the start/end date of previous treatment with PEG-IFN/RBV, (2) documentation of previous dose modifications or interruptions (or lack thereof) to ensure documentation of previous compliance with therapy, (3) HCV RNA prior to the start of previous treatment and at 12 weeks after the start of treatment (window of Week 11 to Week 16) showing a null response, defined as a < 2 $\log_{10}$ IU/mL decrease in viral titer after at least 12 weeks of treatment with PEG-IFN/RBV, (4) HCV assay used, and (5) limit of detection of the assay used.
7. Patients must have discontinued prior HCV treatment at least 12 weeks prior to enrollment (receipt of first dose) in this trial.
8. Chronic liver disease consistent with CHC infection as seen via biopsy, using the scoring methods in Appendix B Patients designated as not having cirrhosis or incomplete/transition to cirrhosis must have had a liver biopsy consistent with CHC within 24 calendar months of the first dose. For patients with cirrhosis or incomplete/transition to cirrhosis, there is no time frame for the biopsy. Fibroscan assessment is allowed in such patients but must be performed within 12 calendar months of the first dose. An elasticity score of ≥ 12.5 kPa will be used to designate incomplete/transition to cirrhosis or cirrhosis (i.e., 'cirrhotic'). If an elasticity score of

≥ 9.5 kPa but < 12.5 kPa is returned, the patient must receive a liver biopsy to determine whether his or her case is described as 'cirrhotic' or 'non-cirrhotic'.

9. Patients with cirrhosis or incomplete/transition to cirrhosis must have an abdominal ultrasound, computed tomography (CT) scan, or magnetic resonance imaging (MRI) scan without evidence of hepatocellular carcinoma (within 6 months prior to randomization), an endoscopy without evidence of bleeding gastroesophageal varices (within 2 years prior to randomization), and a serum alpha-fetoprotein (AFP) < 100 ng/mL (< 100 µg/L).
10. Normal cardiac troponin I value at the screening visit (< 0.100 ng/mL)
11. Serum total bilirubin less than the upper limit of normal (ULN) at the screening visit, unless there is a documented history of Gilbert's syndrome
12. Negative serum pregnancy test at screening (for females of childbearing potential). Negative urine pregnancy test documented within the 24-hour period prior to the first dose of study drugs confirmed by a negative serum pregnancy test collected within 24 hours prior to the first dose of study drug
13. Female patients of childbearing potential must agree to use two forms of non-hormonal contraception (i.e., condom, cervical barrier, intrauterine device [IUD], spermicide, or sponge) during treatment and after the last dose of boceprevir, RO5024048, and Copegus (in accordance with the locally approved label for Copegus). All males with female partners of childbearing potential must use two forms of effective contraception (combined) during treatment and following the last dose of Copegus in accordance with the locally approved label for Copegus.
14. Willingness to give written informed consent and willingness to participate in and comply with the study requirements

4.1.2 Exclusion Criteria

Patients who meet any of the following criteria will be excluded from study entry:

1. Infection with any HCV genotype other than genotype 1a or 1b
2. Body mass index < 18 or ≥ 36
3. Positive test at screening for anti–hepatitis A virus (HAV) IgM Ab, hepatitis B surface antigen (HBsAg), or anti-HIV Ab
4. History of having received RO5024048/boceprevir or any cross-resistant DAA agent at any previous time or use of any other systemic antiviral therapy with established or perceived activity against HCV ≤ 3 months prior to the first dose of study drug
5. Herbal agents (e.g., milk thistle) ≤ 1 month prior to the first dose of study drug and throughout the duration of the study
6. History of having received any investigational drug ≤ 3 months prior to the first dose of study drug or the expectation that such drugs will be used during the study. Patients enrolled in this study cannot be simultaneously enrolled in another study for research, diagnostic, or treatment purposes.

7. History or other evidence of a medical condition associated with chronic liver disease other than HCV (e.g., hemochromatosis, autoimmune hepatitis, Wilson's disease, alpha-1 antitrypsin deficiency, alcoholic liver disease, and/or toxin exposure)
8. Females who are pregnant or breastfeeding
9. Males with female partners who are pregnant
10. Absolute neutrophil count (ANC) < 1.5 × 10^3 cells/μL (< 1.5 × 10^9 cells/L)
11. Platelet count < 90 × 10^3 cells/μL (< 90 × 10^9 cells/L)
12. Hemoglobin concentration < 12 g/dL (120 g/L) in females or < 13 g/dL (130 g/L) in males or a baseline increased risk for anemia (e.g., thalassemia, sickle cell anemia, spherocytosis, history of gastrointestinal bleeding) or in those for whom anemia would be medically problematic
13. The use of colony-stimulating factors such as granulocyte colony-stimulating factor (G-CSF), erythropoietin, blood transfusion, or other therapeutic agents to elevate hematology parameters to facilitate patient entry into the study within the last 6 months
14. Any patient with a history of severe psychiatric disease, including psychosis and/or depression, characterized by a suicide attempt, hospitalization for psychiatric disease, or a period of disability as a result of psychiatric disease who does not agree to have a psychiatric evaluation at screening and who does not agree to have continued monitoring by a mental health specialist at least every 4 weeks during the study
15. History of immunologically mediated disease (e.g., vasculitis, cryoglobulinemia, inflammatory bowel disease, idiopathic thrombocytopenic purpura, lupus erythematosus, autoimmune hemolytic anemia, scleroderma, severe psoriasis [defined as affecting > 10% of the body, where the palm of one hand equals 1% or if the hands and feet are affected], rheumatoid arthritis requiring more than intermittent non-steroidal anti-inflammatory medications for management). Patients with a history of celiac disease may enroll in the study.
16. History or other evidence of decompensated liver disease. Coagulopathy, hyperbilirubinemia, hepatic encephalopathy, hypoalbuminemia, ascites, and bleeding from esophageal varices are conditions consistent with decompensated liver disease.
17. Serum creatinine > 1.5 times the ULN
18. History of pre-existing renal disease. Patients with a history of nephrolithiasis will be allowed.
19. Estimated creatinine clearance (CRCL) of ≤ 70 mL/min (≤ 1.17 mL/sec), calculated by the Cockcroft-Gault formula (see Appendix C)
20. Type 1 or 2 diabetes with glycosylated hemoglobin (HbA1c) of ≥ 8.5% at the screening visit

21. History or other evidence of chronic pulmonary disease associated with functional limitation
22. History of severe cardiac disease (e.g., New York Heart Association Functional Class III or IV, myocardial infarction within 6 months, ventricular tachyarrhythmia requiring ongoing treatment, unstable angina, or other significant cardiovascular disease). Patients with stable cardiac disease (e.g., 6 months after bypass surgery, stent replacement, etc.) as confirmed by a cardiologist will be permitted. In addition, patients with documented or presumed coronary artery disease or cerebrovascular disease should not be enrolled if in the judgment of the investigator an acute decrease in Hgb by up to 4 g/dL would not be well tolerated.
23. Patients with higher potential of duration of ventricular depolarization and repolarization (corrected) (QTc) prolongation as defined by duration of ventricular depolarization and repolarization (QT) interval for heart rate using Fridericia's formula (QTcF) > 450 ms (average from multiple readings) or notable resting bradycardia < 50 beats/min or notable resting tachycardia > 100 beats/min or personal or family history of congenital long QT syndrome or sudden death
24. History of uncontrolled severe seizure disorder
25. History of any neoplastic disease within the last 5 years, with the exception of localized or in situ carcinoma of the skin (e.g., basal or squamous cell carcinoma)
26. History of any systemic antineoplastic or immunomodulatory treatment (including supraphysiologic doses of steroids and radiation) ≤ 6 months prior to the first dose of study drug or the expectation that such treatment will be needed at any time during the study
27. Poorly controlled thyroid dysfunction
28. History or other evidence of a clinically relevant ophthalmologic disorder due to diabetes mellitus or hypertension or history or other evidence of severe retinopathy (e.g., cytomegalovirus, macular degeneration)
29. History of major organ transplantation with an existing functional graft
30. History or other evidence of severe illness or any other conditions that would make the patient, in the opinion of the investigator, unsuitable for the study
31. Use of contraindicated concomitant medications that are highly dependent on CYP3A4/5 for clearance, and for which elevated plasma concentrations are associated with serious and/or life threatening events. Potent CYP3A4/5 inducers are also contraindicated (Victrelis™ USPI 2011).
32. Evidence of excessive alcohol, drug, or substance abuse (excluding marijuana use) within 1 year of the first dose
33. History of allergy to RBV, pegylated IFNs, or other IFNs

4.2 METHOD OF TREATMENT ASSIGNMENT AND BLINDING

Approximately 60 patients who qualify for the study will be enrolled. Patients will be randomized in a 1:1 ratio. Randomization will be centralized and stratified by HCV genotype (1a or 1b) and by IL28B genetic polymorphism (CC, CT, or TT).

The study is double-blind, and neither study staff, monitors or patients will know which treatment arm individuals have been randomized to.

The randomization list will not be provided to the study center and to the monitors. Patients who have previously been enrolled in Group C will be unblinded and offered mericitabine, in addition to the treatment they are receiving, for a maximum of 24 weeks.

An IMC and safety project team will be unblinded for routine aggregate safety reviews. For purposes of modeling and simulation, drug resistance monitoring, and PK analysis, select members will be unblinded. In order for the Sponsor to make recommendations or decisions regarding further development of the drug, selected members of the project team will have access to the blinding code for interim analyses.

In order to facilitate analysis of the biologic samples collected in this study, the treatment code will be released—according to procedures in place to ensure the integrity of the data—to the responsible analytical person when the samples have been received at the analytical site and are ready for assay. The result of the analysis must not be released with individual patient identification until the database is closed.

If unblinding is necessary for patient management (in the case of a SAE), the investigator will be able to break the treatment code by contacting the interactive voice response system (IVRS). Treatment codes should not be broken except in emergency situations. If the investigator wishes to know the identity of the study drug for any reason, he or she should contact the Medical Monitor directly. The investigator should document and provide an explanation for any premature unblinding (e.g., accidental unblinding, unblinding due to a SAE).

As per health authority reporting requirements, Roche will break the treatment code for all unexpected SAEs (see Section 5.7) that are considered by the investigator to be related to the study drug.

All other individuals directly involved in this study will remain blinded until all patients have completed all study assessments and the database is cleaned and closed.

4.3 STUDY TREATMENT

4.3.1 Formulation, Packaging, and Handling

RO5024048: 500-mg tablets; store below 30°C (86°F)

Boceprevir: 200-mg capsules; should be refrigerated at 2°C–8°C (36°F–46°F). Boceprevir can also be stored at room temperature for up to 3 months until the expiration date (please refer to local labeling for details on temperature, duration, etc.)

Pegasys: 180 µg in 1-mL solution in vials; refrigerate at 2°C–8°C (36°F–46°F)

Copegus: 200-mg tablets; store below 30°C (86°F)

For further details, see the local prescribing information for boceprevir, Pegasys, and Copegus (see Appendix E).

4.3.2 Dosage, Administration, and Compliance

RO5024048 will be administered at a dose of 1000 mg orally (PO) BID for 24 weeks.

Boceprevir will be administered at a dose of 800 mg PO TID (at recommended intervals of 7–9 hours) for 24 or 48 weeks.

Pegasys will be administered at a dose of 180 µg via subcutaneous SC route QW for 24 or 48 weeks.

Copegus will be administered at a total daily dose of 1000 mg (< 75 kg) or 1200 mg (≥ 75 kg) PO for 24 or 48 weeks.

Because the absorption of RO5024048, boceprevir, and Copegus increases when administered with a meal, all patients should take RO5024048, boceprevir, and Copegus with food. "With food" means the medication should be taken within 30 minutes before or within 1 hour after a meal or light snack.

No dose modifications will be allowed for RO5024048 or boceprevir. Guidelines for dosage modification and treatment interruption or discontinuation are provided in Appendix F.

Compliance will be measured by a patient drug diary. Each patient will be required to record each dose of study medication taken. Patients will be asked to return used and unused bottles/vials along with the drug diary at designated drug dispensing visits (See Appendix A) for drug reconciliation. Missed or reduced doses of study medication along with the reason for the missed or reduced dose must also be recorded and entered on the electronic Case Report Form (eCRF).

4.3.3 Investigational Medicinal Product Accountability

All investigational medicinal products (IMPs) required for completion of this study (RO5024048, boceprevir, Pegasys, Copegus) will be provided by Roche.
The investigational site will acknowledge receipt of IMPs, using the IVRS to confirm the shipment condition and content. Any damaged shipments will be replaced.

IMPs will either be disposed of at the study site according to the study site's institutional standard operating procedure or returned to Roche with the appropriate documentation. The site's method of IMP destruction must be agreed upon by Roche. The site must obtain written authorization from Roche before any IMP is destroyed, and IMP destruction must be documented on the appropriate form.

Accurate records of all IMPs received at, dispensed from, returned to, and disposed of by the study site should be recorded on the Drug Inventory Log.

4.3.4 Posttrial Access

Roche does not intend to provide study medications (RO5024048, boceprevir, Pegasys, or Copegus) or other study interventions to patients after the conclusion of the study or any earlier withdrawal.

Patients with evidence of boceprevir- and/or RO5024048-resistant mutations that persist after treatment completion and follow up, or patients experiencing partial response or viral load breakthrough (confirmed by retest) while on RO5024048 treatment that do not have known boceprevir or RO5024048 resistance mutations are encouraged to enroll in a separate Roche study (NV22688) to determine the durability of the resistant viral mutation.

4.4 CONCOMITANT THERAPY

4.4.1 Permitted Therapy

Concomitant therapy includes any medication (e.g., prescription drugs, over-the-counter drugs, herbal/homeopathic remedies, nutritional supplements) used by a patient from 30 days prior to screening to the study completion/early termination visit. All concomitant medications should be reported to the investigator and recorded on the Concomitant Medications eCRF.

The total daily dose of acetaminophen (paracetamol) should not exceed 4 g/day.

Alcohol consumption is to be strongly discouraged. During the study, patients should not consume more than 20 g of alcohol daily because of possible adverse effects on CHC patients receiving IFNs.

Patients who use hormone replacement therapy or other maintenance therapy should continue its use. Patients who have been on stable hormone replacement therapy for a period of at least 2 months prior to screening will not be excluded from the study.

Please refer to the package inserts for boceprevir, Pegasys, and Copegus for information regarding concomitant medications and potential drug interactions (see Appendix E).

4.4.2 Growth Factor Use

The Sponsor is unable to recommend the off-label use of growth factors, such as erythropoietin or G-CSF in managing HCV patients and can provide no guidance for their use; however, physicians are advised to manage and treat treatment-related AEs according to their best medical judgment.

Recent safety information for erythropoiesis-stimulating agents (ESAs) has indicated that if Hgb is kept too high, the chance of heart attack, stroke, heart failure, blood clots, and death may increase. Growth factors such as erythropoietin to raise RBC counts or G-CSF to raise neutrophil counts have been used successfully to treat patients with cytopenias during combination therapy with RBV and IFN, respectively. Currently, however, there are insufficient data to recommend their routine use as a means to avoid or ameliorate PEG-IFN and RBV dose reductions in clinical practice. The proper role, dose, and side effects of these adjunctive therapies have yet to be defined (Strader et al. 2004; NIH).

The U.S. Food and Drug Administration (FDA) recommends the following guidelines for ESA use:

- Erythropoiesis-stimulating agents should not be initiated until Hgb falls below 10 g/dL (100 g/L).
- Iron studies should be obtained prior to and during treatment with ESAs.
- Iron supplementation should be initiated for deficient patients and to maintain transferrin saturation at a level that will support erythropoiesis.
- Once an ESA is initiated, Hgb levels and blood pressure (BP) must be monitored weekly until Hgb level stabilizes.
- Treatment should target an Hgb level sufficient to avoid transfusion.
- The ESA dose should be titrated to treatment response.
- The ESA dose should be reduced if Hgb rises by more than 1 g/dL (10 g/L) in a 2-week period.
- The ESA dose should not exceed those recommended for currently approved indications.
- Erythropoiesis-stimulating agents should not be used in patients at increased risk of thromboembolic or cardiovascular events, including those with inadequately controlled hypertension and in patients diagnosed with malignancies.

Detailed information on these medical interventions must be properly documented on the AE eCRF page.

4.4.3 Prohibited Therapy

Please refer to Section 4.1.2 and the boceprevir package insert (Victrelis USPI 2011) for the list of excluded therapies, including drugs that are contraindicated with boceprevir.

Systemic antiviral treatments with established or perceived activity against HCV and antineoplastic and immunomodulatory treatments (including steroids at supraphysiologic doses and radiation) are not allowed during the study. Steroids given at physiologic doses (defined as topical/inhaled steroids for an indefinite duration), steroid replacement therapy at doses calculated to mimic normal serum levels for an indefinite duration, and oral short-course pulse therapy equivalent to using up to 30 mg prednisone once daily [QD] for 7 days) are permitted. Other remedies being taken by the patient for possible or perceived effects against HCV, such as herbal agents, are excluded, as are other investigational drugs.

4.5 STUDY ASSESSMENTS

4.5.1 Description of Study Assessments

Patients will be assessed according to the Schedule of Assessments (see Appendix A). Baseline assessments must be obtained on the first day of test drug administration prior to the initiation of any study medication.

4.5.1.1 Efficacy Assessments

Serum HCV RNA will be assessed in all randomized patients by polymerase chain reaction (PCR) techniques using the Roche COBAS TaqMan HCV v2.0 Test (less than the LLOQ; < 25 IU/mL).

4.5.1.2 Safety Assessments

In addition to the safety measurements detailed in the Schedule of Assessments (see Appendix A), documentation will be collected for dose adjustments, interruptions, and premature withdrawals (PWs) from the study (discontinuation of all four study drugs) for any reason.

4.5.1.3 Laboratory Assessments

Study-associated laboratory measurements will be performed centrally according to the Schedule of Assessments (see Appendix A).

Reference ranges for the local study laboratory parameters must be supplied to Roche before the study starts.

Additional serum samples will be collected during the course of this study. These samples may be used to perform or repeat laboratory tests outlined in the Schedule of Assessments. They also may be used to investigate questions regarding disease-related or host-related factors not specified in this protocol. Examples include viral quantification and sequencing and host response factors to infection and treatment.

Protection of patient confidentiality will extend to any data generated from the assaying of these samples.

The procedures for the collection, handling, and shipping of laboratory samples are specified in the Laboratory Manual(s) provided to each study site.

The samples for this study should be classified, packed, and shipped as UN 3373 Biological Substance, Category B.

4.5.1.4 Electrocardiograms

All ECGs are to be obtained predose and prior to a meal or laboratory/PK sampling collection if these procedures are scheduled at the same time. All patients will have 12-lead ECGs performed in a resting supine position, in triplicate (within 10 minutes), according to the Schedule of Assessments (see Appendix A).

All ECGs must be performed using a standard high-quality, high-fidelity electrocardiograph machine equipped with computer-based interval measurements and provided by the Sponsor through a central supplier. Electrocardiogram recordings will be transmitted to the central ECG supplier where the same cardiologist will evaluate all ECG recordings for any given patient.

4.5.1.5 Pharmacokinetic Assessments

Before the morning dose of all study drugs on Day 1 (baseline) and at Week 8, PK plasma and serum samples will be collected from all patients to measure RO4995855, its metabolite (RO5012433), boceprevir, and Copegus (samples for Copegus will be analyzed only if needed). A predose PK serum sample will be collected from all patients and stored for future analysis of Pegasys if needed. The total amount of blood required is approximately 16 mL per patient.

Should an SAE occur during the first 48 weeks of therapy—requiring discontinuation of RO5024048 and/or boceprevir—the site should make every effort to obtain a plasma and serum blood sample for PK analysis as soon as possible to determine whether there is a correlation between the event and the concentration of study drug(s). The same blood samples may also be used for PK assessment of other concomitant medications if needed.

The procedures for the collection, handling, storage, and shipping of the plasma and serum samples for the PK analysis are specified in a supplemental laboratory manual. Actual sampling times will be recorded for each PK sample. The previous dosing time for all study drugs prior to the PK sample collection should be recorded on the eCRF.

Plasma concentrations for RO4995855, RO5012433, boceprevir, and Copegus will be measured by specific and validated liquid chromatography tandem mass spectrometry (LC/MS/MS) methods. Serum concentrations for Pegasys will be measured by specific and validated ELISA methods.

4.5.1.6 Pharmacodynamic Assessments

Antiviral activity of the study drugs will be assessed at each visit by the quantification of serum HCV RNA viral concentration.

4.5.1.7 Exploratory Biomarker Assessments

Baseline plasma samples will be collected as detailed in the Schedule of Assessments (see Appendix A) to quantify the concentration of IP-10/CXCL10, an IFN-inducible protein, using a research grade assay. The possibility of baseline IP-10 levels affecting response to treatment will be evaluated. Results will not be included in the Clinical Study Report. Details on sampling procedures, sample storage, and shipment are specified in the Laboratory Service Manual.

4.5.1.8 Hepatitis C Virus Drug-Resistance Monitoring

Blood samples will be collected throughout the study as detailed in the Schedule of Assessments (see Appendix A) to monitor for the development of drug resistance.

During the course of the study, phenotypic and/or sequence analyses will be performed on samples from patients who experience virologic breakthrough, partial response, non-response, relapse, and phenotypic analysis at baseline for approximately 5% of patients who maintain response through follow up. To prevent prolonged dosing in the event of a virologic breakthrough or partial response after one single measurement of HCV RNA breakthrough, a confirmatory sample should be drawn within 2 weeks. To confirm a relapse after one single measurement of HCV RNA relapse, another sample should be drawn within 2 weeks.

Patients who develop resistance to RO5024048 and/or to boceprevir while on RO5024048 and boceprevir treatment will be monitored for the persistence of RO5024048- and/or boceprevir-resistant mutation(s) during the follow-up period.

Population sequencing of the complete coding sequence of the HCV NS3/4A and/or NS3 protease and NS5B (for boceprevir- and RO5024048-containing cohorts) of all baseline samples (Day 1) will be performed using standard sequencing technology.

Patients with evidence of a partial response or virologic breakthrough (in the presence or absence of confirmed resistance) will be asked to participate in a separate Roche study for resistance monitoring (Study NV22688), where sequence analysis will be performed. For these patients, samples will be collected and analyzed as detailed in the protocol. For those patients where resistance is not confirmed, samples will be taken for future analyses in case a new resistance mutation to RO5024048 is identified. Clonal and/or ultradeep sequencing and/or phenotypic analysis may also be performed on these samples.

Patient Identification for Resistance Analysis

Samples will be selected for sequence determination of the NS3 protease and NS5B coding region(s) from patients experiencing (for the purposes of sequence determination an HCV RNA ≥ 1000 IU/mL is required):

1. Virologic breakthrough
   a) Those patients who show either a sustained increase in HCV RNA of ≥ 1 $\log_{10}$ IU/mL (≥ 2 consecutive measurements) while on boceprevir/RO5024048 compared with the on-treatment nadir (where the nadir was a ≥ 1 $\log_{10}$ IU/mL decrease from baseline after 2 weeks of treatment
   b) Those patients with confirmed quantifiable HCV RNA (≥ 25 IU/mL by ≥ 2 consecutive measurements) in a patient who had been previously confirmed as having unquantifiable HCV RNA (< 25 IU/mL by ≥ 2 consecutive measurements) before the end of boceprevir/RO5024048 treatment
   c) Those patients who responded (< 25 IU/mL by ≥ 2 consecutive measurements) to boceprevir/RO5024048 treatment and who have subsequent quantifiable viral load (≥ 25 IU/mL by ≥ 2 consecutive measurements) once boceprevir/RO5024048 treatment has been completed (but Pegasys/Copegus treatment continues).
2. Partial response. Those patients who show an initial HCV RNA decline (a ≥ 1 $\log_{10}$ IU/mL decrease from baseline after 2 weeks of treatment) followed by stabilization (≥ 2 consecutive measurements similar to those at nadir) while on boceprevir/RO5024048 treatment and/or experience a HCV RNA ≥ 1000 IU/mL by the end of boceprevir/RO5024048 treatment of at least 4 weeks in duration
3. Non-response. A decrease in HCV RNA of < 1 $\log_{10}$ IU/mL from baseline after 2 weeks of treatment
4. Relapse. Patients who responded to boceprevir/RO5024048 treatment and have subsequent quantifiable viral load (≥ 25 IU/mL by ≥ 2 consecutive measurements) once all treatment has been completed.

Sample Selection, Clonal Sequence, and Phenotypic Analyses

The following samples from patients identified in Section 4.5.1.8 will be selected for sequence determination of the NS3 protease and/or NS5B coding sequences (if HCV RNA ≥ 1000 IU/mL).

1. Baseline sample (Day 1 before initiation of treatment) for patients with HCV RNA breakthrough, partial response, non-response, and relapse
2. First sample after nadir that shows HCV RNA > 1000 IU/mL for patients who experience HCV RNA breakthrough while on boceprevir/RO5024048 treatment (or subsequent samples if amplification fails)

3. Additional samples collected after breakthrough, but before the end of boceprevir/RO5024048 treatment may be assessed, particularly if viral complementary DNA (cDNA) amplification from the first sample after nadir failed for patients with HCV RNA breakthrough
4. End-of-boceprevir/RO5024048 treatment sample from patients who experience partial response while on boceprevir/RO5024048 treatment
5. First sample after quantifiable viral load (or later samples if HCV RNA is higher and allows amplification) that shows HCV RNA > 1000 IU/mL for patients who experience sustained HCV RNA relapse once study drug treatment has been completed

Clonal Sequence Analyses

Clonal sequence analysis will be performed if a population-based sequence of on-treatment samples (or PK data) cannot explain an HCV RNA breakthrough, partial response, or non-response and will be performed on the on-treatment and baseline samples previously analyzed through population sequence.

Clonal sequence analysis will be performed on on-treatment samples from patients with mixtures of amino acid substitutions at positions known to confer resistance to boceprevir or RO5024048 that develop during therapy. This analysis will assist in the understanding of the percentage of mutant/wild-type variants within a patient's quasispecies.

For patients who experienced virologic failure clearly associated with the emergence of a boceprevir and/or RO5024048 resistance–associated substitution(s) and for the analysis of the last sample for resistance testing in Study NV27780 (to assess the persistence of the resistance mutation[s]), population sequence will be used as the primary methodology for the detection of a previously detected resistance mutation(s). Should such mutation(s) not be detected by population sequence, a clonal-based method (≥ 80 clones/sample or equivalent) will be used to assess the patient's eligibility for enrollment in Study NV22688.

Sequence variations of the samples after HCV RNA breakthrough or partial response will be determined and compared with the baseline sequences of the samples from each selected patient and with the corresponding subtype reference sequence.

Phenotypic Characterization

Phenotypic characterization of samples identified for resistance analysis, as outlined in Section 4.5.1.8, will be carried out by the amplification of the entire NS3 protease and/or NS5B coding region (if HCV RNA is > 1000 IU/mL) and cloning into the appropriate replicon shuttle vector. Attempts will be made to carry out phenotypic analysis of the following samples:

1. Baseline sample (before initiation of study drug treatment) for patients with virologic breakthrough, partial response, non-response, and relapse

2. First sample after nadir that shows HCV RNA > 1000 IU/mL for patients who experience HCV RNA breakthrough while on study drug treatment (or subsequent samples if amplification fails)
3. Additional samples collected after virologic breakthrough but before the end of treatment may be assessed, particularly if viral cDNA amplification from the first sample after nadir fails.
4. End-of-study drug treatment sample from patients who experience partial response while on study drug treatment
5. First sample after breakthrough that shows HCV RNA > 1000 IU/mL for patients who experience sustained HCV RNA breakthrough once boceprevir/RO5024048 treatment has been completed
6. First sample after relapse that shows a HCV RNA > 1000 IU/mL for patients who experience sustained HCV RNA relapse once all treatment has been completed

The shuttle vectors will be transfected into Huh-7 cells, and the phenotype against boceprevir and RO4995855 and other approved HCV antivirals will be determined after 3 days of incubation with inhibitor(s).

Specific NS3 protease or NS5B amino acid sequence variations observed in samples of treated patients who experience viral breakthrough, partial response, rebound, or non-response will be evaluated using a laboratory reference strain replicon through site-directed mutagenesis as follows:

- All observed amino acid changes observed in conserved-site substitutions ($n \geq 2$)
- All polymorphic-site substitutions changing to a specific amino acid residue not identified as a polymorphism ($n \geq 2$)

The inhibitory potency of boceprevir or RO4995855 (and other approved antivirals) will be determined using the HCV replicon site-directed mutants generated as outlined above and compared with the inhibitory potency of boceprevir or RO5024048 using the wild-type HCV replicon. The quotient of the half maximum effective concentration (EC_{50}) values determined in the mutant and the wild-type replicons will be reported as fold change (FC) values to describe the level of change in susceptibility to boceprevir or RO5024048 conferred by individual NS3/4A or NS5B amino acid substitution(s).

Phenotypic studies may also include analysis of samples derived from patients having a virologic response. Samples from selected patients (encompassing the NS3 protease region or NS5B) will be processed to determine the sensitivity toward the inhibition of boceprevir or RO4995855 and to IFN alfa-2a. In vitro assessment of cross-resistance to other HCV inhibitors and further HCV sequence analyses may be performed on selected samples and will require amplification and subcloning of sequences from the HCV genome.

Sampling procedures, storage conditions, and shipment instructions will be provided in the Laboratory Service Manual provided to each study site.

A total of 12 mL per sample will be required for resistance monitoring.

4.5.1.9 Samples for Roche Clinical Repository

Overview of the Roche Clinical Repository

The Roche Clinical Repository (RCR) is a centrally administered group of facilities for the long-term storage of human biologic specimens, including body fluids, solid tissues, and derivatives thereof (e.g., DNA, RNA, proteins, peptides). The collection and analysis of RCR specimens will facilitate the rational design of new pharmaceutical agents and the development of diagnostic tests, which may allow for individualized drug therapy for patients in the future.

Specimens for the RCR will be collected from patients who give specific consent to participate in this optional research. RCR specimens will be used to achieve the following objectives:

- To study the association of biomarkers with efficacy, AEs, or other effects associated with medicinal products
- To increase knowledge and understanding of disease biology
- To study drug response, including drug effects and the processes of drug absorption and disposition
- To develop biomarker or diagnostic assays and establish the performance characteristics of these assays

Approval by the Institutional Review Board or Ethics Committee

Sampling for the RCR is contingent upon the review and approval of the exploratory research and the RCR portion of the Informed Consent Form by each site's Institutional Review Board or Ethics Committee (IRB/EC) and, if applicable, an appropriate regulatory body. If a site is not granted approval for RCR sampling, this section of the protocol will not be applicable at that site.

Sample Collection

Optional plasma, RNA, and DNA RCR samples will be collected according to the Schedule of Assessments (see Appendix A).

For all samples, dates of consent and specimen collection should be recorded on the associated RCR page of the eCRF. For sampling procedures, storage conditions, and shipment instructions, see the Sample Handling and Logistics Manual.

Roche Clinical Repository specimens will be destroyed no later than 15 years after the date of final closure of the associated clinical database. The RCR storage period will be in accordance with the IRB/EC-approved Informed Consent Form and applicable laws (e.g., health authority requirements).

The dynamic biomarker specimens will be subject to the confidentiality standards described in Section 8.4. The genetic biomarker specimens will undergo additional processes to ensure confidentiality, as described below.

Confidentiality

Given the sensitive nature of genetic data, Roche has implemented additional processes to ensure patient confidentiality for RCR specimens. Upon receipt by the RCR, each specimen is "double-coded" by replacing the patient identification number with a new independent number. Data generated from the use of these specimens and all clinical data transferred from the clinical database that are considered relevant are also labeled with this same independent number. A "linking key" between the patient identification number and this new independent number is stored in a secure database system. Access to the linking key is restricted to authorized individuals and is monitored by audit trail. Legitimate operational reasons for accessing the linking key are documented in a standard operating procedure. Access to the linking key for any other reason requires written approval from the Pharma Repository Governance Committee and Roche's Legal Department, as applicable.

Data generated from RCR specimens must be available for inspection upon request by representatives of national and local health authorities, and Roche monitors, representatives, and collaborators, as appropriate.

Patient medical information associated with RCR specimens is confidential and may only be disclosed to third parties as permitted by the Informed Consent Form (or separate authorization for use and disclosure of personal health information) signed by the patient, unless permitted or required by law.

Data derived from RCR specimen analysis on individual patients will generally not be provided to study investigators unless a request for research use is granted. The aggregate results of any research conducted using RCR specimens will be available in accordance with the effective Roche policy on study data publication.

Any inventions and resulting patents, improvements, and/or know-how originating from the use of RCR data will become and remain the exclusive and unburdened property of Roche, except where agreed otherwise.

Consent to Participate in the RCR

The Informed Consent Form will contain a separate section that addresses participation in the RCR. The investigator or authorized designee will explain to each patient the objectives, methods, and potential hazards of participation in the RCR. Patients will be told that they are free to refuse to participate and may withdraw their specimens at any time and for any reason during the storage period. A separate, specific signature will be required to document a patient's agreement to provide optional RCR specimens.

Patients who decline to participate will check a “no” box in the appropriate section and will not provide a separate signature.

The investigator should document whether or not the patient has given consent to participate by completing the RCR Research Sample Informed Consent eCRF.

In the event of an RCR participant's death or loss of competence, the participant's specimens and data will continue to be used as part of the RCR research.

Withdrawal from the RCR

Patients who give consent to provide RCR specimens have the right to withdraw their specimens from the RCR at any time for any reason. If a patient wishes to withdraw consent to the testing of his or her specimens, the investigator must inform the Medical Monitor in writing of the patient's wishes using the RCR Subject Withdrawal Form and, if the trial is ongoing, must enter the date of withdrawal on the RCR Research Sample Withdrawal of Informed Consent eCRF. The patient will be provided with instructions on how to withdraw consent after the trial is closed. A patient's withdrawal from Study NV27780 does not, by itself, constitute withdrawal of specimens from the RCR. Likewise, a patient's withdrawal from the RCR does not constitute withdrawal from Study NV27780.

Monitoring and Oversight

RCR specimens will be tracked in a manner consistent with Good Clinical Practice by a quality-controlled, auditable, and appropriately validated laboratory information management system to ensure compliance with data confidentiality as well as adherence to authorized use of specimens as specified in this protocol and in the Informed Consent Form. Roche monitors and auditors will have direct access to appropriate parts of records relating to patient participation in the RCR for the purposes of verifying the data provided to Roche. The site will permit monitoring, audits, IRB/EC review, and health authority inspections by providing direct access to source data and documents related to the RCR samples.

In addition to an internal review body, an independent Science and Ethics Advisory Group—consisting of experts in the fields of biology, ethics, sociology, and law—will advise Roche regarding the use of RCR specimens and on the scientific and ethical aspects of handling genetic information.

4.5.2 Timing of Study Assessments

4.5.2.1 Screening and Pretreatment Assessments

Written informed consent for participation in the study must be obtained before performing any study-specific screening tests or evaluations. Informed Consent Forms for enrolled patients and for patients who are not subsequently enrolled will be maintained at the study site.

Screening evaluations will be performed between 8 weeks before and 1 day before the patient is randomized. All screening evaluations must be completed and reviewed to confirm that patients meet all eligibility criteria before randomization. The investigator will maintain a screening log to record details of all patients screened and to confirm eligibility or record reasons for screening failure, as applicable.

See Appendix A for the schedule of screening and pretreatment assessments.

4.5.2.2 Assessments During Treatment

See Appendix A for the Schedule of Assessments performed during the treatment period.

4.5.2.3 Assessments at Study Completion/Early Termination Visit

Any patient who prematurely discontinues all study drugs should return for the PW Visit as soon as possible after the last dose of study medication. Additional requirements exist for patients who have unquantifiable HCV RNA levels at the time of premature study drug discontinuation (see Appendix A).

- If a patient stops Pegasys, all study medications must be discontinued.
- If a patient stops one DAA (either RO5024048 or boceprevir), the patient may remain on the other DAA (either RO5024048 or boceprevir) plus Pegasys/Copegus.
- If both RO5024048 and boceprevir are stopped, the patient may remain on Pegasys/Copegus.
- If a patient stops Copegus, all study medications must be discontinued.
- Patients should not be treated with a single DAA (either RO5024048 or boceprevir) and Pegasys or with Pegasys monotherapy.
- In other circumstances, the investigator should consult with the Sponsor.

See Appendix A for the Schedule of Assessments performed at the Study Completion/Early Termination Visit.

4.5.2.4 Follow-Up Assessments

After the Study Completion/Early Termination Visit, AEs should be followed as outlined in Section 5.5 and 5.6.

See Appendix A for the schedule of follow-up assessments.

4.6 PATIENT, STUDY, AND SITE DISCONTINUATION

4.6.1 Patient Discontinuation

The investigator has the right to discontinue a patient from any study drug or withdraw a patient from the study at any time. In addition, patients have the right to voluntarily discontinue study drugs or withdraw from the study at any time for any reason. Reasons for discontinuation of a study drug or withdrawal from the study may include, but are not limited to, the following:

- Patient withdrawal of consent at any time
- Any medical condition that the investigator or Sponsor determines may jeopardize the patient's safety if he or she continues in the study
- The investigator or Sponsor determines it is in the best interest of the patient.

4.6.1.1 Discontinuation from Study Drug

All study medications should be stopped for individual patients for any of the following reasons:

- Hepatitis C virus RNA titer > 100 IU/mL by Week 24
- Quantifiable HCV RNA at Week 24
- Hepatitis C virus RNA virologic breakthrough before the end of treatment with boceprevir and/or RO5024048/placebo and/or Pegasys/Copegus. Virologic breakthrough is defined as:

  A sustained increase in HCV RNA of ≥ 1 $\log_{10}$ IU/mL (≥ 2 consecutive measurements) while on boceprevir/RO5024048 compared with the on-treatment nadir (where the nadir is a ≥ 1 $\log_{10}$ IU/mL decrease from baseline after 2 weeks of treatment or ≥ 2 $\log_{10}$ IU/mL decrease from baseline after 4 weeks of treatment)

  Confirmed quantifiable HCV RNA (≥ 25 IU/mL by ≥ 2 consecutive measurements) in a patient who had been previously confirmed as having unquantifiable HCV RNA (< 25 IU/mL by ≥ 2 consecutive measurements) before the end of treatment with boceprevir/RO5024048

  Response (< 25 IU/mL by ≥ 2 consecutive measurements) to boceprevir/RO5024048 treatment and subsequent quantifiable viral load (≥ 25 IU/mL by ≥ 2 consecutive measurements) once boceprevir/RO5024048 treatment has been completed (but Pegasys/Copegus treatment continues)

- Hepatitis C virus partial response before the end of treatment with boceprevir/RO5024048/Pegasys/Copegus/placebo, defined as an initial HCV RNA decline (a ≥ 1 $\log_{10}$ IU/mL decrease from baseline after 2 weeks of treatment or ≥ 2 $\log_{10}$ IU/mL decrease from baseline after 4 weeks of treatment) followed by stabilization (≥ 2 consecutive measurements similar to those at nadir) while on boceprevir/RO5024048/Pegasys/Copegus/placebo treatment and/or a HCV RNA ≥ 1000 IU/mL by the end of boceprevir/RO5024048/Pegasys/Copegus/placebo treatment of at least 4 weeks in duration
- Any confirmed Grade 4 laboratory abnormality deemed clinically significant (based on the investigator's assessment)
- A positive troponin value (> 0.100 ng/mL)

RO5024048 should be stopped for individual patients for any of the following reasons:

- A creatinine clearance decrease ≥ 35% from baseline or < 60 mL/min (< 1.00 mL/sec) confirmed by repeat test, preferably within 1 week
- A urine protein/creatinine ratio ≥ 0.5 (56.51 mg/mmol) and greater than the baseline value confirmed by repeat test, preferably within 1 week (see Appendix H)

Refer to Appendix F and G for more details on stopping rules and for additional guidance on the management of specific AEs and laboratory abnormalities.

Patients who discontinue study drug prematurely will be asked to return to the clinic for a Study Completion/Early Termination Visit (see Section 4.5.2.3) and may undergo follow-up assessments (see Section 4.5.2.4). The primary reason for premature study drug discontinuation should be documented on the appropriate eCRF. Patients who discontinue study drug prematurely will not be replaced.

4.6.1.2 Withdrawal from Study

Every effort should be made to obtain information on patients who withdraw from the study. The primary reason for withdrawal from the study should be documented on the appropriate eCRF. Patients will not be followed for any reason after consent has been withdrawn. Patients who withdraw from the study will not be replaced.

4.6.2 Study and Site Discontinuation

The Sponsor has the right to terminate this study at any time. Reasons for terminating the study may include, but are not limited to, the following:

- The incidence or severity of AEs in this or other studies indicates a potential health hazard to patients.
- Previously unknown data become available that raise significant concerns about the potential risk from participation in the study.
- Patient enrollment is unsatisfactory.

The Sponsor will notify the investigator if the study is placed on hold or if the Sponsor decides to discontinue the study or development program.

The Sponsor has the right to replace a site at any time. Reasons for replacing a site may include, but are not limited to, the following:

- Excessively slow recruitment
- Poor protocol adherence
- Inaccurate or incomplete data recording
- Non-compliance with the International Conference on Harmonisation (ICH) guideline for Good Clinical Practice

5. ASSESSMENT OF SAFETY

5.1 SAFETY PLAN

Adverse events found during nonclinical studies of RO5024048 include glomerular microangiopathy with or without secondary hypertrophic/dilatory changes in the heart and a decrease in red blood cell parameters. A potential class effect of nucleoside analogs is mitochondrial toxicity.

Clinical trials have identified anemia and neutropenia as significant AEs associated with boceprevir (Victrelis USPI 2011). Pegasys/Copegus are associated with well-documented AEs such as psychiatric events; teratogenicity (with Copegus); cytopenias (including hemolytic anemia); flu-like symptoms; infections; and cardiovascular, thyroid, metabolic, autoimmune, respiratory, ophthalmic, and other conditions (Pegasys/Copegus USPI 2011).

Exclusion criteria in this study aim to exclude patients who are at risk for increased toxicity—as determined by the potential and identified AEs of RO5024048, boceprevir, and Pegasys/Copegus—such as patients with significant renal impairment, significant anemia, severe psychiatric disease, etc.

5.1.1 Management of Specific AEs

Regular monitoring of AEs, laboratory parameters, vital signs, and ECGs will ensure that any AEs (e.g., cytopenias) are detected early and treated promptly. Algorithms for the management of specific AEs/laboratory abnormalities (e.g., anemia, renal parameters) are provided in Appendix G and in the boceprevir, Pegasys, and Copegus package inserts (see Appendix E).

Dose-modification rules for this study are outlined in Appendix F. The IMC will review the clinical trial data at regular intervals and at prespecified interim analyses.

5.2 SAFETY PARAMETERS AND DEFINITIONS

Safety assessments will consist of monitoring and recording AEs, including SAEs and non-serious adverse events of special interest; measurement of protocol-specified safety laboratory assessments; measurement of protocol-specified vital signs; and other protocol-specified tests that are deemed critical to the safety evaluation of the study.

Certain types of events require immediate reporting to the Sponsor, as outlined in Section 5.4.

5.2.1 Adverse Events

According to the ICH guideline for Good Clinical Practice, an AE is any untoward medical occurrence in a clinical investigation patient given a pharmaceutical product, regardless of causal attribution. An AE can therefore be any of the following:

- Any unfavorable and unintended sign (including an abnormal laboratory finding), symptom, or disease temporally associated with the use of a medicinal product, whether or not considered related to the medicinal product
- Any new disease or exacerbation of an existing disease (a worsening in the character, frequency, or severity of a known condition)
- Recurrence of an intermittent medical condition (e.g., headache) not present at baseline
- Any deterioration in a laboratory value or other clinical test (e.g., ECG, X-ray) that is associated with symptoms or leads to a change in study treatment or concomitant treatment or discontinuation from study drug
- Adverse events that are related to a protocol-mandated intervention, including those that occur prior to assignment of study treatment (e.g., screening invasive procedures such as biopsies)

5.2.2 Serious Adverse Events (Immediately Reportable to Roche)

An SAE is any AE that meets any of the following criteria:

- Fatal (i.e., the AE actually causes or leads to death)
- Life threatening (i.e., the AE, in the view of the investigator, places the patient at immediate risk of death). This does not include any AE that had it occurred in a more severe form or was allowed to continue might have caused death.
- Requires or prolongs inpatient hospitalization (see Section 5.3.5.10)
- Results in persistent or significant disability/incapacity (i.e., the AE results in substantial disruption of the patient's ability to conduct normal life functions)
- Congenital anomaly/birth defect in a neonate/infant born to a mother exposed to study drug
- Significant medical event in the investigator's judgment (e.g., may jeopardize the patient or may require medical/surgical intervention to prevent one of the outcomes listed above)

The terms “severe” and “serious” are not synonymous. Severity refers to the intensity of an AE (rated as mild, moderate, or severe, or according to ACTG criteria; see Section 5.3.3); the event itself may be of relatively minor medical significance (such as severe headache without any further findings).

Severity and seriousness need to be independently assessed for each AE recorded on the eCRF.

Serious adverse events must be reported by the investigator to the Sponsor immediately (i.e., no more than 24 hours) after learning of the event (see Section 5.4.2 for reporting instructions).

5.3 METHODS AND TIMING FOR CAPTURING AND ASSESSING SAFETY PARAMETERS

The investigator is responsible for ensuring that all AEs (see Section 5.2.1 for definition) are recorded on the AE eCRF and reported to the Sponsor in accordance with instructions provided in this section and in Section 5.4 – 5.6.

For each AE recorded on the AE eCRF, the investigator will make an assessment of seriousness (see Section 5.2.2 for seriousness criteria), severity (see Section 5.3.3), and causality (see Section 5.3.4).

5.3.1 Adverse Event Reporting Period

Investigators will seek information on AEs at each patient contact. All AEs, whether reported by the patient or noted by study personnel, will be recorded in the patient’s medical record and on the AE eCRF.

After informed consent has been obtained **but prior to initiation of study drug**, only SAEs caused by a protocol-mandated intervention should be reported (e.g., SAEs related to invasive procedures such as biopsies).

After initiation of study drug, all AEs, regardless of relationship to study drug, will be reported after the last dose of study drug according to Appendix A. After this period, investigators should report any deaths, SAEs, or other AEs of concern that are believed to be related to prior treatment with study drug (see Section 5.6).

5.3.2 Eliciting AE Information

A consistent methodology of non-directive questioning should be adopted for eliciting AE information at all patient evaluation timepoints. Examples of non-directive questions include the following:

“How have you felt since your last clinic visit?”

“Have you had any new or changed health problems since you were last here?”

5.3.3 Assessment of Severity of AEs

Table 2 provides guidance for assessing AE severity.

Table 2 AE Severity Grading Scale

Grade	Severity	Alternate Description [a]
1	Mild (apply event-specific ACTG grading criteria)	Symptoms causing no or minimal interference with usual social and functional activities
2	Moderate (apply event-specific ACTG grading criteria)	Symptoms causing greater than minimal interference with usual social and functional activities
3	Severe (apply event-specific ACTG grading criteria)	Symptoms causing inability to perform usual social and functional activities
4	Potentially life threatening (apply event-specific ACTG grading criteria)	Symptoms causing inability to perform basic self-care functions OR medical or operative intervention indicated to prevent permanent impairment, persistent disability, or death

ACTG = The Division of AIDS Table for Grading the Severity of Adult and Pediatric Adverse Events; AE, adverse event.

Note: Regardless of severity, some events may also meet regulatory serious criteria. Refer to definitions of a serious adverse event (see Section 5.2.2).

[a] Use these alternate definitions for Grades 1, 2, 3, and 4 events when the observed or reported AE is in the ACTG listing. ACTG v1.0 can be found in Appendix I.

5.3.4 Assessment of Causality of AEs

Investigators should use their knowledge of the patient, the circumstances surrounding the event, and an evaluation of any potential alternative causes to determine whether or not an AE is considered to be related to the study drug, indicating “yes” or “no” accordingly. The following guidance should be taken into consideration:

- Temporal relationship of event onset to the initiation of study drug
- Course of the event, considering especially the effects of dose reduction, discontinuation of study drug, or re-introduction of study drug (where applicable)
- Known association of the event with the study drug or with similar treatments
- Known association of the event with the disease under study
- Presence of risk factors in the patient or use of concomitant medications known to increase the occurrence of the event
- Presence of non-treatment-related factors that are known to be associated with the occurrence of the event

For patients receiving combination therapy, causality will be assessed individually for each protocol-mandated therapy.

5.3.5 Procedures for Recording AEs

Investigators should use correct medical terminology/concepts when recording AEs on the AE eCRF. Avoid colloquialisms and abbreviations.

Only one AE term should be recorded in the event field on the AE eCRF.

5.3.5.1 Diagnosis versus Signs and Symptoms

Injection-Related Reactions

Adverse events that occur during or within 24 hours after study drug injection should be captured as individual signs and symptoms rather than as a diagnosis of allergic reaction or injection reaction.

Other AEs

For AEs other than injection-related reactions, a diagnosis (if known) should be recorded on the AE eCRF rather than individual signs and symptoms (e.g., record only liver failure or hepatitis rather than jaundice, asterixis, and elevated transaminases). However, if a constellation of signs and/or symptoms cannot be medically characterized as a single diagnosis or syndrome at the time of reporting, each individual event should be recorded on the AE eCRF. If a diagnosis is subsequently established, all previously reported AEs based on signs and symptoms should be nullified and replaced by one AE report based on the single diagnosis, with a starting date that corresponds to the starting date of the first symptom of the eventual diagnosis.

5.3.5.2 Adverse Events Occurring Secondary to Other Events

In general, AEs occurring secondary to other events (e.g., cascade events or clinical sequelae) should be identified by their primary cause, with the exception of severe or serious secondary events. However, medically significant AEs occurring secondary to an initiating event that are separated in time should be recorded as independent events on the AE eCRF. For example:

- If vomiting results in mild dehydration with no additional treatment in a healthy adult, only vomiting should be reported on the eCRF.
- If vomiting results in severe dehydration, both events should be reported separately on the eCRF.
- If a severe gastrointestinal hemorrhage leads to renal failure, both events should be reported separately on the eCRF.
- If dizziness leads to a fall and subsequent fracture, all three events should be reported separately on the eCRF.
- If neutropenia is accompanied by infection, both events should be reported separately on the eCRF.

All AEs should be recorded separately on the AE eCRF if it is unclear as to whether the events are associated.

5.3.5.3 Persistent or Recurrent AEs

A persistent AE is one that extends continuously, without resolution, between patient evaluation timepoints. Such events should only be recorded once on the AE eCRF. The initial severity of the event should be recorded, and the severity should be updated to reflect the most extreme severity any time the event worsens. If the event becomes serious, the AE eCRF should be updated to reflect this.

A recurrent AE is one that resolves between patient evaluation timepoints and subsequently recurs. Each recurrence of an AE should be recorded separately on the AE eCRF.

5.3.5.4 Abnormal Laboratory Values

Not every laboratory abnormality qualifies as an AE. A laboratory test result should be reported as an AE if it meets any of the following criteria:

- Accompanied by clinical symptoms
- Results in a change in study treatment (e.g., dosage modification, treatment interruption, or treatment discontinuation)
- Results in a medical intervention (e.g., potassium supplementation for hypokalemia) or a change in concomitant therapy
- Requires further diagnostic evaluation (e.g., an abnormal laboratory result requires a scan/radiologic work up, biopsy, etc., to further manage the case for diagnostic purposes)
- Clinically significant in the investigator’s judgment

It is the investigator’s responsibility to review all laboratory findings. Medical and scientific judgment should be exercised in deciding whether an isolated laboratory abnormality should be classified as an AE.

If a clinically significant laboratory abnormality is a sign of a disease or syndrome (e.g., alkaline phosphatase and bilirubin 5 times the ULN associated with cholecystitis), only the diagnosis (i.e., cholecystitis) should be recorded on the AE eCRF.

If a clinically significant laboratory abnormality is not a sign of a disease or syndrome, the abnormality itself should be recorded on the AE eCRF, along with a descriptor indicating if the test result is above or below the normal range (e.g., “elevated potassium,” as opposed to “abnormal potassium”). If the laboratory abnormality can be characterized by a precise clinical term per standard definitions, the clinical term should be recorded as the AE. For example, an elevated serum potassium level of 7.0 mEq/L should be recorded as “hyperkalemia.”

Observations of the same clinically significant laboratory abnormality from visit to visit should not be repeatedly recorded on the AE eCRF, unless the etiology changes. The initial severity of the event should be recorded, and the severity or seriousness should be updated any time the event worsens.

5.3.5.5 Abnormal Vital Sign Values

Not every vital sign abnormality qualifies as an AE. A vital sign result should be reported as an AE if it meets any of the following criteria:

- Accompanied by clinical symptoms
- Results in a change in study treatment (e.g., dosage modification, treatment interruption, or treatment discontinuation)
- Results in a medical intervention or a change in concomitant therapy
- Clinically significant in the investigator's judgment

It is the investigator's responsibility to review all vital sign findings. Medical and scientific judgment should be exercised in deciding whether an isolated vital sign abnormality should be classified as an AE.

If a clinically significant vital sign abnormality is a sign of a disease or syndrome (e.g., high blood pressure), only the diagnosis (i.e., hypertension) should be recorded on the AE eCRF.

Observations of the same clinically significant vital sign abnormality from visit to visit should not be repeatedly recorded on the AE eCRF, unless the etiology changes. The initial severity of the event should be recorded, and the severity or seriousness should be updated any time the event worsens.

5.3.5.6 Abnormal Liver Function Tests

The finding of an elevated ALT or AST (> 3 times the baseline value) in combination with either an elevated total bilirubin (> 2 times the ULN) or clinical jaundice in the absence of cholestasis or other causes of hyperbilirubinemia is considered to be an indicator of severe liver injury. Therefore, investigators must report as an AE the occurrence of either of the following:

- Treatment-emergent ALT or AST > 3 times the baseline value in combination with total bilirubin > 2 times the ULN (of which ≥ 35% is direct bilirubin)
- Treatment-emergent ALT or AST > 3 times the baseline value in combination with clinical jaundice

The most appropriate diagnosis or (if a diagnosis cannot be established) the abnormal laboratory values should be recorded on the AE eCRF (see Section 5.3.5) and reported to the Sponsor immediately (i.e., no more than 24 hours) after learning of the event, either as an SAE or a non-serious AE of special interest (see Section 5.4.2).

5.3.5.7 Deaths

All deaths that occur during the protocol-specified AE reporting period (see Section 5.3.1), regardless of relationship to study drug, must be recorded on the AE eCRF and immediately reported to the Sponsor (see Section 5.4.2), including death attributed to progression of CHC.

Death should be considered an outcome and not a distinct event. The event or condition that caused or contributed to the fatal outcome should be recorded as the single medical concept on the AE eCRF. Generally, only one such event should be reported. The term “sudden death” should only be used for the occurrence of an abrupt and unexpected death due to presumed cardiac causes in a patient with or without pre-existing heart disease, within 1 hour of the onset of acute symptoms or, in the case of an unwitnessed death, within 24 hours after the patient was last seen alive and stable. If the cause of death is unknown and cannot be ascertained at the time of reporting, “unexplained death” should be recorded on the AE eCRF. If the cause of death later becomes available (e.g., after autopsy), “unexplained death” should be replaced by the established cause of death.

If the death is attributed to progression of CHC, "CHC progression” should be recorded on the AE eCRF.

5.3.5.8 Pre-Existing Medical Conditions

A pre-existing medical condition is one that is present at the screening visit for this study. Such conditions should be recorded on the General Medical History and Baseline Conditions eCRF.

A pre-existing medical condition should be recorded as an AE **only** if the frequency, severity, or character of the condition worsens during the study. When recording such events on the AE eCRF, it is important to convey the concept that the pre-existing condition has changed by including applicable descriptors (e.g., “more frequent headaches”).

5.3.5.9 Lack of Efficacy or Worsening of CHC

Medical occurrences or symptoms of deterioration that are anticipated as part of CHC should be recorded as AEs if judged by the investigator to have unexpectedly worsened in severity or frequency or changed in nature at any time during the study. When recording an unanticipated worsening of CHC on the AE eCRF, it is important to convey the concept that the condition has changed by including applicable descriptors (e.g., “accelerated chronic hepatitis C”).

5.3.5.10 Hospitalization or Prolonged Hospitalization

Any AE that results in hospitalization or prolonged hospitalization should be documented and reported as an SAE (per the definition of SAE in Section 5.2.2), except as outlined below.

The following hospitalization scenarios are not considered to be SAEs:

- Hospitalization for respite care
- Hospitalization for a pre-existing condition, provided that all of the following criteria are met:

  The hospitalization was planned prior to the study or was scheduled during the study when elective surgery became necessary because of the expected normal progression of the disease.

  The patient has not experienced an AE.

5.3.5.11 Overdoses

Study drug overdose is the accidental or intentional use of the drug in an amount higher than the dose being studied. An overdose or incorrect administration of study drug is not an AE unless it results in untoward medical effects.

Any study drug overdose or incorrect administration of study drug should be noted on the Study Drug Administration eCRF.

All AEs associated with an overdose or incorrect administration of study drug should be recorded on the AE eCRF. If the associated AE meets criteria for seriousness, the event should be reported to the Sponsor immediately (i.e. no more than 24 hours) after learning of the event (see Section 5.4.2).

5.4 IMMEDIATE REPORTING REQUIREMENTS FROM INVESTIGATOR TO SPONSOR

Certain events require immediate reporting to allow the Sponsor to take appropriate measures to address potential new risks in a clinical trial. The investigator must report such events to the Sponsor immediately; under no circumstances should reporting take place more than 24 hours after the investigator learns of the event. The following is a list of events that the investigator must report to the Sponsor within 24 hours after learning of the event, regardless of relationship to study drug:

- Serious adverse events
- Non-serious AEs of special interest
- Pregnancies

The investigator must report new significant follow-up information for these events to the Sponsor immediately (i.e., no more than 24 hours) after becoming aware of the information. New significant information includes the following:

- New signs or symptoms or a change in the diagnosis
- Significant new diagnostic test results
- Change in causality based on new information
- Change in the event's outcome, including recovery
- Additional narrative information on the clinical course of the event

Investigators must also comply with local requirements for reporting SAEs to the local health authority and IRB/EC.

5.4.1 Emergency Medical Contacts

MEDICAL MONITOR (ROCHE MEDICAL RESPONSIBLE) CONTACT INFORMATION

Primary Contact

Medical Monitor: Ewa Pastuszka, M.D. Ph.D

Telephone No.: +44 1707 365953

Mobile Telephone No.: +44 77868 56599

Secondary Contact

Medical Monitor: Astrid Scalori, M.D.

Telephone No.: +44 1707 365927

Mobile Telephone No.: +44 7824623395

To ensure the safety of study patients, an Emergency Medical Call Center Help Desk will access the Roche Medical Emergency List, escalate emergency medical calls, provide medical translation service (if necessary), connect the investigator with a Roche Medical Monitor, and track all calls. The Emergency Medical Call Center Help Desk will be available 24 hours per day, 7 days per week. Toll-free numbers for the Help Desk and Medical Monitor contact information will be distributed to all investigators (see "Protocol Administrative and Contact Information and List of Investigators").

5.4.2 Reporting Requirements for SAEs and Non-Serious AEs of Special Interest

For reports of SAEs and non-serious AEs of special interest, investigators should record all case details that can be gathered immediately (i.e., no more than 24 hours) on the AE eCRF and submit the report via the electronic data capture (EDC) system. A report will be generated and sent to Roche Safety Risk Management by the EDC system.

In the event that the EDC system is unavailable, a paper SAE/Non-Serious AE of Special Interest Case Report Form (CRF) and Fax Coversheet should be completed and faxed to Roche Safety Risk Management or its designee immediately (i.e., no more than 24 hours) after learning of the event, using the fax numbers provided to investigators (see “Protocol Administrative and Contact Information and List of Investigators”). Once the EDC system is available, all information will need to be entered and submitted via the EDC system.

5.4.3 Reporting Requirements for Pregnancies

5.4.3.1 Pregnancies in Female Patients

Female patients of childbearing potential will be instructed to immediately inform the investigator if they become pregnant during the study or within 6 months after the study or according to the locally approved label for Copegus after the last dose of study drug. A Pregnancy Report eCRF should be completed by the investigator immediately (i.e., no more than 24 hours) after learning of the pregnancy and submitted via the EDC system. A pregnancy report will automatically be generated and sent to Roche Safety Risk Management. Pregnancy should not be recorded on the AE eCRF. The investigator should discontinue Copegus and counsel the patient, discussing the risks of the pregnancy and the possible effects on the fetus. Monitoring of the patient should continue until conclusion of the pregnancy.

In the event that the EDC system is unavailable, a Pregnancy Report Worksheet and Pregnancy Fax Coversheet should be completed and faxed to Roche Safety Risk Management or its designee immediately (i.e., no more than 24 hours) after learning of the pregnancy, using the fax numbers provided to investigators (see “Protocol Administrative and Contact Information and List of Investigators”).

5.4.3.2 Pregnancies in Female Partners of Male Patients

Male patients will be instructed through the Informed Consent Form to immediately inform the investigator if their partner becomes pregnant during the study or within 6 months after the study or according to the locally approved label for Copegus after the last dose of study drug. A Pregnancy Report eCRF should be completed by the investigator immediately (i.e., no more than 24 hours) after learning of the pregnancy and submitted via the EDC system. Attempts should be made to collect and report details of the course and outcome of any pregnancy in the partner of a male patient exposed to study drug. The pregnant partner will need to sign an Authorization for Use and Disclosure of Pregnancy Health Information to allow for follow up on her pregnancy. Once the authorization has been signed, the investigator will update the Pregnancy Report eCRF with additional information on the course and outcome of the pregnancy. An investigator who is contacted by the male patient or his pregnant partner may provide information on the risks of the pregnancy and the possible effects on the fetus to support an informed decision in cooperation with the treating physician and/or obstetrician.

In the event that the EDC system is unavailable, follow reporting instructions provided in Section 5.4.3.1.

5.4.3.3 Abortions

Any spontaneous abortion should be classified as an SAE (as the Sponsor considers spontaneous abortions to be medically significant events), recorded on the AE eCRF, and reported to the Sponsor immediately (i.e., no more than 24 hours) after learning of the event (see Section 5.4.2).

5.4.3.4 Congenital Anomalies/Birth Defects

Any congenital anomaly/birth defect in a child born to a female patient or female partner of a male patient exposed to study drug should be classified as an SAE, recorded on the AE eCRF, and reported to the Sponsor immediately (i.e., no more than 24 hours) after learning of the event (see Section 5.4.2).

5.5 FOLLOW-UP OF PATIENTS AFTER AEs

5.5.1 Investigator Follow Up

The investigator should follow each AE until the event has resolved to baseline grade or better, the event is assessed as stable by the investigator, the patient is lost to follow up, or the patient withdraws consent. Every effort should be made to follow all SAEs considered to be related to study drug or trial-related procedures until a final outcome can be reported.

During the study period, resolution of AEs (with dates) should be documented on the AE eCRF and in the patient's medical record to facilitate source data verification. If, after follow-up, return to baseline status or stabilization cannot be established, an explanation should be recorded on the AE eCRF.

All pregnancies reported during the study should be followed until pregnancy outcome. If the EDC system is not available at the time of pregnancy outcome, follow reporting instructions provided in Section 5.4.3.1.

5.5.2 Sponsor Follow Up

For SAEs, non-serious AEs of special interest, and pregnancies, the Sponsor or a designee may follow up by telephone, fax, electronic mail, and/or a monitoring visit to obtain additional case details and outcome information (e.g., from hospital discharge summaries, consultant reports, autopsy reports) in order to perform an independent medical assessment of the reported case.

5.6 POSTSTUDY AEs

At the Study Completion/Early Termination Visit, the investigator should instruct each patient to report to the investigator any subsequent AEs that the patient's personal physician believes could be related to prior study drug treatment or study procedures.

The investigator should notify the Sponsor of any death, SAE, or other AE of concern occurring at any time after a patient has discontinued study participation if the event is believed to be related to prior study drug treatment or study procedures. The Sponsor should also be notified if the investigator becomes aware of the development of cancer or a congenital anomaly/birth defect in a subsequently conceived offspring of a patient that participated in this study.

The investigator should report these events to Roche Safety Risk Management on the AE eCRF. If the AE eCRF is no longer available, the investigator should report the event directly to Roche Safety Risk Management via telephone (see "Protocol Administrative and Contact Information and List of Investigators").

5.7 EXPEDITED REPORTING TO HEALTH AUTHORITIES, INVESTIGATORS, IRBs, AND ECs

To determine reporting requirements for SAE cases, the Sponsor will assess the expectedness of these events using the following reference documents:

- RO5024048 IB
- Prescribing information for boceprevir (USPI), Pegasys (local), and Copegus (local)

The Sponsor will compare the severity of each event and the cumulative event frequency reported for the study with the severity and frequency reported in the applicable reference document.

Reporting requirements will also be based on the investigator's assessment of causality and seriousness, with allowance for upgrading by the Sponsor as needed.

6. STATISTICAL CONSIDERATIONS AND ANALYSIS PLAN

Final efficacy and safety analyses of the total study population will be conducted at the end of the study after all patients have completed all study assessments and the database has been cleaned and closed.

6.1 DETERMINATION OF SAMPLE SIZE

Boceprevir with Pegasys/Copegus has been shown to provide approximately 34% SVR in patients with poor prior response defined as < 1 $\log_{10}$ IU/mL decline after a 4 week lead in with PEG-IFN/RBV (Bacon et al. 2011). An SVR rate of 45%–55% for an experimental group is expected on the basis of an anticipated reduction in treatment-emergent resistance to boceprevir and the potential added benefit of RO5024048.

Assuming response rates of 45%–55% for an experimental treatment group, a sample size of 30 patients per experimental treatment group will provide 90% confidence interval widths of ± 15%.

6.2 SUMMARIES OF CONDUCT OF STUDY

Enrollment, eligibility violations, and study drug administration will be summarized by treatment group. Patient disposition will be summarized by treatment group and will include whether treatment was completed or discontinued early and the reason for early treatment discontinuation.

6.3 SUMMARIES OF TREATMENT GROUP COMPARABILITY

Demographic and baseline characteristics will be summarized by treatment group. Descriptive summaries of continuous data will include the group mean, standard deviation, median, minimum and maximum, and sample size. Descriptive summaries of discrete data will include frequencies and percentages.

6.4 EFFICACY ANALYSES

Primary and secondary efficacy analyses will include all patients who were randomized and received at least one dose of study medication. Patients will be analyzed according to the treatment group to which they were randomized.

No formal hypothesis testing will be done in this exploratory study. As a result, no adjustment for a Type 1 error will be made to account for the multiplicity of analyses.

6.4.1 Primary Efficacy Endpoint

The primary efficacy endpoint will be SVR-12 (actual) outcome, with response defined as an unquantifiable (less than the LLOQ [< 25 IU/mL]) serum HCV RNA 12 weeks after the actual end of treatment (a single last unquantifiable HCV RNA within 8–20 weeks after the last day of study drug administration).

The primary efficacy endpoint (SVR-12 [actual]) for each of the treatment groups will be summarized using descriptive statistics (n, percentage, and confidence interval based upon binomial probabilities).

6.4.2 Secondary Efficacy Endpoints

The virologic response rate for each treatment group, and the corresponding confidence interval estimates will be summarized for relevant secondary endpoints (e.g., SVR-4 [actual] and SVR-24 [actual]).

Mean changes from baseline in HCV RNA ($\log_{10}$ IU/mL) levels for each treatment group and their confidence interval estimates will also be summarized.

6.5 SAFETY ANALYSES

Safety analyses will include all randomized patients who received at least one dose of study treatment (RO5024048, Pegasys, Copegus, or boceprevir) and had at least one postbaseline safety assessment with patients allocated to the treatment group associated with the regimen actually received.

In the analysis of safety data, all AEs or abnormal laboratory findings occurring during treatment and the treatment-free follow-up period will be summarized for each treatment group. Adverse events will be summarized by body system and event within each body system.

Laboratory data will be analyzed according to Roche's "International Guideline for the Handling and Reporting of Laboratory Data" (Roche Guideline). All laboratory data will be converted to International System (SI) units for reporting and processing purposes. For those laboratory tests lacking sufficiently common procedures, in order to use a universal "normal range," transformations of scale will be executed at the time of conversion to SI units, using the investigator- or laboratory-supplied reference range to transform to a common (Roche-supplied standard) range. This will be done for a limited set of tests (that is, the enzyme tests), and in almost all instances the investigator or central laboratory lower limit will be supplanted by 0. The definitions regarding transforming laboratory test results, apart from changes to SI units, are provided in the Roche Guideline and apply to all tests for which the investigator's ranges are not provided. Marked laboratory abnormalities will be summarized according to the guideline.

Safety analyses to determine if treatment groups are safe to proceed will use all available data reported from central laboratory results. Unscheduled local laboratory tests may be ordered per investigator discretion and may be used for the individual management of the patient. A duplicate sample should simultaneously be sent to the central laboratory for analysis.

The absolute and percentage changes from baseline in vital sign parameters will be computed, and potentially serious changes will be noted. Electrocardiogram interval measurements will be summarized by nominal time. Appropriate summary statistics will be provided for all vital sign parameters. For a change from baseline in any vital sign parameter to be considered potentially serious, the change must be an increase or decrease of > 20% in the direction of worsening, and the absolute value must be outside the corresponding reference range as shown below:

- Systolic BP < 85 or > 180 mmHg OR diastolic BP > 110 mmHg
- Heart rate < 45 or > 120 beats per minute

6.6 SUBGROUP ANALYSES

The following subgroup analyses will be performed to examine the consistency of treatment effects:

- Hepatitis C virus genotype subtype (1a, 1b)
- IL28B genotype (CC, CT, TT)
- Pretreatment cirrhotic status (cirrhotic, non-cirrhotic)

The proportion of patients with relapse of virologic response will be summarized by treatment group.

6.7 RESISTANCE ANALYSES

Blood samples for resistance-monitoring studies will be taken according to the Schedule of Assessments (see Appendix A). Patients' samples for resistance-monitoring studies will be selected on the basis of results obtained from the COBAS TaqMan HCV v2.0 test.

Resistance-monitoring studies will be performed as detailed in Section 4.5.1.8.

The genotype of boceprevir- and/or RO5024048-resistant samples (defined as those samples for which FC ≥ 3) will be determined through population sequencing of the NS5B coding region.

The quotient of antiviral IC_{50} values determined in the mutant and in the wild-type replicons will be determined as FC values to describe the level of resistance to RO5024048 and/or boceprevir.

Samples (encompassing the NS3 protease, NS5B polymerase, and/or other regions of the HCV genome) may also be used to test for resistance to other small antiviral molecules. Sampling procedures, storage conditions, and shipment instructions will be provided in a supplemental laboratory manual.

The findings from these analyses will be listed for each patient selected for these analyses. Data will be reported following the FDA guidelines for the reporting of drug-resistance data.

The rate of resistance will be summarized by treatment group.

6.8 PHARMACOKINETIC ANALYSES

Data may be combined with those from other studies for population PK analyses using non-linear mixed effects modeling to describe the time course of the plasma concentrations of RO4995855. The influence of demographic covariates (such as body weight, age, and sex) on RO4995855 PK parameters (i.e., clearance and volume of distribution) will be investigated if appropriate.

6.9 EXPLORATORY ANALYSES

An exploratory analysis will be performed to assess the PD response and emergence of resistance. Graphical and statistical techniques such as linear, non-linear, and logistic regression will be used to explore potential relationships between treatment regimen, PK, viral, and host factors and safety and efficacy parameters.

6.9.1 Biomarker Analyses

Biomarker analyses will explore the impact of baseline IP-10 on treatment response. The analyses will be exploratory and will not be part of the final Clinical Study Report. Analysis will be completed as appropriate. Details of the exploratory analyses will be provided in a separate SAP.

6.10 INTERIM ANALYSES

At least one interim analysis of efficacy and safety data will be performed to inform the RO5024048 clinical development plan. An interim analysis will be performed for safety after all patients complete Week 12, and for safety and efficacy after all patients complete Week 36 and potentially at other timepoints as needed. Virologic response with a confidence interval will be summarized by visit. Presentations of resistance rates and of safety data including tabulations of all AEs, all laboratory abnormalities, and reasons for dose modifications and/or discontinuation from trial medications will be prepared.

7. DATA COLLECTION AND MANAGEMENT

7.1 DATA QUALITY ASSURANCE

Roche will be responsible for data management of this study, including quality checking of the data. Data entered manually will be collected via EDC using eCRFs. Sites will be responsible for data entry into the EDC system. In the event of discrepant data, Roche will request data clarification from the sites. The sites will resolve discrepant data electronically in the EDC system.

Roche will produce an EDC Study Specification document that describes the quality checking to be performed on the data. Central laboratory data will be sent directly to Roche, using Roche's standard procedures to handle and process the electronic transfer of these data.

Electronic CRFs and correction documentation will be maintained in the EDC system's audit trail. System backups for data stored at Roche and records retention for the study data will be consistent with Roche's standard procedures.

7.2 ELECTRONIC CASE REPORT FORMS

Electronic CRFs are to be completed using a Sponsor-designated EDC system. Sites will receive training and a have access to a manual for appropriate eCRF completion. Electronic CRFs will be submitted electronically to Roche and should be handled in accordance with instructions from Roche.

All eCRFs should be completed by designated, trained site staff. Electronic CRFs should be reviewed and electronically signed and dated by the investigator or a designee.

At the end of the study, the investigator will receive patient data for his or her site in a readable format on a compact disc that must be kept with the study records. Acknowledgement of receipt of the compact disc is required.

7.3 SOURCE DATA DOCUMENTATION

Study monitors will perform ongoing source data verification to confirm that critical protocol data (i.e., source data) entered into the eCRFs by authorized site personnel are accurate, complete, and verifiable from source documents.

Source documents (paper or electronic) are those in which patient data are recorded and documented for the first time. They include, but are not limited to, hospital records, clinical and office charts, laboratory notes, memoranda, patient-reported outcomes, evaluation checklists, pharmacy dispensing records, recorded data from automated instruments, copies of transcriptions that are certified after verification as being accurate and complete, microfiche, photographic negatives, microfilm or magnetic media, X-rays, patient files, and records kept at pharmacies, laboratories, and medicotechnical departments involved in a clinical trial.

Before study initiation, the types of source documents that are to be generated will be clearly defined in the Trial Monitoring Plan. This includes any protocol data to be entered directly into the eCRFs (i.e., no prior written or electronic record of the data) and considered source data.

Source documents that are required to verify the validity and completeness of data entered into the eCRFs must not be obliterated or destroyed and must be retained per the policy for retention of records described in Section 7.5.

To facilitate source data verification, the investigators and institutions must provide the Sponsor direct access to applicable source documents and reports for trial-related monitoring, Sponsor audits, and IRB/EC review. The investigational site must also allow inspection by applicable health authorities.

7.4 USE OF COMPUTERIZED SYSTEMS

When clinical observations are entered directly into an investigational site's computerized medical record system (i.e., in lieu of original hardcopy records), the electronic record can serve as the source document if the system has been validated in accordance with health authority requirements pertaining to computerized systems used in clinical research. An acceptable computerized data collection system allows preservation of the original entry of data. If original data are modified, the system should maintain a viewable audit trail that shows the original data as well as the reason for the change, name of the person making the change, and date of the change.

7.5 RETENTION OF RECORDS

Records and documents pertaining to the conduct of this study and the distribution of IMP, including eCRFs, Informed Consent Forms, laboratory test results, and medication inventory records must be retained by the Principal Investigator for at least 15 years after completion or discontinuation of the study or for the length of time required by relevant national or local health authorities, whichever is longer. After that period of time, the documents may be destroyed, subject to local regulations.

No records may be disposed of without the written approval of Roche. Written notification should be provided to Roche prior to transferring any records to another party or moving them to another location.

8. <u>ETHICAL CONSIDERATIONS</u>

8.1 COMPLIANCE WITH LAWS AND REGULATIONS

This study will be conducted in full conformance with the ICH E6 guideline for Good Clinical Practice and the principles of the Declaration of Helsinki, or the laws and regulations of the country in which the research is conducted, whichever affords the greater protection to the individual. The study will comply with the requirements of the ICH E2A guideline (Clinical Safety Data Management: Definitions and Standards for Expedited Reporting). Studies conducted in the United States or under a U.S. Investigational New Drug Application (IND) will comply with U.S. FDA regulations and applicable local, state, and federal laws. Studies conducted in the EU/EEA will comply with the EU Clinical Trial Directive (2001/20/EC).

8.2 INFORMED CONSENT

Roche’s sample Informed Consent Form (and ancillary sample Informed Consent Forms such as a Child’s Assent or Caregiver's Informed Consent Form, if applicable) will be provided to each site. If applicable, it will be provided in a certified translation of the local language. Roche or its designee must review and approve any proposed deviations from Roche's sample Informed Consent Forms or any alternate consent forms proposed by the site (collectively, the “Consent Forms”) before IRB/EC submission. The final IRB/EC-approved Consent Forms must be provided to Roche for health authority submission purposes according to local requirements.

The Consent Forms must be signed and dated by the patient or the patient’s legally authorized representative before his or her participation in the study. The case history or clinical records for each patient shall document the informed consent process and that written informed consent was obtained prior to participation in the study.

The Consent Forms should be revised whenever there are changes to study procedures or when new information becomes available that may affect the willingness of the patient to participate. The final revised IRB/EC-approved Consent Forms must be provided to Roche for health authority submission purposes.

Patients must be reconsented to the most current version of the Consent Forms (or to a significant new information/findings addendum in accordance with applicable laws and IRB/EC policy) during their participation in the study. For any updated or revised Consent Forms, the case history or clinical records for each patient shall document the informed consent process and that written informed consent was obtained using the updated/revised Consent Forms for continued participation in the study.

A copy of each signed Informed Consent Form must be provided to the patient or the patient's legally authorized representative. All signed and dated Consent Forms must remain in each patient's study file or in the site file and must be available for verification by study monitors at any time.

For sites in the United States, each Informed Consent Form may also include patient authorization to allow use and disclosure of personal health information in compliance with the U.S. Health Insurance Portability and Accountability Act of 1996 (HIPAA). If the site uses a separate Authorization Form for patient authorization for use and disclosure of personal health information under the HIPAA regulations, the review, approval, and other processes outlined above apply except that IRB review and approval may not be required per study site policies.

8.3 INSTITUTIONAL REVIEW BOARD OR EC

This protocol, the Informed Consent Forms, any information to be given to the patient, and relevant supporting information must be submitted to the IRB/EC by the principal investigator and reviewed and approved by the IRB/EC before the study is initiated. In addition, any patient recruitment materials must be approved by the IRB/EC.

The principal investigator is responsible for providing written summaries of the status of the study to the IRB/EC annually or more frequently in accordance with the requirements, policies, and procedures established by the IRB/EC. Investigators are also responsible for promptly informing the IRB/EC of any protocol amendments (see Section 9.4).

In addition to the requirements for reporting all AEs to the Sponsor, investigators must comply with requirements for reporting SAEs to the local health authority and IRB/EC. Investigators may receive written IND safety reports or other safety-related communications from Roche. Investigators are responsible for ensuring that such reports are reviewed and processed in accordance with health authority requirements and the policies and procedures established by their IRB/EC and archived in the site's study file.

8.4 CONFIDENTIALITY

Roche maintains confidentiality standards by coding each patient enrolled in the study through assignment of a unique patient identification number. This means that patient names are not included in data sets that are transmitted to any Roche location.

Patient medical information obtained by this study is confidential and may only be disclosed to third parties as permitted by the Informed Consent Form (or separate authorization for use and disclosure of personal health information) signed by the patient, unless permitted or required by law.

Medical information may be given to a patient's personal physician or other appropriate medical personnel responsible for the patient's welfare for treatment purposes.

Data generated by this study must be available for inspection upon request by representatives of the U.S. FDA and other national and local health authorities; Roche monitors, representatives, and collaborators; and the IRB/EC for each study site, as appropriate.

8.5 FINANCIAL DISCLOSURE

Investigators will provide the Sponsor with sufficient, accurate financial information in accordance with local regulations to allow the Sponsor to submit complete and accurate financial certification or disclosure statements to the appropriate health authorities. Investigators are responsible for providing information on financial interests during the course of the study and for 1 year after completion of the study (i.e., LPLV).

9. STUDY DOCUMENTATION, MONITORING, AND ADMINISTRATION

9.1 STUDY DOCUMENTATION

The investigator must maintain adequate and accurate records to enable the conduct of the study to be fully documented, including but not limited to the protocol, protocol amendments, Informed Consent Forms, and documentation of IRB/EC and governmental approval. In addition, at the end of the study, the investigator will receive the patient data, which includes an audit trail containing a complete record of all changes to data.

9.2 SITE INSPECTIONS

Site visits will be conducted by Roche or an authorized representative for inspection of study data, patients' medical records, and eCRFs. The investigator will permit national and local health authorities, Roche monitors, representatives, and collaborators, and the IRBs/ECs to inspect facilities and records relevant to this study.

9.3 PUBLICATION OF DATA AND PROTECTION OF TRADE SECRETS

The results of this study may be published or presented at scientific meetings. If this is foreseen, the investigator agrees to submit all manuscripts or abstracts to Roche prior to submission. This allows the Sponsor to protect proprietary information and to provide comments based on information from other studies that may not yet be available to the investigator.

Roche will comply with the requirements for publication of study results. In accordance with standard editorial and ethical practice, Roche will generally support publication of multicenter trials only in their entirety and not as individual center data. In this case, a coordinating investigator will be designated by mutual agreement.

Authorship will be determined by mutual agreement and in line with International Committee of Medical Journal Editors authorship requirements. Any formal publication of the study in which contribution of Roche personnel exceeded that of conventional monitoring will be considered as a joint publication by the investigator and the appropriate Roche personnel.

Any inventions and resulting patents, improvements, and/or know-how originating from the use of data from this study will become and remain the exclusive and unburdened property of Roche, except where agreed otherwise.

9.4 PROTOCOL AMENDMENTS

Any protocol amendments will be prepared by the Sponsor. Protocol amendments will be submitted to the IRB/EC and to regulatory authorities in accordance with local regulatory requirements.

Approval must be obtained from the IRB/EC and regulatory authorities (as locally required) before implementation of any changes, except for changes necessary to eliminate an immediate hazard to patients or changes that involve logistical or administrative aspects only (e.g., change in Medical Monitor or contact information).

10. REFERENCES

Adijawah BS, Hare B, Caron PR, et al. Rapid decrease of wild-type hepatitis C virus on boceprevir treatment. Antiviral Therapy 2009;14:591–5.

Bacon B, Gordon S, Lawitz E, et. al. Boceprevir for previously treated chronic HCV genotype 1 infection. N Engl J Med 2011;364:1207-17.

Berman K and Kwo PY. Boceprevir, an NS3 protease inhibitor of HCV. Clin Liver Dis 2009;13:429-39.

Boceprevir Investigator’s Brochure. June 2010.

Bukh J, Miller RH, Purcell RH. Genetic heterogeneity of hepatitis C virus: quasispecies and genotypes. Semin Liv Dis 1995;15:41–63.

Chatel-Chaix L, Baril M, Lamarre D. Hepatitis C virus NS3/4A protease inhibitors: A light at the end of the tunnel. Viruses 2010;2:1752–65.

Copegus [package insert]. South San Francisco, CA: Roche; 2011.

Daniel S. Chronic hepatitis C treatment patterns in African American patients: an update. Am J Gastroenterol 2005;100:716–22.

Darling JM, Aerssens J, Fanning G, et al. Quantitation of pretreatment serum interferon-γ-inducible protein-10 improves the predictive value of an IL28B gene polymorphism for hepatitis C treatment response. Hepatology 2011;53:14–22.

Di Bisceglie AM, Conjeevaram HS, Fried MW, et al. Ribavirin as therapy for chronic hepatitis C. A randomized, double-blind, placebo-controlled trial. Ann Int Med 1995;123:897–903.

Di Bisceglie AM, Nelson DR, Gane E, et al. VX-222 with TVR alone or in combination with peginterferon alfa-2a and ribavirin in treatment-naive patients with chronic hepatitis C: ZENITH study interim results. J Hepatol 2011;54:S540.

Dieterich DT, Rizzetto M, Manns MP. Management of chronic hepatitis C patients who have relapsed or not responded to pegylated interferon alfa plus ribavirin. J Viral Hepat 2009;16:833–43.

Dusheiko G, Main J, Thomas H, et al. Ribavirin treatment for patients with chronic hepatitis C: Results of a placebo-controlled trial. J Hepatol 1996;25:591–8.

EASL/International consensus conference on hepatitis C. Consensus statement. J Hepatol 1999;31(Suppl):3–8.

Foster GR, Buggisch P, Marcellin P, et al. Four-week treatment with GS-9256 and tegobuvir (GS-9190), ± RBV ± PEG, results in enhanced viral suppression on follow-up PEG/RBV therapy, in genotype 1a/1b HCV patients. J Hepatol 2011;54:S172.

Fried MW, Shiffman ML, Reddy KR, et al. Peginterferon alfa-2a plus ribavirin for chronic hepatitis C virus. Nature 2002;436:939–45.

Gale M, Foy EM. Evasion of intracellular host defense by hepatitis C virus. Nature 2005;436:939–45.

Gane EJ, Roberts SK, Stedman CAM, et al. Oral combination therapy with nucleoside polymerase inhibitor (RG7128) and danoprevir for chronic hepatitis C genotype 1 infection (INFORM-1): a randomized, double-blind, placebo-controlled, dose-escalation trial. Lancet 2010;376:1467–75.

Ge D, Fellay J, Thompson AJ, et al. Genetic variation in IL28B predicts hepatitis C treatment-induced viral clearance. Nature 2009;461:399–401.

Ghany MG, Strader DB, Thomas DL, et al. AASLD practice guidelines: diagnosis, management, and treatment of hepatitis C: an update. Hepatology 2009;49:1335–74.

Ghosal A, Yuan Y, Tong W, et. al. Characterization of human liver enzymes involved in the biotransformation of boceprevir, a hepatitis C virus protease inhibitor. DMD 2011;39:510-21.

Gilbert BE, Knight V. Mini-review: biochemistry and clinical applications for ribavirin. Antimicro Agents Chemother 1986;30:201–5.

Hadziyannis SJ, Sette H Jr, Morgan TR, et al. Peginterferon alfa-2a and ribavirin combination therapy in chronic hepatitis C. Ann Int Med 2004;140:346–55.

Jensen DM, Marcellin P, Freilich B, et al. Re-treatment of patients with chronic hepatitis C who do not respond to peginterferon-α2b. Ann Intern Med 2009;150:528–40.

Jensen DM, Wedemeyer H, Herring R Jr, et al. High rates of early viral response, promising safety profile and lack of resistance-related breakthrough in HCV GT 1/4 patients treated with RG7128 plus PegIFN alfa-2a (40KD)/RBV: Planned week 12 interim analysis from the ProPel study. Hepatology 2010;52:Abstract 81.

Kamal SM, Nasser IA. Hepatitis C genotype 4: What we know and what we don’t yet know. Hepatology 2008;47:1371–83.

Khakoo SI, Alexander G, Goedert JJ, et al. Genetic variation in IL28B and spontaneous clearance of hepatitis C virus. Nature 2009;461:798–801.

Kwo PK, McCone J, Schiff ER, et. al. Efficacy of boceprevir, an NS3 protease inhibitor, in combination with peginterferon alfa-2b and ribavirin in treatment-naïve patients with genotype 1 hepatitis C infection (SPRINT-1); an open-label, randomized, multicenter phase 2 trial. Lancet 2010;376:705-16.

Lauer GM, Walker BD. Hepatitis C virus infection. N Engl J Med 2001;345:41–52.

Lavanchy, D. Global surveillance and control of hepatitis C: Report of a WHO consultation. J Viral Hepat 1999;6:35–47.

Le Pogam S, Yan JM, Kosaka A, et al. No evidence of drug resistance or baseline S282T resistance mutation among GT1 and GT4 HCV infected patients on nucleoside polymerase inhibitor (RG7128) and PegIFN/RBV combination treatment for up to 12 weeks: Interim analysis from the ProPel study. Hepatology 2010;52:Abstract 799.

Lok AS, Gardiner DF, Lawitz E, et al. Quadruple therapy with BMS-790052, BMS-650032 and PEG-IFN/RBV for 24 weeks in 100% SVR12 in HCV genotype 1 null responders. J Hepatol 2011;54:S536.

Manns MP, McHutchison JG, Gordon SC, et al. Peginterferon alfa-2b plus ribavirin compared with interferon alfa-2b plus ribavirin for initial treatment of chronic hepatitis C: a randomized trial. Lancet 2001;358:958–65.

Mauss S, Berg T, Rockstroh J, et al. Hepatology: A clinical textbook. 2nd edition 2010;187. Available from: www.HepatologyTextbook.com.

McHutchison JG, Everson GT, Gordon SC, et al. Telaprevir with peginterferon and ribavirin for chronic HCV genotype 1 infection. N Engl J Med 2009;360:1827–38.

McHutchison JG, Manns MP, Muir AJ, et al. Telaprevir for previously treated chronic HCV infection. N Engl J Med 2010;362:1292–303.

Monto A, Schooley RT, Lai JC, et al. Lessons from HIV therapy applied to viral hepatitis therapy: Summary of a workshop. Am J Gastroenterol 2010;105:989–1004.

NIH. Recommendations from the National Institutes of Health Consensus Development conference statement: Management of hepatitis C: 2002. Hepatology 2002;36:1039.

NIH, NIDDK. Chronic hepatitis C: Current disease management. Available from: http://digestive.niddk.nih.gov/ddiseases/pubs/chronichepc/index.htm.

Pegasys Investigator’s Brochure. July 2010.

Pegasys [package insert]. South San Francisco, CA: Roche; 2011.

Pockros P, Jensen D, Tsai N, et al. First SVR data with the nucleoside analogue polymerase inhibitor RO5024048 (RG7128) combinated with peginterferon/ribavirin in treatment-naive HCV G1/4 patients: interim analysis from the Jump-C trial. J Hepatol 2011;54:S538.

Poordad F, McCone J, Bacon B, et. al. Boceprevir for untreated chronic HCV genotype 1 infection. N Engl J Med 2011;364:1195-1206.

Powdrill MH, Bernatchez JA, Götte M. Inhibitors of the hepatitis C virus RNA-dependent RNA polymerase NS5B. Viruses 2010;2:2169–95.

RO5024048 Investigator’s Brochure. February 2011.

Roche Palo Alto LLC. Inhibition of RNA synthesis by β-D-2′-deoxy-2′-fluoro-2′-C-methylcytidine (TO4995855) in the HCV subgenomic replicon system. 2006;Report No. 1022744.

Roche International Clinical Operating Guideline No. 3. International Guideline for the Handling and Reporting of Laboratory Data.

Rong L, Dahari H, Ribeiro RM, et. al. Rapid emergence of protease resistance in hepatitis C virus. Sci Transl Med 2010;2:30ra32.

Sarrazin C, Lim S, Qin X, et al. Kinetic analysis or viral rebound and drug-resistant viral variant dynamics in patients treated with ITMN-191 (RG7227) monotherapy suggests a high barrier to viral escape. Hepatology 2009;50(Suppl 1):953A.

Sarrazin C, Rouzier R, Wagner F, et. al. SCH 503034, a novel hepatitis C virus protease inhibitor, plus pegylated interferon α-2b for genotype 1 nonresponders. Gastroenterology 2007;132:1270-78.

Sarrazin C, Zeuzem S. Resistance to direct antiviral agents in patients with hepatitis C virus infection. Gastroenterology 2010;138:447–62.

Schiff E, Poordad F, Jacobson I, et. al. Boceprevir (B) combination therapy in null responders (NR): response dependent on interferon responsiveness. J Hepatol 2008;48:Abstract 104.

Sidwell RW, Robins RK, Hillyard IW. Ribavirin: an antiviral agent. Pharmacol Ther 1979;6:123–46.

SmPC eMC: Summary of Product Characteristics last updated on the eMC: July 16, 2010. Available from: http://www.medicines.org.uk/emc/medicine/10081.

Soriano V, Peters MG, Zeuzem S. New therapies for hepatitis C virus infection. Clin Infec Dis 2009;48:313–20.

Strader DB, Wright T, Thomas DL, et al. Diagnosis, management and treatment of hepatitis C. Hepatology 2004;39:1147–71.

Susser S, Welch C, Wang Y, et. al. Characterization of resistance to the protease inhibitor boceprevir in hepatitis C virus-infected patients. Heptatology 2009;50:1709-18.

Thompson AJ, Muir AJ, Sulkowski MS, et al. IL28B Polymorphism improves viral kinetics and is the strongest pre-treatment predictor of SVR in HCV-1 patients. Gastroenterology. 2010;139:120–9.

Trey C, Burns DG, Saunders SJ. Treatment of hepatic coma by exchange blood transfusion. N Engl J Med 1966;274:473–81.

Victrelis [package insert]. Whitehouse Station, NJ: Merck; 2011.

Zeuzem S. Heterogeneous virologic response rates to interferon based therapy in patients with chronic hepatitis C: Who responds less well? Ann Intern Med 2004;140:370–81.

Zeuzem S, Buggisch P, Agarwal K, et al. Dual, triple, and quadruple combination treatment with a protease inhibitor (GS-9256) and a polymerase inhibitor (GS-9190) alone and in combination with ribavirin (RBV) or PegIFN/RBV for up to 28 days in treatment naïve, genotype 1 HCV subjects. Hepatology 2010;52:Abstract LB-1.

Appendix A
Schedule of Assessments

	Screen	Re-screen	Base-line	Treatment Period (wk)												Treatment-Free Follow-Up Period (wk) [a]			
	Up to –8 wk	–35 to –1 d	Day 1	1	2	4	8	12	16	20	24	30	36	42	48	4	12	24	PW [b]
Informed consent	X																		
Demographics	X																		
Complete medical history and physical examination	X [c]	X [d]	X [e]																X [e]
HCV genotyping	X																		
Chest X-ray [f]	X	X [d]																	
Liver biopsy [g]	X																		
AFP, ultrasound, and endoscopy [h]	X																		
Immunology [i]	X	X [d]																	
Ophthalmologic exam [j]	X	X [d]																	
Psychiatric evaluation [k]	X	X [d]				X	Every 4 weeks during study												
ECG [l]	X			X	X	X		X			X								X [d]
Pregnancy test [m]	X [n]	X [n]	X [n]			X		Home-based pregnancy test every 4 weeks during treatment and following last dose of Copegus in accordance with the locally approved label											
Vital signs [o]	X	X	X	X	X	X [p]	X [p]	X [p]	X [p]	X [p]	X [p]	X	X	X	X	X	X	X	X
Hematology [q]	X	X	X	X	X	X	X	X	X	X	X	X	X	X	X	X	X		X
Chemistry [r]	X [s]	X	X	X	X	X	X	X	X	X	X	X	X	X	X	X	X		X

Appendix A (cont'd)
Schedule of Assessments

	Screen	Re-screen	Base-line	Treatment Period (wk)												Treatment-Free Follow-Up Period (wk) [a]			
	Up to –8 wk	–35 to –1 d	Day 1	1	2	4	8	12	16	20	24	30	36	*42*	48	4	12	24	PW [b]
Urinalysis with urine chemistry for protein/creatinine ratio [t]			X	X	X	X	X	X	X	X	X	X	X	*X*	X		X		X
TSH, uric acid, and fasting cholesterol and triglycerides	X	X [d]	X					X			X		X		X		X		X
IL28B genotyping	X																		
HCV RNA [u]	X	X	X [v]	X	X	X	X	X	X	X	X	X	X	*X*	X	X	X	X	X
Drug-resistance monitoring			X	X	X	X	X	X	X	X	X	X	X	*X*	X	X	X	X	X
Plasma and RNA RCR (optional)			X [v]	X [w]	X [v]	X [v]	X [v]	X [v]			X [v]				X		X		
DNA RCR (optional)			X																
PK sampling			X				X												
Serum IP-10			X [w]																
Serum bank	X	X	X				X					X	X	*X*	X	X	X	X	X
AEs x, concomitant medications, compliance	X [y]	X [y]	X				X					X	X	*X*	X	X	X	X	X
Dispense study medication			X				X					X	X	*X*					

Ab = antibody; AE = adverse event; AFP = alpha-fetoprotein; AMA = antimitochrondrial Ab; ANA = antinuclear Ab; ASMA = antismooth muscle Ab; AT = antitrypsin; CK = creatine kinase; CRCL = creatinine clearance; CT = computerized tomography; HAV = hepatitis A virus; HBsAg = hepatitis B surface antigen; HCV = hepatitis C virus; Hgb = hemoglobin; INR = International Normalized Ratio; Serum IP-10 = interferon gamma-induced protein 10kDa; MCH = mean corpuscular hemoglobin; MCHC = mean corpuscular hemoglobin concentration; MCV = mean corpuscular volume; MRI = magnetic resonance imaging; PD = pharmacodynamic; PK = pharmacokinetic; PT = prothrombin time; PW = Premature Withdrawal Visit; RCR = Roche Clinical Repository; SAE = serious adverse event; TSH = thyroid-stimulating hormone.

[a] Assessments to be performed 4, 12, and 24 weeks after the last dose of study treatment in all patients who completed treatment. Patients who prematurely discontinue from the study will be assessed for safety and efficacy at 4*,* 12*, and 24* weeks after the last dose of study treatment.

[b] All patients who prematurely withdraw are recommended to complete the PW Visit as soon as possible after the last dose of study medication and proceed with treatment-free follow up.

[c] Includes height and weight.

[d] Repeat if any change in medical condition is noted since the screening examination.

[e] Physical examination only.

[f] Only for patients with pre-existing pulmonary disease or current respiratory symptoms.

[g] All patients must have a liver biopsy or fibroscan assessment. Fibroscan must be completed within 12 calendar months of the first dose (an elasticity score of ≥ 12.5 kPa will be used to designate incomplete/transition to cirrhosis (i.e., "cirrhotic") a score of ≥ 9.5 kPa but < 12.5 kPa will require a patient to undergo a liver biopsy to determine whether he/she is designated as "cirrhotic" or "non-cirrhotic").

[h] Ultrasound within 6 months prior to randomization. If a patient has had a CT or MRI scan within the 6-month period that includes cuts of the entire liver, these assessments may be substituted for an ultrasound as the liver imaging procedure. Endoscopy within 2 years prior to randomization.

[i] Anti-HCV Ab, Anti-HAV IgM Ab, HBsAg, anti-HIV Ab, ceruloplasmin, alpha-1 AT, AMA, ANA, ASMA, ferritin, and transferrin.

[j] In all patients, complete ophthalmologic examination, including dilated fundus examination, visual acuity, visual field testing, and color visual testing to be performed by a trained ophthalmologist.

[k] Only for patients with a history of severe psychiatric disease.

[l] All ECGs are to be obtained predose and prior to a meal or laboratory/PK sampling collection if these procedures are scheduled at the same time. All patients will have 12-lead ECGs performed in a resting supine position, in triplicate (within 10 minutes).

[m] For fertile or potentially fertile females only, and at any time a secondary amenorrhea of > 1 week occurs.

[n] Negative serum test at screening and a negative urine test confirmed by a negative serum test collected within 24 hours before first dose of study drug.

[o] Systolic and diastolic blood pressure and pulse.

[p] Body weight.

[q] Complete blood count with differential (RBC count, Hgb, hematocrit, MCV, MCH, MCHC, leukocytes, neutrophils, basophils, eosinophils, monocytes, lymphocytes, reticulocytes, platelets, haptoglobin); coagulation panel (PT/INR).

[r] Alanine transaminase, AST, gamma-glutamyl transpeptidase, lactate dehydrogenase, total/direct/indirect bilirubin, alkaline phosphatase, total protein, albumin, BUN/urea, creatinine, glucose, calcium, magnesium, phosphorus, sodium, potassium, chloride, bicarbonate, amylase, lipase, CK, CK-MB, troponins (only if CK-MB is abnormal after baseline), lactic acid, and calculation for CRCL.

[s] HbA1c only for patients with Type 1 and 2 diabetes.

[t] Urine chemistry for protein, microalbumin, and creatinine. Urinalysis consisting of color and clarity, specific gravity, pH, protein, glucose, ketones, bilirubin, urobilinogen, blood, nitrite, and leukocyte esterase. Urine microscopic for WBC, WBC clumps, RBC, RBC clumps, epithelial cells, crystals, bacteria, yeast, mucous, and casts (see Appendix H for urine protein/creatinine calculations).

[u] For patients showing viral load breakthrough, relapse, or partial response, a confirmatory sample must be obtained within 2 weeks.

[v] Collected predose.

[w] Collected prior to Pegasys dose.

[x] To be accompanied by symptom-directed physical examination as appropriate.

[y] After informed consent, but prior to initiation of study medications, only SAEs caused by a protocol-mandated intervention will be collected (e.g., SAEs related to invasive procedures such as biopsies).

Appendix B
Liver Biopsy Scores

All patients must have a liver biopsy consistent with CHC. Patients not yet designated as having cirrhosis or incomplete/transition to cirrhosis must have had a liver biopsy within 24 calendar months prior to the first dose. For patients with cirrhosis or incomplete/transition to cirrhosis have, there is no time frame for the biopsy. The biopsies must be scored using one of the scales described below.

Transition to cirrhosis will be classified as a Metavir score of 3, a Batts and Ludwig score of 3, a Scheuer score of 3, or an Ishak Modified HAI score of 4 with nodules or > 3 bridges or a score of 5.

Patients with transition to or incomplete cirrhosis are to be classified as cirrhotic.

METAVIR FIBROSIS SCORE

- 0 No fibrosis
- 1 Stellate enlargement of portal tract but without septa formation
- 2 Enlargement of portal tract with rare septa formation
- 3 Numerous septa without cirrhosis
- 4 Cirrhosis

BATTS and LUDWIG

- 0 No fibrosis; normal connective tissue
- 1 Portal fibrosis; fibrous portal expansion
- 2 Periportal fibrosis; periportal or rare portal–portal septa
- 3 Septal fibrosis; fibrous septa with architectural distortion; no obvious cirrhosis
- 4 Cirrhosis

THE SCHEUER SYSTEM

- 0 None
- 1 Enlarged, fibrotic portal tracts
- 2 Periportal or portal-portal septa but intact architecture
- 3 Fibrosis with architectural distortion but no obvious cirrhosis
- 4 Probable or definite cirrhosis

ISHAK MODIFIED HAI FIBROSIS SCORE

0 No fibrosis

1 Fibrous expansion of some portal areas, with or without short fibrous septa

2 Fibrous expansion of most portal areas, with or without short fibrous septa

3 Fibrous expansion of most portal areas, with occasional portal to portal bridging

4 Fibrous expansion of portal areas, with marked bridging (portal to portal) as well as portal to central

5 Marked bridging (portal to portal and/or portal to central) with occasional nodules (incomplete cirrhosis)

6 Cirrhosis, probable or definite

Appendix C
Creatinine Clearance Calculation

ESTIMATED CREATININE CLEARANCE ACCORDING TO COCKCROFT GAULT

CONVENTIONAL UNITS

$$\text{Creatinine Clearance (mL/min)} = \frac{(140 - \text{age}) \times \text{Body Weight (kg)}}{72 \times \text{Serum Creatinine (mg/dL)}}$$

Female CRCL = Male CRCL × 0.85

SI UNITS

$$\text{Creatinine Clearance (mL/sec)} = \frac{(140 - \text{age}) \times \text{Body Weight (kg)}}{72 \times \text{Serum Creatinine } (\mu\text{mol/L}) \times 0.6786}$$

Female CRCL = Male CRCL × 0.85

CONVERSION FACTOR FOR CRCL

Conventional units (mL/min) = SI units (mL/sec)/0.0167

SI units (mL/sec) = Conventional units (mL/min) × 0.0167

CONVERSION FACTOR FOR SERUM CREATININE

Conventional units (mg/dL) = SI units (μmol/L)/88.4

SI units (μmol/L) = Conventional units (mg/dL) × 88.4

Appendix D
Child–Pugh Classification of Severity of Liver Disease

Table D1
Child–Pugh Scoring on Select Parameters

	Points Scored for Increasing Abnormality		
Clinical and biochemical measurements	1	2	3
Encephalopathy (grade) [a]	None	1 or 2	3 or 4
Ascites [b]	Absent	Slight	Moderate
Bilirubin (mg/100 mL)	< 2	2–3	> 3
Albumin (g/100 mL)	> 3.5	2.8–3.5	< 2.8
Prothrombin time (International Normalized Ratio) [c]	< 1.7	1.7–2.3	> 2.3

Note: With increasing abnormality of each of the five parameters measured, 1, 2, or 3 points are scored. Grade A: 5 or 6 points. Grade B: 7–9 points. Grade C: 10–15 points.

[a] According to grading of Trey, Burns, and Saunders (1966).

[b] As determined by physical examination alone.

[c] Prothrombin time results should be reported and used for calculations only as International Normalized Ratios because of variations in the methods used and reference ranges for controls (expressed in seconds).

Appendix E
Package Inserts for Boceprevir, Pegasys, and Copegus

http://www.merck.com/product/usa/pi_circulars/v/victrelis/victrelis_pi.pdf
http://www.ema.europa.eu/docs/en_GB/document_library/EPAR_-_Product_Information/human/002332/WC500109786.pdf
http://www.gene.com/gene/products/information/pegasys/pdf/pi.pdf
http://www.gene.com/gene/products/information/pegasys/pdf/Copegus_pi.pdf

Appendix F
Dose Modifications, Interruptions, and Delays

The intention of the protocol is for patients demonstrating a response to therapy to remain on study drug until the completion of the study treatment period. However, it is possible that some patients will encounter transient or prolonged AEs at some juncture during their participation in the trial necessitating test drug dose adjustment. To minimize the effects of these modifications on the eventual evaluation of the safety, tolerability, and efficacy of test drug regimens, the principles in the following sections will be used to adjust the dose of test drugs.

RO5024048 AND BOCEPREVIR DOSE INTERRUPTION OR DISCONTINUATION

No dose modifications will be allowed for RO5024048 or boceprevir.

RO5024048 must be discontinued immediately and the Sponsor's Medical Monitor contacted within 24 hours if any of the following are confirmed and **considered related** to the study drug:

- Creatinine clearance: ≥ 35% decrease from baseline or < 60 mL/min (< 1.00 mL/sec) confirmed by repeat test, preferably within 1 week
- Any clinically significant Grade 4 laboratory abnormality confirmed by repeat test, preferably within 1 week, unless otherwise defined
- Any SAE assessed as related to RO5024048

It is important that patients receive the full dose of RO5024048 and/or boceprevir. However, moderate or severe adverse reactions (clinical and/or laboratory) may necessitate dose interruption or discontinuation of RO5024048 and/or boceprevir.

If a dose of RO5024048 or boceprevir is missed and the patient remembers the same day, the patient must take the missed dose if the next scheduled dose is more than 4 hours away. The patient should not take two doses at the same time or within 4 hours of each other.

Patients who discontinue all study drugs should return for the PW visit as soon as possible after their last dose of study medication, according to Appendix A. Patients who prematurely discontinue from the study will be assessed for safety and efficacy at 4 and 12 weeks after the last dose of study treatment. In addition, patients who prematurely discontinue and have unquantifiable HCV RNA will be assessed for efficacy at 12 weeks after the last dose of study treatment as long as they remain HCV RNA unquantifiable at their last assessment.

PEGASYS DOSE INTERRUPTIONS, DELAYS, AND MODIFICATIONS

If a Pegasys dose is delayed but eventually administered, the following guidelines should be used for the next scheduled dose(s).

Dose delayed by 1 or 2 days

Administer next dose on usual dosing day of the week (e.g., if Monday is the usual dosing day and the dose is delayed until Wednesday, the next dose may be administered as usual on Monday).

Dose delayed by 3-5 days

Administer subsequent doses every fifth or sixth day until the patient is back to his or her original schedule (e.g., if Monday is the usual dosing day and the dose is delayed until Saturday, the next dose should be administered on Thursday, the following dose on Tuesday, then the dose after that as usual on Monday).

Dose delayed by 6 days

Withhold the dose for that week, then continue on the usual schedule the following week (e.g., if Monday is the usual dosing day but the patient is not ready to be dosed until the following Sunday, the dose is considered to have been withheld and the next injection should be for the following week's dose on Monday).

Dose delayed by ≥ 7 days

The investigator may reintroduce Pegasys at any time and, if necessary, dose the patient every fifth or sixth day until the patient resumes weekly dosing on his/her usual scheduled day.

Moderate or severe adverse reactions (clinical and/or laboratory) may require a dose reduction of Pegasys. Decremental reductions should be uniform across centers and patients. Toward this end, downward reductions should follow the guidelines shown in Table F1.

Table F1

General Pegasys Dose-Reduction Scheme

Full Pegasys dose	1.0 mL	180 µg
One-level reduction	0.75 mL	135 µg
Two-level reduction	0.5 mL	90 µg
Three-level reduction	0.25 mL	45 µg

Once a patient's dose has been reduced or withheld for an adverse reaction, the investigator may attempt to increase the dose back to or toward that which was originally assigned if the event has resolved or improved.

Specific dose adjustment guidelines for Pegasys are provided below for elevated serum ALT, depression (see Table F2), neutropenia (see Table F3), thrombocytopenia (see Table F3), and low Hgb (see Table F4).

Dose Adjustments for Elevated Serum ALT

Fluctuations in abnormalities of liver function tests are common in patients with CHC. As with other alfa IFNs, increases in ALT levels above baseline have been observed in patients treated with Pegasys, including patients with a virologic response.

In patients with progressive ALT increases above baseline, the dose of Pegasys should be reduced by one level. If ALT increases are progressive despite dose reduction or accompanied by increased bilirubin or evidence of hepatic decompensation, therapy should be immediately discontinued.

Table F2
Guidelines for Modification or Discontinuation of Pegasys and for Scheduling Visits for Patients with Depression

	Initial Management (4–8 weeks)		Depression		
Depression Severity	Dose Modification	Visit Schedule	Remains Stable	Improves	Worsens
Mild	No change	Evaluate once weekly by visit and/or phone	Continue weekly visit schedule	Resume normal visit schedule	(See moderate or severe depression)
Moderate	Decrease Pegasys dose by one level adjustment (in some cases a two-level adjustment may be needed)	Evaluate once weekly (office visit at least every other week)	Consider psychiatric consultation. Continue reduced dosing	If symptoms improve and are stable for 4 weeks, may resume normal visit schedule. Continue reduced dosing or return to normal dose	(See severe depression)
Severe	Discontinue Pegasys permanently	Obtain immediate psychiatric consultation	Psychiatric therapy necessary		

Table F3
Dose Adjustments for Neutropenia and Thrombocytopenia

Dose Adjustment for Low Absolute Neutrophil Count	
ANC	Pegasys Dose Reduction
< 750 cells/mm^3	Reduce Pegasys to 135 µg
< 500 cells/mm^3	Withhold Pegasys until ANC > 750 cells/mm^3, reinstitute at 90 µg, and monitor
**Dose Adjustment for Low Platelet Count**	
Platelet Count	Pegasys Dose Reduction
< 50,0000 cells/mm^3	Reduce Pegasys to 90 µg
< 25,000 cells/mm^3	Withhold treatment and conduct weekly or more frequent monitoring

ANC = absolute neutrophil count.

Table F4
Dose Adjustment for Low Hemoglobin

	One Level Adjustment for Copegus if:	Withhold Copegus if:
In patients without significant cardiovascular disease	Hemoglobin < 10 g/dL	Hemoglobin < 8.5 g/dL

COPEGUS DOSE MODIFICATIONS

It is important that patients receive as close to the full dose of Copegus as possible. However, moderate or severe adverse reactions (clinical and/or laboratory) may necessitate a dose modification of Copegus. It is therefore recommended that the Copegus dose be reduced in gradual stepwise decrements as depicted in Table F5.

Table F5
General Copegus Dose-Reduction Scheme

	Baseline Body Weight	
	≥ 75 kg	< 75 kg
Full Copegus dose	1200 mg/day (AM: 3 tabs; PM: 3 tabs)	1000 mg/day (AM: 3 tabs; PM: 2 tabs)
One-level reduction	1000 mg/day (AM: 3 tabs; PM: 2 tabs)	800 mg/day (AM: 2 tabs; PM: 2 tabs)
Two-level reduction	800 mg/day (AM: 2 tabs; PM: 2 tabs)	600 mg/day (AM: 2 tabs; PM: 1 tab)
Three-level reduction	600 mg/day (AM: 2 tabs; PM: 1 tab)	400 mg/day (AM: 1 tab; PM: 1 tab)

At the discretion of the investigator, patients who experience a substantial hemoglobin decrease during treatment should have weekly or biweekly hematologic testing and clinical evaluation. Patients who have > 3 g/dL decrease from baseline in their Hgb concentration should have an appropriate work-up for anemia—including reticulocyte count—and a search for sources of bleeding.

If deemed necessary by the investigator, patients should be assessed at a minimum every 2 weeks until the AE is resolved or the patient is stable. Persistent adverse reactions may require two- or three-level dose reductions or necessitate that the dose be temporarily withheld.

Once the Copegus dose has been reduced or withheld for an adverse reaction, an attempt may be made to restart Copegus and/or increase the dose if the event has resolved or improved, at the discretion of the investigator. The Copegus dose should be increased in 200-mg increments by increasing the number of tablets taken daily in a stepwise manner until the desired level is achieved. It is not recommended to escalate to the fully prescribed dose of Copegus.

Appendix G
Management of Specific Adverse Events and Laboratory Abnormalities

RENAL ABNORMALITIES

If RO5024048 is discontinued because of renal abnormalities, the following measures will be performed as soon as possible:

- Blood measurements

    Complete blood count with differential and peripheral smear for schistocytes

    Chemistry with calculated creatinine clearance

- Blood pressure measurements
- Urine panel

    Urinalysis (dipstick and microscopic)

    Urine chemistry (first void of the day, chilled sample) for protein, creatinine, and microalbumin (for details and calculation of urine protein/creatinine ratio when urine protein is reported as undetectable)

    Repeat urinalysis to confirm the presence of hematuria at the time of the additional evaluation (for hematuria only)

    Repeat urine analysis to evaluate for leukocytes and the presence of infection (for hematuria only)

    Urine culture and sensitivity (for hematuria only)

    Urine protein electrophoresis

- Nephrology consultation
- Renal ultrasound or renal biopsy, if indicated per consultant evaluation

The investigator will use the additional clinical and laboratory measures to evaluate the relationship between renal abnormalities and other potential clinical findings and will contact the Roche Medical Monitor within 24 hours for further discussion. All events should be appropriately monitored (including obtaining laboratory test) until resolution or return to baseline.

ELEVATED BLOOD PRESSURE/DECREASED RENAL FUNCTION

During RO5024048 treatment and for 2 weeks thereafter, patients with elevations in systolic blood pressure of > 160 mmHg and diastolic blood pressure of > 100 mmHg confirmed (preferably within 1 week) or increases of 30 mmHg systolic or 20 mmHg diastolic from baseline will be scheduled for weekly blood pressure measurements and laboratory procedures described below.

During RO5024048 treatment and for 2 weeks thereafter, patients with confirmed (preferably within 1 week) CRCL < 70 mL/min (< 1.17mL/sec) but ≥ 60 mL/min (≥ 1 mL/sec) OR a decrease from baseline ≥ 25% but < 35% OR urine protein/creatinine ratio ≥ 0.4 (≥ 45.25 mg/mmol) but < 0.5 (< 56.51 mg/mmol) will be scheduled for weekly visits and laboratory assessments described below.

- Blood measurements

  Complete blood count with differential and peripheral smear for schistocytes

  Chemistry with calculated creatinine clearance

- Urine panel

  Urinalysis (dipstick and microscopic)

  Urine chemistry (first void of the day, chilled sample) for protein, creatinine and microalbumin (for details and calculation of urine protein/creatinine ratio when urine protein is reported as undetectable)

The investigator will use the additional clinical and laboratory measures to evaluate the relationship between elevated blood pressure findings/renal abnormalities and other potential clinical findings and will contact the Medical Monitor within 24 hours for further discussion. All events should be appropriately monitored (including obtaining laboratory test) until resolution or return to baseline.

OPHTHALMOLOGIC ABNORMALITIES

Patients who during the study develop blurred vision, decreased visual acuity, photophobia, or eye pain, which are not attributable to another clinical diagnosis (e.g., allergies), should be scheduled immediately for an ophthalmologic evaluation including, but not limited to visual acuity, visual field, color vision, and a dilated fundus exam.

The investigator should contact the Medical Monitor within 24 hours for further discussion.

ANEMIA/DECREASED HEMOGLOBIN

Refer to Appendix F, Table F4: Dose Adjustment for Low Hemoglobin (Copegus Dose Modification), Appendix F, Table F5: General Copegus Dose Reduction Scheme, and the boceprevir package insert (Victrelis USPI 2011) for dose-modification instructions related to occurrence of anemia or decreased hemoglobin.

Appendix G
Management of Specific Adverse Events and Laboratory Abnormalities (cont.)

NEUTROPENIA

Refer to Appendix F, Table F3: Dose Adjustment for Low Absolute Neutrophil Count; and Table F5: General Copegus Dose-Reduction Scheme and the boceprevir package insert for dose-modification instructions related to the occurrence of neutropenia or decreased neutrophil count.

Appendix H
Urinary Protein Monitoring Plan

Urine protein/creatinine ratio is a study stopping rule and requires the measurement of urine protein and creatinine concentration. A low urine protein concentration may be physiologic or due to a diluted urine sample caused by a rapid water load prior to voiding.

The methodology used by the central laboratory has a LLOQ of 4 mg/dL. In cases where urine protein is < 4 mg/dL (below the LLOQ), the urine protein/creatinine ratio will be reported by the central laboratory as “Unable to calculate.” To determine a urine protein/creatinine ratio in all patients, the following steps should be taken.

1. On study visit days, patients are required to collect a first void of the day (typically the morning void) urine sample. Concentrations of both urine protein and creatinine will be higher, reducing the risk of undetectable levels and avoiding the variation in protein excretion caused by varying levels of activity during the day. The urine sample should be kept chilled until it is brought to the clinic.
2. In cases where the central laboratory reports a urine protein concentration of < 4 mg/dL (below the LLOQ), the site should calculate the urine protein/creatinine ratio by substituting the LLOQ (i.e., 4 mg/dL) for the urine protein results. The choice of using the LLOQ as the assigned value is a conservative approach when calculating the protein/creatinine ratio. Manual calculation of a urine protein/creatinine ratio will allow assessment of the exclusion criterion, the stopping rule, or the need for additional follow up and will also enable reporting of summary statistics for each visit.

For sites using SI units: To determine if the entry criteria or renal stopping rules have been met, please convert the reported ratio result from SI units to conventional units by multiplying by 0.00884 the result reported in SI units. An example of the conversion of the urine protein/creatinine ratio result from SI to conventional units is as follows:

$$4.52 \text{ mg/mmol} \times 0.00884 = 0.04$$

Appendix H
Urinary Protein Monitoring Plan (cont.)

Table H1
Urinary Monitoring Plan for Protein

<table>
<tr><th>Timepoint</th><th>Finding</th><th colspan="2">Action Required by Investigator</th></tr>
<tr><td rowspan="2">Baseline to Week 24</td><td rowspan="2">Urine chemistry protein/Cr ratio ≥ 0.5 and greater than the baseline.</td><td colspan="2">Confirm value with repeat urine measurement within 1 week</td></tr>
<tr><td>IF CONFIRMED:
Discontinue RO5024048 immediately. Collect urine for protein electrophoresis. Continue urine monitoring until protein/Cr ratio returns to baseline or stabilizes. Refer to nephrologist.</td><td>IF NOT CONFIRMED:
Continue therapy with RO5024048, continue urine assessments per protocol.</td></tr>
<tr><td>Weeks 24–60</td><td>Urine chemistry protein/Cr ratio ≥ 0.5 and greater than the baseline</td><td colspan="2">Collect urine for protein electrophoresis.
Continue urine monitoring weekly until protein/Cr ratio returns to baseline or stabilized. Refer to nephrologist.</td></tr>
</table>

Appendix I
Division of Aids Table for Grading the Severity of Adult and Pediatric Adverse Events

Quick Reference

The Division of AIDS Table for Grading the Severity of Adult and Pediatric Adverse Events ("DAIDS AE grading table") is a descriptive terminology which can be utilized for Adverse Event (AE) reporting. A grading (severity) scale is provided for each AE term.

General Instructions

Estimating Severity Grade

If the need arises to grade a clinical AE that is not identified in the DAIDS AE grading table, use the category "Estimating Severity Grade" located at the top of Page 3. For AEs that are not listed in the table but will be collected systematically for a study/trial, protocol teams are highly encouraged to define study-specific severity scales within the protocol or an appendix to the protocol. (Please see "Template Wording for the Expedited Adverse Event Reporting Section of DAIDS-sponsored Protocols".) This is particularly important for laboratory values because the "Estimating Severity Grade" category only applies to clinical symptoms.

Grading Adult and Pediatric AEs

The DAIDS AE grading table includes parameters for grading both Adult and Pediatric AEs. When a single set of parameters is not appropriate for grading specific types of AEs for both Adult and Pediatric populations, separate sets of parameters for Adult and/or Pediatric populations (with specified respective age ranges) are given in the table. If there is no distinction in the table between Adult and Pediatric values for a type of AE, then the single set of parameters listed is to be used for grading the severity of both Adult and Pediatric events of that type.

Determining Severity Grade

If the severity of an AE could fall under either one of two grades (e.g., the severity of an AE could be either Grade 2 or Grade 3), select the higher of the two grades for the AE.

Definitions

Basic Self-care Functions	Adult<br>Activities such as bathing, dressing, toileting, transfer/movement, continence, and feeding.<br>Young Children<br>Activities that are age and culturally appropriate (e.g., feeding self with culturally appropriate eating implement).
LLN	Lower limit of normal
Medical Intervention	Use of pharmacologic or biologic agent(s) for treatment of an AE.
NA	Not Applicable
Operative Intervention	Surgical OR other invasive mechanical procedures.
ULN	Upper limit of normal
Usual Social & Functional Activities	Adult<br>Adaptive tasks and desirable activities, such as going to work, shopping, cooking, use of transportation, pursuing a hobby, etc.<br>Young Children<br>Activities that are age and culturally appropriate (e.g., social interactions, play activities, learning tasks, etc.).

Appendix I
Division of Aids Table For Grading the Severity of Adult and Pediatric Adverse Events (cont.)

Contents

Clinical	Page
Estimating Severity Grade	3
Systemic	3
Infection	4
Injection Site Reactions	4
Skin – Dermatological	5
Cardiovascular	5
Gastrointestinal	7
Neurologic	9
Respiratory	12
Musculoskeletal	12
Genitourinary	13
Ocular/Visual	14
Endocrine/Metabolic	14

Laboratory	Page
Hematology	16
Chemistries	17
Urinalysis	20

Appendix I
Division of Aids Table For Grading the Severity of Adult and Pediatric Adverse Events (cont.)

CLINICAL				
**PARAMETER**	**GRADE 1 MILD**	**GRADE 2 MODERATE**	**GRADE 3 SEVERE**	**GRADE 4 POTENTIALLY LIFE-THREATENING**
**ESTIMATING SEVERITY GRADE**				
Clinical adverse event NOT identified elsewhere in this DAIDS AE grading table	Symptoms causing no or minimal interference with usual social & functional activities	Symptoms causing greater than minimal interference with usual social & functional activities	Symptoms causing inability to perform usual social & functional activities	Symptoms causing inability to perform basic self-care functions OR Medical or operative intervention indicated to prevent permanent impairment, persistent disability, or death
**SYSTEMIC**				
Acute systemic allergic reaction	Localized urticaria (wheals) with no medical intervention indicated	Localized urticaria with medical intervention indicated OR Mild angioedema with no medical intervention indicated	Generalized urticaria OR Angioedema with medical intervention indicated OR Symptomatic mild bronchospasm	Acute anaphylaxis OR Life-threatening bronchospasm OR laryngeal edema
Chills	Symptoms causing no or minimal interference with usual social & functional activities	Symptoms causing greater than minimal interference with usual social & functional activities	Symptoms causing inability to perform usual social & functional activities	NA
Fatigue<br>Malaise	Symptoms causing no or minimal interference with usual social & functional activities	Symptoms causing greater than minimal interference with usual social & functional activities	Symptoms causing inability to perform usual social & functional activities	Incapacitating fatigue/ malaise symptoms causing inability to perform basic self-care functions
Fever (nonaxillary)	37.7 – 38.6°C	38.7 – 39.3°C	39.4 – 40.5°C	> 40.5°C
Pain (indicate body site)<br>DO NOT use for pain due to injection (See Injection Site Reactions: Injection site pain)<br>See also Headache, Arthralgia, and Myalgia	Pain causing no or minimal interference with usual social & functional activities	Pain causing greater than minimal interference with usual social & functional activities	Pain causing inability to perform usual social & functional activities	Disabling pain causing inability to perform basic self-care functions OR Hospitalization (other than emergency room visit) indicated

Basic Self-care Functions – Adult: Activities such as bathing, dressing, toileting, transfer/movement, continence, and feeding.

Basic Self-care Functions – Young Children: Activities that are age and culturally appropriate (e.g., feeding self with culturally appropriate eating implement).

Usual Social & Functional Activities – Adult: Adaptive tasks and desirable activities, such as going to work, shopping, cooking, use of transportation, pursuing a hobby, etc.

Usual Social & Functional Activities – Young Children: Activities that are age and culturally appropriate (e.g., social interactions, play activities, learning tasks, etc.).

12-28-04 Page 3 of 20 Version 1.0

Appendix I
Division of Aids Table For Grading the Severity of Adult and Pediatric Adverse Events (cont.)

CLINICAL				
**PARAMETER**	**GRADE 1 MILD**	**GRADE 2 MODERATE**	**GRADE 3 SEVERE**	**GRADE 4 POTENTIALLY LIFE-THREATENING**
Unintentional weight loss	NA	5 – 9% loss in body weight from baseline	10 – 19% loss in body weight from baseline	≥ 20% loss in body weight from baseline OR Aggressive intervention indicated [e.g., tube feeding or total parenteral nutrition (TPN)]
**INFECTION**				
Infection (any other than HIV infection)	Localized, no systemic antimicrobial treatment indicated AND Symptoms causing no or minimal interference with usual social & functional activities	Systemic antimicrobial treatment indicated OR Symptoms causing greater than minimal interference with usual social & functional activities	Systemic antimicrobial treatment indicated AND Symptoms causing inability to perform usual social & functional activities OR Operative intervention (other than simple incision and drainage) indicated	Life-threatening consequences (e.g., septic shock)
**INJECTION SITE REACTIONS**				
Injection site pain (pain without touching) Or Tenderness (pain when area is touched)	Pain/tenderness causing no or minimal limitation of use of limb	Pain/tenderness limiting use of limb OR Pain/tenderness causing greater than minimal interference with usual social & functional activities	Pain/tenderness causing inability to perform usual social & functional activities	Pain/tenderness causing inability to perform basic self-care function OR Hospitalization (other than emergency room visit) indicated for management of pain/tenderness
Injection site reaction (localized)				
**Adult > 15 years**	Erythema OR Induration of 5x5 cm – 9x9 cm (or 25 cm^2 – 81cm^2)	Erythema OR Induration OR Edema > 9 cm any diameter (or > 81 cm^2)	Ulceration OR Secondary infection OR Phlebitis OR Sterile abscess OR Drainage	Necrosis (involving dermis and deeper tissue)
**Pediatric ≤ 15 years**	Erythema OR Induration OR Edema present but ≤ 2.5 cm diameter	Erythema OR Induration OR Edema > 2.5 cm diameter but < 50% surface area of the extremity segment (e.g., upper arm/thigh)	Erythema OR Induration OR Edema involving ≥ 50% surface area of the extremity segment (e.g., upper arm/thigh) OR Ulceration OR Secondary infection OR Phlebitis OR Sterile abscess OR Drainage	Necrosis (involving dermis and deeper tissue)

Basic Self-care Functions – Adult: Activities such as bathing, dressing, toileting, transfer/movement, continence, and feeding.

Basic Self-care Functions – Young Children: Activities that are age and culturally appropriate (e.g., feeding self with culturally appropriate eating implement).

Usual Social & Functional Activities – Adult: Adaptive tasks and desirable activities, such as going to work, shopping, cooking, use of transportation, pursuing a hobby, etc.

Usual Social & Functional Activities – Young Children: Activities that are age and culturally appropriate (e.g., social interactions, play activities, learning tasks, etc.).

12-28-04 Page 4 of 20 Version 1.0

Appendix I
Division of Aids Table For Grading the Severity of Adult and Pediatric Adverse Events (cont.)

CLINICAL				
**PARAMETER**	**GRADE 1 MILD**	**GRADE 2 MODERATE**	**GRADE 3 SEVERE**	**GRADE 4 POTENTIALLY LIFE-THREATENING**
Pruritis associated with injection<br>See also Skin: Pruritis (itching - no skin lesions)	Itching localized to injection site AND Relieved spontaneously or with < 48 hours treatment	Itching beyond the injection site but not generalized OR Itching localized to injection site requiring ≥ 48 hours treatment	Generalized itching causing inability to perform usual social & functional activities	NA
**SKIN – DERMATOLOGICAL**				
Alopecia	Thinning detectable by study participant (or by caregiver for young children and disabled adults)	Thinning or patchy hair loss detectable by health care provider	Complete hair loss	NA
Cutaneous reaction – rash	Localized macular rash	Diffuse macular, maculopapular, or morbilliform rash OR Target lesions	Diffuse macular, maculopapular, or morbilliform rash with vesicles or limited number of bullae OR Superficial ulcerations of mucous membrane limited to one site	Extensive or generalized bullous lesions OR Stevens-Johnson syndrome OR Ulceration of mucous membrane involving two or more distinct mucosal sites OR Toxic epidermal necrolysis (TEN)
Hyperpigmentation	Slight or localized	Marked or generalized	NA	NA
Hypopigmentation	Slight or localized	Marked or generalized	NA	NA
Pruritis (itching – no skin lesions)<br>(See also Injection Site Reactions: Pruritis associated with injection)	Itching causing no or minimal interference with usual social & functional activities	Itching causing greater than minimal interference with usual social & functional activities	Itching causing inability to perform usual social & functional activities	NA
**CARDIOVASCULAR**				
Cardiac arrhythmia (general)<br>(By ECG or physical exam)	Asymptomatic AND No intervention indicated	Asymptomatic AND Non-urgent medical intervention indicated	Symptomatic, non-life-threatening AND Non-urgent medical intervention indicated	Life-threatening arrhythmia OR Urgent intervention indicated
Cardiac-ischemia/infarction	NA	NA	Symptomatic ischemia (stable angina) OR Testing consistent with ischemia	Unstable angina OR Acute myocardial infarction

Basic Self-care Functions – Adult: Activities such as bathing, dressing, toileting, transfer/movement, continence, and feeding.

Basic Self-care Functions – Young Children: Activities that are age and culturally appropriate (e.g., feeding self with culturally appropriate eating implement).

Usual Social & Functional Activities – Adult: Adaptive tasks and desirable activities, such as going to work, shopping, cooking, use of transportation, pursuing a hobby, etc.

Usual Social & Functional Activities – Young Children: Activities that are age and culturally appropriate (e.g., social interactions, play activities, learning tasks, etc.).

12-28-04 Page 5 of 20 Version 1.0

Appendix I
Division of Aids Table For Grading the Severity of Adult and Pediatric Adverse Events (cont.)

CLINICAL				
**PARAMETER**	**GRADE 1 MILD**	**GRADE 2 MODERATE**	**GRADE 3 SEVERE**	**GRADE 4 POTENTIALLY LIFE-THREATENING**
Hemorrhage (significant acute blood loss)	NA	Symptomatic AND No transfusion indicated	Symptomatic AND Transfusion of ≤ 2 units packed RBCs (for children ≤ 10 cc/kg) indicated	Life-threatening hypotension OR Transfusion of > 2 units packed RBCs (for children > 10 cc/kg) indicated
Hypertension				
**Adult > 17 years** (with repeat testing at same visit)	> 140 – 159 mmHg systolic OR > 90 – 99 mmHg diastolic	> 160 – 179 mmHg systolic OR > 100 – 109 mmHg diastolic	> 180 mmHg systolic OR > 110 mmHg diastolic	Life-threatening consequences (e.g., malignant hypertension) OR Hospitalization indicated (other than emergency room visit)
**Pediatric ≤ 17 years** (with repeat testing at same visit)	NA	91st – 94th percentile adjusted for age, height, and gender (systolic and/or diastolic)	≥ 95th percentile adjusted for age, height, and gender (systolic and/or diastolic)	Life-threatening consequences (e.g., malignant hypertension) OR Hospitalization indicated (other than emergency room visit)
Hypotension	NA	Symptomatic, corrected with oral fluid replacement	Symptomatic, IV fluids indicated	Shock requiring use of vasopressors or mechanical assistance to maintain blood pressure
Pericardial effusion	Asymptomatic, small effusion requiring no intervention	Asymptomatic, moderate or larger effusion requiring no intervention	Effusion with non-life threatening physiologic consequences OR Effusion with non-urgent intervention indicated	Life-threatening consequences (e.g., tamponade) OR Urgent intervention indicated
Prolonged PR interval				
**Adult > 16 years**	PR interval 0.21 – 0.25 sec	PR interval > 0.25 sec	Type II 2nd degree AV block OR Ventricular pause > 3.0 sec	Complete AV block
**Pediatric ≤ 16 years**	1st degree AV block (PR > normal for age and rate)	Type I 2nd degree AV block	Type II 2nd degree AV block	Complete AV block

Basic Self-care Functions – Adult: Activities such as bathing, dressing, toileting, transfer/movement, continence, and feeding.

Basic Self-care Functions – Young Children: Activities that are age and culturally appropriate (e.g., feeding self with culturally appropriate eating implement).

Usual Social & Functional Activities – Adult: Adaptive tasks and desirable activities, such as going to work, shopping, cooking, use of transportation, pursuing a hobby, etc.

Usual Social & Functional Activities – Young Children: Activities that are age and culturally appropriate (e.g., social interactions, play activities, learning tasks, etc.).

12-28-04 Page 6 of 20 Version 1.0

CLINICAL				
**PARAMETER**	**GRADE 1 MILD**	**GRADE 2 MODERATE**	**GRADE 3 SEVERE**	**GRADE 4 POTENTIALLY LIFE-THREATENING**
Prolonged QTc				
**Adult > 16 years**	Asymptomatic, QTc interval 0.45 – 0.47 sec OR Increase interval < 0.03 sec above baseline	Asymptomatic, QTc interval 0.48 – 0.49 sec OR Increase in interval 0.03 – 0.05 sec above baseline	Asymptomatic, QTc interval ≥ 0.50 sec OR Increase in interval ≥ 0.06 sec above baseline	Life-threatening consequences, e.g. Torsade de pointes or other associated serious ventricular dysrhythmia
**Pediatric ≤ 16 years**	Asymptomatic, QTc interval 0.450 – 0.464 sec	Asymptomatic, QTc interval 0.465 – 0.479 sec	Asymptomatic, QTc interval ≥ 0.480 sec	Life-threatening consequences, e.g. Torsade de pointes or other associated serious ventricular dysrhythmia
Thrombosis/embolism	NA	Deep vein thrombosis AND No intervention indicated (e.g., anticoagulation, lysis filter, invasive procedure)	Deep vein thrombosis AND Intervention indicated (e.g., anticoagulation, lysis filter, invasive procedure)	Embolic event (e.g., pulmonary embolism, life-threatening thrombus)
Vasovagal episode (associated with a procedure of any kind)	Present without loss of consciousness	Present with transient loss of consciousness	NA	NA
Ventricular dysfunction (congestive heart failure)	NA	Asymptomatic diagnostic finding AND intervention indicated	New onset with symptoms OR Worsening symptomatic congestive heart failure	Life-threatening congestive heart failure
**GASTROINTESTINAL**				
Anorexia	Loss of appetite without decreased oral intake	Loss of appetite associated with decreased oral intake without significant weight loss	Loss of appetite associated with significant weight loss	Life-threatening consequences OR Aggressive intervention indicated [e.g., tube feeding or total parenteral nutrition (TPN)]
Ascites	Asymptomatic	Symptomatic AND Intervention indicated (e.g., diuretics or therapeutic paracentesis)	Symptomatic despite intervention	Life-threatening consequences

Basic Self-care Functions – Adult: Activities such as bathing, dressing, toileting, transfer/movement, continence, and feeding.

Basic Self-care Functions – Young Children: Activities that are age and culturally appropriate (e.g., feeding self with culturally appropriate eating implement).

Usual Social & Functional Activities – Adult: Adaptive tasks and desirable activities, such as going to work, shopping, cooking, use of transportation, pursuing a hobby, etc.

Usual Social & Functional Activities – Young Children: Activities that are age and culturally appropriate (e.g., social interactions, play activities, learning tasks, etc.).

12-28-04 Page 7 of 20 Version 1.0

CLINICAL				
**PARAMETER**	**GRADE 1 MILD**	**GRADE 2 MODERATE**	**GRADE 3 SEVERE**	**GRADE 4 POTENTIALLY LIFE-THREATENING**
Cholecystitis	NA	Symptomatic AND Medical intervention indicated	Radiologic, endoscopic, or operative intervention indicated	Life-threatening consequences (e.g., sepsis or perforation)
Constipation	NA	Persistent constipation requiring regular use of dietary modifications, laxatives, or enemas	Obstipation with manual evacuation indicated	Life-threatening consequences (e.g., obstruction)
Diarrhea				
**Adult and Pediatric ≥ 1 year**	Transient or intermittent episodes of unformed stools OR Increase of ≤ 3 stools over baseline per 24-hour period	Persistent episodes of unformed to watery stools OR Increase of 4 – 6 stools over baseline per 24-hour period	Bloody diarrhea OR Increase of ≥ 7 stools per 24-hour period OR IV fluid replacement indicated	Life-threatening consequences (e.g., hypotensive shock)
**Pediatric < 1 year**	Liquid stools (more unformed than usual) but usual number of stools	Liquid stools with increased number of stools OR Mild dehydration	Liquid stools with moderate dehydration	Liquid stools resulting in severe dehydration with aggressive rehydration indicated OR Hypotensive shock
Dysphagia-Odynophagia	Symptomatic but able to eat usual diet	Symptoms causing altered dietary intake without medical intervention indicated	Symptoms causing severely altered dietary intake with medical intervention indicated	Life-threatening reduction in oral intake
Mucositis/stomatitis (clinical exam)<br>Indicate site (e.g., larynx, oral)<br>See Genitourinary for Vulvovaginitis<br>See also Dysphagia-Odynophagia and Proctitis	Erythema of the mucosa	Patchy pseudomembranes or ulcerations	Confluent pseudomembranes or ulcerations OR Mucosal bleeding with minor trauma	Tissue necrosis OR Diffuse spontaneous mucosal bleeding OR Life-threatening consequences (e.g., aspiration, choking)
Nausea	Transient (< 24 hours) or intermittent nausea with no or minimal interference with oral intake	Persistent nausea resulting in decreased oral intake for 24 – 48 hours	Persistent nausea resulting in minimal oral intake for > 48 hours OR Aggressive rehydration indicated (e.g., IV fluids)	Life-threatening consequences (e.g., hypotensive shock)

Basic Self-care Functions – Adult: Activities such as bathing, dressing, toileting, transfer/movement, continence, and feeding.

Basic Self-care Functions – Young Children: Activities that are age and culturally appropriate (e.g., feeding self with culturally appropriate eating implement).

Usual Social & Functional Activities – Adult: Adaptive tasks and desirable activities, such as going to work, shopping, cooking, use of transportation, pursuing a hobby, etc.

Usual Social & Functional Activities – Young Children: Activities that are age and culturally appropriate (e.g., social interactions, play activities, learning tasks, etc.).

Appendix I
Division of Aids Table For Grading the Severity of Adult and Pediatric Adverse Events (cont.)

CLINICAL				
**PARAMETER**	**GRADE 1 MILD**	**GRADE 2 MODERATE**	**GRADE 3 SEVERE**	**GRADE 4 POTENTIALLY LIFE-THREATENING**
Pancreatitis	NA	Symptomatic AND Hospitalization not indicated (other than emergency room visit)	Symptomatic AND Hospitalization indicated (other than emergency room visit)	Life-threatening consequences (e.g., circulatory failure, hemorrhage, sepsis)
Proctitis (functional-symptomatic)<br>Also see Mucositis/stomatitis for clinical exam	Rectal discomfort AND No intervention indicated	Symptoms causing greater than minimal interference with usual social & functional activities OR Medical intervention indicated	Symptoms causing inability to perform usual social & functional activities OR Operative intervention indicated	Life-threatening consequences (e.g., perforation)
Vomiting	Transient or intermittent vomiting with no or minimal interference with oral intake	Frequent episodes of vomiting with no or mild dehydration	Persistent vomiting resulting in orthostatic hypotension OR Aggressive rehydration indicated (e.g., IV fluids)	Life-threatening consequences (e.g., hypotensive shock)
**NEUROLOGIC**				
Alteration in personality-behavior or in mood (e.g., agitation, anxiety, depression, mania, psychosis)	Alteration causing no or minimal interference with usual social & functional activities	Alteration causing greater than minimal interference with usual social & functional activities	Alteration causing inability to perform usual social & functional activities	Behavior potentially harmful to self or others (e.g., suicidal and homicidal ideation or attempt, acute psychosis) OR Causing inability to perform basic self-care functions
Altered Mental Status<br>For Dementia, see Cognitive and behavioral/attentional disturbance (including dementia and attention deficit disorder)	Changes causing no or minimal interference with usual social & functional activities	Mild lethargy or somnolence causing greater than minimal interference with usual social & functional activities	Confusion, memory impairment, lethargy, or somnolence causing inability to perform usual social & functional activities	Delirium OR obtundation, OR coma
Ataxia	Asymptomatic ataxia detectable on exam OR Minimal ataxia causing no or minimal interference with usual social & functional activities	Symptomatic ataxia causing greater than minimal interference with usual social & functional activities	Symptomatic ataxia causing inability to perform usual social & functional activities	Disabling ataxia causing inability to perform basic self-care functions

Basic Self-care Functions – Adult: Activities such as bathing, dressing, toileting, transfer/movement, continence, and feeding.

Basic Self-care Functions – Young Children: Activities that are age and culturally appropriate (e.g., feeding self with culturally appropriate eating implement).

Usual Social & Functional Activities – Adult: Adaptive tasks and desirable activities, such as going to work, shopping, cooking, use of transportation, pursuing a hobby, etc.

Usual Social & Functional Activities – Young Children: Activities that are age and culturally appropriate (e.g., social interactions, play activities, learning tasks, etc.).

Appendix I
Division of Aids Table For Grading the Severity of Adult and Pediatric Adverse Events (cont.)

CLINICAL				
**PARAMETER**	**GRADE 1 MILD**	**GRADE 2 MODERATE**	**GRADE 3 SEVERE**	**GRADE 4 POTENTIALLY LIFE-THREATENING**
Cognitive and behavioral/attentional disturbance (including dementia and attention deficit disorder)	Disability causing no or minimal interference with usual social & functional activities OR Specialized resources not indicated	Disability causing greater than minimal interference with usual social & functional activities OR Specialized resources on part-time basis indicated	Disability causing inability to perform usual social & functional activities OR Specialized resources on a full-time basis indicated	Disability causing inability to perform basic self-care functions OR Institutionalization indicated
CNS ischemia (acute)	NA	NA	Transient ischemic attack	Cerebral vascular accident (CVA, stroke) with neurological deficit
Developmental delay – **Pediatric ≤ 16 years**	Mild developmental delay, either motor or cognitive, as determined by comparison with a developmental screening tool appropriate for the setting	Moderate developmental delay, either motor or cognitive, as determined by comparison with a developmental screening tool appropriate for the setting	Severe developmental delay, either motor or cognitive, as determined by comparison with a developmental screening tool appropriate for the setting	Developmental regression, either motor or cognitive, as determined by comparison with a developmental screening tool appropriate for the setting
Headache	Symptoms causing no or minimal interference with usual social & functional activities	Symptoms causing greater than minimal interference with usual social & functional activities	Symptoms causing inability to perform usual social & functional activities	Symptoms causing inability to perform basic self-care functions OR Hospitalization indicated (other than emergency room visit) OR Headache with significant impairment of alertness or other neurologic function
Insomnia	NA	Difficulty sleeping causing greater than minimal interference with usual social & functional activities	Difficulty sleeping causing inability to perform usual social & functional activities	Disabling insomnia causing inability to perform basic self-care functions
Neuromuscular weakness (including myopathy & neuropathy)	Asymptomatic with decreased strength on exam OR Minimal muscle weakness causing no or minimal interference with usual social & functional activities	Muscle weakness causing greater than minimal interference with usual social & functional activities	Muscle weakness causing inability to perform usual social & functional activities	Disabling muscle weakness causing inability to perform basic self-care functions OR Respiratory muscle weakness impairing ventilation

Basic Self-care Functions – Adult: Activities such as bathing, dressing, toileting, transfer/movement, continence, and feeding.

Basic Self-care Functions – Young Children: Activities that are age and culturally appropriate (e.g., feeding self with culturally appropriate eating implement).

Usual Social & Functional Activities – Adult: Adaptive tasks and desirable activities, such as going to work, shopping, cooking, use of transportation, pursuing a hobby, etc.

Usual Social & Functional Activities – Young Children: Activities that are age and culturally appropriate (e.g., social interactions, play activities, learning tasks, etc.).

12-28-04 Page 10 of 20 Version 1.0

Appendix I
Division of Aids Table For Grading the Severity of Adult and Pediatric Adverse Events (cont.)

CLINICAL				
**PARAMETER**	**GRADE 1 MILD**	**GRADE 2 MODERATE**	**GRADE 3 SEVERE**	**GRADE 4 POTENTIALLY LIFE-THREATENING**
Neurosensory alteration (including paresthesia and painful neuropathy)	Asymptomatic with sensory alteration on exam or minimal paresthesia causing no or minimal interference with usual social & functional activities	Sensory alteration or paresthesia causing greater than minimal interference with usual social & functional activities	Sensory alteration or paresthesia causing inability to perform usual social & functional activities	Disabling sensory alteration or paresthesia causing inability to perform basic self-care functions
Seizure: (new onset) – **Adult ≥ 18 years**<br>See also Seizure: (known pre-existing seizure disorder)	NA	1 seizure	2 – 4 seizures	Seizures of any kind which are prolonged, repetitive (e.g., status epilepticus), or difficult to control (e.g., refractory epilepsy)
Seizure: (known pre-existing seizure disorder) **– Adult ≥ 18 years**<br>For worsening of existing epilepsy the grades should be based on an increase from previous level of control to any of these levels.	NA	Increased frequency of pre-existing seizures (non-repetitive) without change in seizure character OR Infrequent break-through seizures while on stable medication in a previously controlled seizure disorder	Change in seizure character from baseline either in duration or quality (e.g., severity or focality)	Seizures of any kind which are prolonged, repetitive (e.g., status epilepticus), or difficult to control (e.g., refractory epilepsy)
Seizure **– Pediatric < 18 years**	Seizure, generalized onset with or without secondary generalization, lasting < 5 minutes with < 24 hours post ictal state	Seizure, generalized onset with or without secondary generalization, lasting 5 – 20 minutes with < 24 hours post ictal state	Seizure, generalized onset with or without secondary generalization, lasting > 20 minutes	Seizure, generalized onset with or without secondary generalization, requiring intubation and sedation
Syncope (not associated with a procedure)	NA	Present	NA	NA
Vertigo	Vertigo causing no or minimal interference with usual social & functional activities	Vertigo causing greater than minimal interference with usual social & functional activities	Vertigo causing inability to perform usual social & functional activities	Disabling vertigo causing inability to perform basic self-care functions

Basic Self-care Functions – Adult: Activities such as bathing, dressing, toileting, transfer/movement, continence, and feeding.

Basic Self-care Functions – Young Children: Activities that are age and culturally appropriate (e.g., feeding self with culturally appropriate eating implement).

Usual Social & Functional Activities – Adult: Adaptive tasks and desirable activities, such as going to work, shopping, cooking, use of transportation, pursuing a hobby, etc.

Usual Social & Functional Activities – Young Children: Activities that are age and culturally appropriate (e.g., social interactions, play activities, learning tasks, etc.).

12-28-04 Page 11 of 20 Version 1.0

Appendix I
Division of Aids Table For Grading the Severity of Adult and Pediatric Adverse Events (cont.)

CLINICAL					
**PARAMETER**		**GRADE 1 MILD**	**GRADE 2 MODERATE**	**GRADE 3 SEVERE**	**GRADE 4 POTENTIALLY LIFE-THREATENING**
**RESPIRATORY**					
Bronchospasm (acute)		FEV1 or peak flow reduced to 70 – 80%	FEV1 or peak flow 50 – 69%	FEV1 or peak flow 25 – 49%	Cyanosis OR FEV1 or peak flow < 25% OR Intubation
Dyspnea or respiratory distress					
	**Adult ≥ 14 years**	Dyspnea on exertion with no or minimal interference with usual social & functional activities	Dyspnea on exertion causing greater than minimal interference with usual social & functional activities	Dyspnea at rest causing inability to perform usual social & functional activities	Respiratory failure with ventilatory support indicated
	**Pediatric < 14 years**	Wheezing OR minimal increase in respiratory rate for age	Nasal flaring OR Intercostal retractions OR Pulse oximetry 90 – 95%	Dyspnea at rest causing inability to perform usual social & functional activities OR Pulse oximetry < 90%	Respiratory failure with ventilatory support indicated
**MUSCULOSKELETAL**					
Arthralgia<br>See also Arthritis		Joint pain causing no or minimal interference with usual social & functional activities	Joint pain causing greater than minimal interference with usual social & functional activities	Joint pain causing inability to perform usual social & functional activities	Disabling joint pain causing inability to perform basic self-care functions
Arthritis<br>See also Arthralgia		Stiffness or joint swelling causing no or minimal interference with usual social & functional activities	Stiffness or joint swelling causing greater than minimal interference with usual social & functional activities	Stiffness or joint swelling causing inability to perform usual social & functional activities	Disabling joint stiffness or swelling causing inability to perform basic self-care functions
Bone Mineral Loss					
	**Adult ≥ 21 years**	BMD t-score -2.5 to -1.0	BMD t-score < -2.5	Pathological fracture (including loss of vertebral height)	Pathologic fracture causing life-threatening consequences
	**Pediatric < 21 years**	BMD z-score -2.5 to -1.0	BMD z-score < -2.5	Pathological fracture (including loss of vertebral height)	Pathologic fracture causing life-threatening consequences

Basic Self-care Functions – Adult: Activities such as bathing, dressing, toileting, transfer/movement, continence, and feeding.

Basic Self-care Functions – Young Children: Activities that are age and culturally appropriate (e.g., feeding self with culturally appropriate eating implement).

Usual Social & Functional Activities – Adult: Adaptive tasks and desirable activities, such as going to work, shopping, cooking, use of transportation, pursuing a hobby, etc.

Usual Social & Functional Activities – Young Children: Activities that are age and culturally appropriate (e.g., social interactions, play activities, learning tasks, etc.).

Appendix I
Division of Aids Table For Grading the Severity of Adult and Pediatric Adverse Events (cont.)

CLINICAL				
**PARAMETER**	**GRADE 1 MILD**	**GRADE 2 MODERATE**	**GRADE 3 SEVERE**	**GRADE 4 POTENTIALLY LIFE-THREATENING**
Myalgia (non-injection site)	Muscle pain causing no or minimal interference with usual social & functional activities	Muscle pain causing greater than minimal interference with usual social & functional activities	Muscle pain causing inability to perform usual social & functional activities	Disabling muscle pain causing inability to perform basic self-care functions
Osteonecrosis	NA	Asymptomatic with radiographic findings AND No operative intervention indicated	Symptomatic bone pain with radiographic findings OR Operative intervention indicated	Disabling bone pain with radiographic findings causing inability to perform basic self-care functions
**GENITOURINARY**				
Cervicitis (symptoms) (For use in studies evaluating topical study agents) For other cervicitis see Infection: Infection (any other than HIV infection)	Symptoms causing no or minimal interference with usual social & functional activities	Symptoms causing greater than minimal interference with usual social & functional activities	Symptoms causing inability to perform usual social & functional activities	Symptoms causing inability to perform basic self-care functions
Cervicitis (clinical exam) (For use in studies evaluating topical study agents) For other cervicitis see Infection: Infection (any other than HIV infection)	Minimal cervical abnormalities on examination (erythema, mucopurulent discharge, or friability) OR Epithelial disruption < 25% of total surface	Moderate cervical abnormalities on examination (erythema, mucopurulent discharge, or friability) OR Epithelial disruption of 25 – 49% total surface	Severe cervical abnormalities on examination (erythema, mucopurulent discharge, or friability) OR Epithelial disruption 50 – 75% total surface	Epithelial disruption > 75% total surface
Inter-menstrual bleeding (IMB)	Spotting observed by participant OR Minimal blood observed during clinical or colposcopic examination	Inter-menstrual bleeding not greater in duration or amount than usual menstrual cycle	Inter-menstrual bleeding greater in duration or amount than usual menstrual cycle	Hemorrhage with life-threatening hypotension OR Operative intervention indicated
Urinary tract obstruction (e.g., stone)	NA	Signs or symptoms of urinary tract obstruction without hydronephrosis or renal dysfunction	Signs or symptoms of urinary tract obstruction with hydronephrosis or renal dysfunction	Obstruction causing life-threatening consequences

Basic Self-care Functions – Adult: Activities such as bathing, dressing, toileting, transfer/movement, continence, and feeding.

Basic Self-care Functions – Young Children: Activities that are age and culturally appropriate (e.g., feeding self with culturally appropriate eating implement).

Usual Social & Functional Activities – Adult: Adaptive tasks and desirable activities, such as going to work, shopping, cooking, use of transportation, pursuing a hobby, etc.

Usual Social & Functional Activities – Young Children: Activities that are age and culturally appropriate (e.g., social interactions, play activities, learning tasks, etc.).

12-28-04 Page 13 of 20 Version 1.0

Appendix I
Division of Aids Table For Grading the Severity of Adult and Pediatric Adverse Events (cont.)

CLINICAL				
PARAMETER	GRADE 1 MILD	GRADE 2 MODERATE	GRADE 3 SEVERE	GRADE 4 POTENTIALLY LIFE-THREATENING
Vulvovaginitis (symptoms) (Use in studies evaluating topical study agents) For other vulvovaginitis see Infection: Infection (any other than HIV infection)	Symptoms causing no or minimal interference with usual social & functional activities	Symptoms causing greater than minimal interference with usual social & functional activities	Symptoms causing inability to perform usual social & functional activities	Symptoms causing inability to perform basic self-care functions
Vulvovaginitis (clinical exam) (Use in studies evaluating topical study agents) For other vulvovaginitis see Infection: Infection (any other than HIV infection)	Minimal vaginal abnormalities on examination OR Epithelial disruption < 25% of total surface	Moderate vaginal abnormalities on examination OR Epithelial disruption of 25 - 49% total surface	Severe vaginal abnormalities on examination OR Epithelial disruption 50 - 75% total surface	Vaginal perforation OR Epithelial disruption > 75% total surface
**OCULAR/VISUAL**				
Uveitis	Asymptomatic but detectable on exam	Symptomatic anterior uveitis OR Medical intervention indicated	Posterior or pan-uveitis OR Operative intervention indicated	Disabling visual loss in affected eye(s)
Visual changes (from baseline)	Visual changes causing no or minimal interference with usual social & functional activities	Visual changes causing greater than minimal interference with usual social & functional activities	Visual changes causing inability to perform usual social & functional activities	Disabling visual loss in affected eye(s)
**ENDOCRINE/METABOLIC**				
Abnormal fat accumulation (e.g., back of neck, breasts, abdomen)	Detectable by study participant (or by caregiver for young children and disabled adults)	Detectable on physical exam by health care provider	Disfiguring OR Obvious changes on casual visual inspection	NA

Basic Self-care Functions – Adult: Activities such as bathing, dressing, toileting, transfer/movement, continence, and feeding.

Basic Self-care Functions – Young Children: Activities that are age and culturally appropriate (e.g., feeding self with culturally appropriate eating implement).

Usual Social & Functional Activities – Adult: Adaptive tasks and desirable activities, such as going to work, shopping, cooking, use of transportation, pursuing a hobby, etc.

Usual Social & Functional Activities – Young Children: Activities that are age and culturally appropriate (e.g., social interactions, play activities, learning tasks, etc.).

12-28-04 Page 14 of 20 Version 1.0

Appendix I
Division of Aids Table For Grading the Severity of Adult and Pediatric Adverse Events (cont.)

CLINICAL				
PARAMETER	GRADE 1 MILD	GRADE 2 MODERATE	GRADE 3 SEVERE	GRADE 4 POTENTIALLY LIFE-THREATENING
Diabetes mellitus	NA	New onset without need to initiate medication OR Modification of current medications to regain glucose control	New onset with initiation of medication indicated OR Diabetes uncontrolled despite treatment modification	Life-threatening consequences (e.g., ketoacidosis, hyperosmolar non-ketotic coma)
Gynecomastia	Detectable by study participant or caregiver (for young children and disabled adults)	Detectable on physical exam by health care provider	Disfiguring OR Obvious on casual visual inspection	NA
Hyperthyroidism	Asymptomatic	Symptomatic causing greater than minimal interference with usual social & functional activities OR Thyroid suppression therapy indicated	Symptoms causing inability to perform usual social & functional activities OR Uncontrolled despite treatment modification	Life-threatening consequences (e.g., thyroid storm)
Hypothyroidism	Asymptomatic	Symptomatic causing greater than minimal interference with usual social & functional activities OR Thyroid replacement therapy indicated	Symptoms causing inability to perform usual social & functional activities OR Uncontrolled despite treatment modification	Life-threatening consequences (e.g., myxedema coma)
Lipoatrophy (e.g., fat loss from the face, extremities, buttocks)	Detectable by study participant (or by caregiver for young children and disabled adults)	Detectable on physical exam by health care provider	Disfiguring OR Obvious on casual visual inspection	NA

Basic Self-care Functions – Adult: Activities such as bathing, dressing, toileting, transfer/movement, continence, and feeding.

Basic Self-care Functions – Young Children: Activities that are age and culturally appropriate (e.g., feeding self with culturally appropriate eating implement).

Usual Social & Functional Activities – Adult: Adaptive tasks and desirable activities, such as going to work, shopping, cooking, use of transportation, pursuing a hobby, etc.

Usual Social & Functional Activities – Young Children: Activities that are age and culturally appropriate (e.g., social interactions, play activities, learning tasks, etc.).

12-28-04 Page 15 of 20 Version 1.0

Appendix I
Division of Aids Table For Grading the Severity of Adult and Pediatric Adverse Events (cont.)

LABORATORY				
**PARAMETER**	**GRADE 1**<br>**MILD**	**GRADE 2**<br>**MODERATE**	**GRADE 3**<br>**SEVERE**	**GRADE 4**<br>**POTENTIALLY LIFE-THREATENING**
**HEMATOLOGY**	***Standard International Units are listed in italics***			
Absolute CD4+ count<br>**– Adult and Pediatric > 13 years**<br>(HIV NEGATIVE ONLY)	300 – 400/mm^3<br>*300 – 400/µL*	200 – 299/mm^3<br>*200 – 299/µL*	100 – 199/mm^3<br>*100 – 199/µL*	< 100/mm^3<br>*< 100/µL*
Absolute lymphocyte count<br>**– Adult and Pediatric > 13 years**<br>(HIV NEGATIVE ONLY)	600 – 650/mm^3<br>*0.600 x 10^9 – 0.650 x 10^9/L*	500 – 599/mm^3<br>*0.500 x 10^9 – 0.599 x 10^9/L*	350 – 499/mm^3<br>*0.350 x 10^9 – 0.499 x 10^9/L*	< 350/mm^3<br>*< 0.350 x 10^9/L*
Absolute neutrophil count (ANC)				
**Adult and Pediatric, > 7 days**	1,000 – 1,300/mm^3<br>*1.000 x 10^9 – 1.300 x 10^9/L*	750 – 999/mm^3<br>*0.750 x 10^9 – 0.999 x 10^9/L*	500 – 749/mm^3<br>*0.500 x 10^9 – 0.749 x 10^9/L*	< 500/mm^3<br>*< 0.500 x 10^9/L*
**Infant[*,†], 2 – ≤ 7 days**	1,250 – 1,500/mm^3<br>*1.250 x 10^9 – 1.500 x 10^9/L*	1,000 – 1,249/mm^3<br>*1.000 x 10^9 – 1.249 x 10^9/L*	750 – 999/mm^3<br>*0.750 x 10^9 – 0.999 x 10^9/L*	< 750/mm^3<br>*< 0.750 x 10^9/L*
**Infant[*,†], 1 day**	4,000 – 5,000/mm^3<br>*4.000 x 10^9 – 5.000 x 10^9/L*	3,000 – 3,999/mm^3<br>*3.000 x 10^9 – 3.999 x10^9/L*	1,500 – 2,999/mm^3<br>*1.500 x 10^9 – 2.999 x 10^9/L*	< 1,500/mm^3<br>*< 1.500 x 10^9/L*
Fibrinogen, decreased	100 – 200 mg/dL<br>*1.00 – 2.00 g/L*<br>OR<br>0.75 – 0.99 x LLN	75 – 99 mg/dL<br>*0.75 – 0.99 g/L*<br>OR<br>0.50 – 0.74 x LLN	50 – 74 mg/dL<br>*0.50 – 0.74 g/L*<br>OR<br>0.25 – 0.49 x LLN	< 50 mg/dL<br>*< 0.50 g/L*<br>OR<br>< 0.25 x LLN<br>OR<br>Associated with gross bleeding
Hemoglobin (Hgb)				
**Adult and Pediatric ≥ 57 days**<br>(HIV POSITIVE ONLY)	8.5 – 10.0 g/dL<br>*1.32 – 1.55 mmol/L*	7.5 – 8.4 g/dL<br>*1.16 – 1.31 mmol/L*	6.50 – 7.4 g/dL<br>*1.01 – 1.15 mmol/L*	< 6.5 g/dL<br>*< 1.01 mmol/L*
**Adult and Pediatric ≥ 57 days**<br>(HIV NEGATIVE ONLY)	10.0 – 10.9 g/dL<br>*1.55 – 1.69 mmol/L*<br>OR<br>Any decrease<br>2.5 – 3.4 g/dL<br>*0.39 – 0.53 mmol/L*	9.0 – 9.9 g/dL<br>*1.40 – 1.54 mmol/L*<br>OR<br>Any decrease<br>3.5 – 4.4 g/dL<br>*0.54 – 0.68 mmol/L*	7.0 – 8.9 g/dL<br>*1.09 – 1.39 mmol/L*<br>OR<br>Any decrease<br>≥ 4.5 g/dL<br>*≥ 0.69 mmol/L*	< 7.0 g/dL<br>*< 1.09 mmol/L*
**Infant[*,†], 36 – 56 days**<br>(HIV POSITIVE OR NEGATIVE)	8.5 – 9.4 g/dL<br>*1.32 – 1.46 mmol/L*	7.0 – 8.4 g/dL<br>*1.09 – 1.31 mmol/L*	6.0 – 6.9 g/dL<br>*0.93 – 1.08 mmol/L*	< 6.00 g/dL<br>*< 0.93 mmol/L*

[*] Values are for term infants.

[†] Use age and sex appropriate values (e.g., bilirubin), including preterm infants.

Appendix I
Division of Aids Table For Grading the Severity of Adult and Pediatric Adverse Events (cont.)

LABORATORY				
**PARAMETER**	**GRADE 1 MILD**	**GRADE 2 MODERATE**	**GRADE 3 SEVERE**	**GRADE 4 POTENTIALLY LIFE-THREATENING**
**Infant[*,†], 22 – 35 days** (HIV POSITIVE OR NEGATIVE)	9.5 – 10.5 g/dL<br>*1.47 – 1.63 mmol/L*	8.0 – 9.4 g/dL<br>*1.24 – 1.46 mmol/L*	7.0 – 7.9 g/dL<br>*1.09 – 1.23 mmol/L*	< 7.00 g/dL<br>*< 1.09 mmol/L*
**Infant[*,†], 1 – 21 days** (HIV POSITIVE OR NEGATIVE)	12.0 – 13.0 g/dL<br>*1.86 – 2.02 mmol/L*	10.0 – 11.9 g/dL<br>*1.55 – 1.85 mmol/L*	9.0 – 9.9 g/dL<br>*1.40 – 1.54 mmol/L*	< 9.0 g/dL<br>*< 1.40 mmol/L*
International Normalized Ratio of prothrombin time (INR)	1.1 – 1.5 x ULN	1.6 – 2.0 x ULN	2.1 – 3.0 x ULN	> 3.0 x ULN
Methemoglobin	5.0 – 10.0%	10.1 – 15.0%	15.1 – 20.0%	> 20.0%
Prothrombin Time (PT)	1.1 – 1.25 x ULN	1.26 – 1.50 x ULN	1.51 – 3.00 x ULN	> 3.00 x ULN
Partial Thromboplastin Time (PTT)	1.1 – 1.66 x ULN	1.67 – 2.33 x ULN	2.34 – 3.00 x ULN	> 3.00 x ULN
Platelets, decreased	100,000 – 124,999/mm^3<br>*100.000 x 10^9 – 124.999 x 10^9/L*	50,000 – 99,999/mm^3<br>*50.000 x 10^9 – 99.999 x 10^9/L*	25,000 – 49,999/mm^3<br>*25.000 x 10^9 – 49.999 x 10^9/L*	< 25,000/mm^3<br>*< 25.000 x 10^9/L*
WBC, decreased	2,000 – 2,500/mm^3<br>*2.000 x 10^9 – 2.500 x 10^9/L*	1,500 – 1,999/mm^3<br>*1.500 x 10^9 – 1.999 x 10^9/L*	1,000 – 1,499/mm^3<br>*1.000 x 10^9 – 1.499 x 10^9/L*	< 1,000/mm^3<br>*< 1.000 x 10^9/L*
**CHEMISTRIES**	***Standard International Units are listed in italics***			
Acidosis	NA	pH < normal, but ≥ 7.3	pH < 7.3 without life-threatening consequences	pH < 7.3 with life-threatening consequences
Albumin, serum, low	3.0 g/dL – < LLN<br>*30 g/L – < LLN*	2.0 – 2.9 g/dL<br>*20 – 29 g/L*	< 2.0 g/dL<br>*< 20 g/L*	NA
Alkaline Phosphatase	1.25 – 2.5 x ULN[†]	2.6 – 5.0 x ULN[†]	5.1 – 10.0 x ULN[†]	> 10.0 x ULN[†]
Alkalosis	NA	pH > normal, but ≤ 7.5	pH > 7.5 without life-threatening consequences	pH > 7.5 with life-threatening consequences
ALT (SGPT)	1.25 – 2.5 x ULN	2.6 – 5.0 x ULN	5.1 – 10.0 x ULN	> 10.0 x ULN
AST (SGOT)	1.25 – 2.5 x ULN	2.6 – 5.0 x ULN	5.1 – 10.0 x ULN	> 10.0 x ULN
Bicarbonate, serum, low	16.0 mEq/L – < LLN<br>*16.0 mmol/L – < LLN*	11.0 – 15.9 mEq/L<br>*11.0 – 15.9 mmol/L*	8.0 – 10.9 mEq/L<br>*8.0 – 10.9 mmol/L*	< 8.0 mEq/L<br>*< 8.0 mmol/L*
Bilirubin (Total)				
**Adult and Pediatric > 14 days**	1.1 – 1.5 x ULN	1.6 – 2.5 x ULN	2.6 – 5.0 x ULN	> 5.0 x ULN

[*] Values are for term infants.

[†] Use age and sex appropriate values (e.g., bilirubin), including preterm infants.

12-28-04 Page 17 of 20 Version 1.0

Appendix I
Division of Aids Table For Grading the Severity of Adult and Pediatric Adverse Events (cont.)

LABORATORY					
**PARAMETER**		**GRADE 1 MILD**	**GRADE 2 MODERATE**	**GRADE 3 SEVERE**	**GRADE 4 POTENTIALLY LIFE-THREATENING**
	**Infant*[†], ≤ 14 days** (non-hemolytic)	NA	20.0 – 25.0 mg/dL *342 – 428 µmol/L*	25.1 – 30.0 mg/dL *429 – 513 µmol/L*	> 30.0 mg/dL *> 513.0 µmol/L*
	**Infant*[†], ≤ 14 days** (hemolytic)	NA	NA	20.0 – 25.0 mg/dL *342 – 428 µmol/L*	> 25.0 mg/dL *> 428 µmol/L*
Calcium, serum, high (corrected for albumin)					
	**Adult and Pediatric ≥ 7 days**	10.6 – 11.5 mg/dL *2.65 – 2.88 mmol/L*	11.6 – 12.5 mg/dL *2.89 – 3.13 mmol/L*	12.6 – 13.5 mg/dL *3.14 – 3.38 mmol/L*	> 13.5 mg/dL *> 3.38 mmol/L*
	**Infant*[†], < 7 days**	11.5 – 12.4 mg/dL *2.88 – 3.10 mmol/L*	12.5 – 12.9 mg/dL *3.11 – 3.23 mmol/L*	13.0 – 13.5 mg/dL *3.245 – 3.38 mmol/L*	> 13.5 mg/dL *> 3.38 mmol/L*
Calcium, serum, low (corrected for albumin)					
	**Adult and Pediatric ≥ 7 days**	7.8 – 8.4 mg/dL *1.95 – 2.10 mmol/L*	7.0 – 7.7 mg/dL *1.75 – 1.94 mmol/L*	6.1 – 6.9 mg/dL *1.53 – 1.74 mmol/L*	< 6.1 mg/dL *< 1.53 mmol/L*
	**Infant*[†], < 7 days**	6.5 – 7.5 mg/dL *1.63 – 1.88 mmol/L*	6.0 – 6.4 mg/dL *1.50 – 1.62 mmol/L*	5.50 – 5.90 mg/dL *1.38 – 1.51 mmol/L*	< 5.50 mg/dL *< 1.38 mmol/L*
Cardiac troponin I (cTnI)		NA	NA	NA	Levels consistent with myocardial infarction or unstable angina as defined by the manufacturer
Cardiac troponin T (cTnT)		NA	NA	NA	≥ 0.20 ng/mL OR Levels consistent with myocardial infarction or unstable angina as defined by the manufacturer
Cholesterol (fasting)					
	**Adult ≥ 18 years**	200 – 239 mg/dL *5.18 – 6.19 mmol/L*	240 – 300 mg/dL *6.20 – 7.77 mmol/L*	> 300 mg/dL *> 7.77 mmol/L*	NA
	**Pediatric < 18 years**	170 – 199 mg/dL *4.40 – 5.15 mmol/L*	200 – 300 mg/dL *5.16 – 7.77 mmol/L*	> 300 mg/dL *> 7.77 mmol/L*	NA
Creatine Kinase		3.0 – 5.9 x ULN[†]	6.0 – 9.9 x ULN[†]	10.0 – 19.9 x ULN[†]	≥ 20.0 x ULN[†]
Creatinine		1.1 – 1.3 x ULN[†]	1.4 – 1.8 x ULN[†]	1.9 – 3.4 x ULN[†]	≥ 3.5 x ULN[†]
Glucose, serum, high					
	Nonfasting	116 – 160 mg/dL *6.44 – 8.88 mmol/L*	161 – 250 mg/dL *8.89 – 13.88 mmol/L*	251 – 500 mg/dL *13.89 – 27.75 mmol/L*	> 500 mg/dL *> 27.75 mmol/L*
	Fasting	110 – 125 mg/dL *6.11 – 6.94 mmol/L*	126 – 250 mg/dL *6.95 – 13.88 mmol/L*	251 – 500 mg/dL *13.89 – 27.75 mmol/L*	> 500 mg/dL *> 27.75 mmol/L*

* Values are for term infants.

† Use age and sex appropriate values (e.g., bilirubin), including preterm infants.

Appendix I
Division of Aids Table For Grading the Severity of Adult and Pediatric Adverse Events (cont.)

LABORATORY				
**PARAMETER**	**GRADE 1 MILD**	**GRADE 2 MODERATE**	**GRADE 3 SEVERE**	**GRADE 4 POTENTIALLY LIFE-THREATENING**
Glucose, serum, low				
**Adult and Pediatric ≥ 1 month**	55 – 64 mg/dL<br>3.05 – 3.55 mmol/L	40 – 54 mg/dL<br>2.22 – 3.06 mmol/L	30 – 39 mg/dL<br>1.67 – 2.23 mmol/L	< 30 mg/dL<br>< 1.67 mmol/L
**Infant[*,†], < 1 month**	50 – 54 mg/dL<br>2.78 – 3.00 mmol/L	40 – 49 mg/dL<br>2.22 – 2.77 mmol/L	30 – 39 mg/dL<br>1.67 – 2.21 mmol/L	< 30 mg/dL<br>< 1.67 mmol/L
Lactate	< 2.0 x ULN without acidosis	≥ 2.0 x ULN without acidosis	Increased lactate with pH < 7.3 without life-threatening consequences	Increased lactate with pH < 7.3 with life-threatening consequences
LDL cholesterol (fasting)				
**Adult ≥ 18 years**	130 – 159 mg/dL<br>3.37 – 4.12 mmol/L	160 – 190 mg/dL<br>4.13 – 4.90 mmol/L	≥ 190 mg/dL<br>≥ 4.91 mmol/L	NA
**Pediatric > 2 - < 18 years**	110 – 129 mg/dL<br>2.85 – 3.34 mmol/L	130 – 189 mg/dL<br>3.35 – 4.90 mmol/L	≥ 190 mg/dL<br>≥ 4.91 mmol/L	NA
Lipase	1.1 – 1.5 x ULN	1.6 – 3.0 x ULN	3.1 – 5.0 x ULN	> 5.0 x ULN
Magnesium, serum, low	1.2 – 1.4 mEq/L<br>0.60 – 0.70 mmol/L	0.9 – 1.1 mEq/L<br>0.45 – 0.59 mmol/L	0.6 – 0.8 mEq/L<br>0.30 – 0.44 mmol/L	< 0.60 mEq/L<br>< 0.30 mmol/L
Pancreatic amylase	1.1 – 1.5 x ULN	1.6 – 2.0 x ULN	2.1 – 5.0 x ULN	> 5.0 x ULN
Phosphate, serum, low				
**Adult and Pediatric > 14 years**	2.5 mg/dL – < LLN<br>0.81 mmol/L – < LLN	2.0 – 2.4 mg/dL<br>0.65 – 0.80 mmol/L	1.0 – 1.9 mg/dL<br>0.32 – 0.64 mmol/L	< 1.00 mg/dL<br>< 0.32 mmol/L
**Pediatric 1 year – 14 years**	3.0 – 3.5 mg/dL<br>0.97 – 1.13 mmol/L	2.5 – 2.9 mg/dL<br>0.81 – 0.96 mmol/L	1.5 – 2.4 mg/dL<br>0.48 – 0.80 mmol/L	< 1.50 mg/dL<br>< 0.48 mmol/L
**Pediatric < 1 year**	3.5 – 4.5 mg/dL<br>1.13 – 1.45 mmol/L	2.5 – 3.4 mg/dL<br>0.81 – 1.12 mmol/L	1.5 – 2.4 mg/dL<br>0.48 – 0.80 mmol/L	< 1.50 mg/dL<br>< 0.48 mmol/L
Potassium, serum, high	5.6 – 6.0 mEq/L<br>5.6 – 6.0 mmol/L	6.1 – 6.5 mEq/L<br>6.1 – 6.5 mmol/L	6.6 – 7.0 mEq/L<br>6.6 – 7.0 mmol/L	> 7.0 mEq/L<br>> 7.0 mmol/L
Potassium, serum, low	3.0 – 3.4 mEq/L<br>3.0 – 3.4 mmol/L	2.5 – 2.9 mEq/L<br>2.5 – 2.9 mmol/L	2.0 – 2.4 mEq/L<br>2.0 – 2.4 mmol/L	< 2.0 mEq/L<br>< 2.0 mmol/L
Sodium, serum, high	146 – 150 mEq/L<br>146 – 150 mmol/L	151 – 154 mEq/L<br>151 – 154 mmol/L	155 – 159 mEq/L<br>155 – 159 mmol/L	≥ 160 mEq/L<br>≥ 160 mmol/L
Sodium, serum, low	130 – 135 mEq/L<br>130 – 135 mmol/L	125 – 129 mEq/L<br>125 – 129 mmol/L	121 – 124 mEq/L<br>121 – 124 mmol/L	≤ 120 mEq/L<br>≤ 120 mmol/L
Triglycerides (fasting)	NA	500 – 750 mg/dL<br>5.65 – 8.48 mmol/L	751 – 1,200 mg/dL<br>8.49 – 13.56 mmol/L	> 1,200 mg/dL<br>> 13.56 mmol/L
Uric acid	7.5 – 10.0 mg/dL<br>0.45 – 0.59 mmol/L	10.1 – 12.0 mg/dL<br>0.60 – 0.71 mmol/L	12.1 – 15.0 mg/dL<br>0.72 – 0.89 mmol/L	> 15.0 mg/dL<br>> 0.89 mmol/L

[*] Values are for term infants.

[†] Use age and sex appropriate values (e.g., bilirubin), including preterm infants.

12-28-04 Page 19 of 20 Version 1.0

Appendix I
Division of Aids Table For Grading the Severity of Adult and Pediatric Adverse Events (cont.)

LABORATORY					
PARAMETER		GRADE 1 MILD	GRADE 2 MODERATE	GRADE 3 SEVERE	GRADE 4 POTENTIALLY LIFE-THREATENING
**URINALYSIS**		***Standard International Units are listed in italics***			
Hematuria (microscopic)		6 – 10 RBC/HPF	> 10 RBC/HPF	Gross, with or without clots OR with RBC casts	Transfusion indicated
Proteinuria, random collection		1 +	2 – 3 +	4 +	NA
Proteinuria, 24 hour collection					
	**Adult and Pediatric ≥ 10 years**	200 – 999 mg/24 h<br>*0.200 – 0.999 g/d*	1,000 – 1,999 mg/24 h<br>*1.000 – 1.999 g/d*	2,000 – 3,500 mg/24 h<br>*2.000 – 3.500 g/d*	> 3,500 mg/24 h<br>*> 3.500 g/d*
	**Pediatric > 3 mo - < 10 years**	201 – 499 mg/m²/24 h<br>*0.201 – 0.499 g/d*	500 – 799 mg/m²/24 h<br>*0.500 – 0.799 g/d*	800 – 1,000 mg/m²/24 h<br>*0.800 – 1.000 g/d*	> 1,000 mg/ m²/24 h<br>*> 1.000 g/d*

* Values are for term infants.

† Use age and sex appropriate values (e.g., bilirubin), including preterm infants.

12-28-04 Page 20 of 20 Version 1.0

Reference:

[NIAID] National Institute of Allergy and Infectious Diseases. Division of AIDS. Clinical Research Policies and Standard Procedures Documents. Division of AIDS table for grading the severity of adult and pediatric adverse events. [resource on the Internet]. 2004. Available from: http://www.niaid.nih.gov/LabsAndResources/resources/DAIDSClinRsrch/Documents/daidsaegradingtable.pdf.
